# Effect of Pain Reprocessing Therapy vs Placebo and Usual Care for Patients With Chronic Back Pain: A Randomized Clinical Trial

**DOI:** 10.1001/jamapsychiatry.2021.2669

**Published:** 2021-09-29

**Authors:** Yoni K. Ashar, Alan Gordon, Howard Schubiner, Christie Uipi, Karen Knight, Zachary Anderson, Judith Carlisle, Laurie Polisky, Stephan Geuter, Thomas F. Flood, Philip A. Kragel, Sona Dimidjian, Mark A. Lumley, Tor D. Wager

**Affiliations:** 1 Department of Psychiatry, Weill Cornell Medical College, New York City, New York; 2 Department of Psychology and Neuroscience, University of Colorado, Boulder; 3 Institute of Cognitive Science, University of Colorado, Boulder; 4 Pain Psychology Center, Los Angeles, California; 5 Ascension Providence Hospital, Southfield, Michigan; 6 Michigan State University College of Human Medicine, East Lansing; 7 Panorama Orthopedics and Spine Center, Golden, Colorado; 8 Department of Psychology, Northwestern University, Evanston, Illinois; 9 Department of Philosophy, Washington University in Saint Louis, Saint Louis, Missouri; 10 Johns Hopkins University Department of Biostatistics, Baltimore, Maryland; 11 Department of Radiology, Brigham and Women’s Hospital, Boston, Massachusetts; 12 Department of Psychology, Emory University, Atlanta, Georgia; 13 Renée Crown Wellness Institute, University of Colorado, Boulder; 14 Department of Psychology, Wayne State University, Detroit, Michigan; 15 Department of Psychological and Brain Sciences, Dartmouth College, Hanover, New Hampshire

## Abstract

**Importance:**

Chronic back pain (CBP) is a leading cause of disability, and treatment is often ineffective. Approximately 85% of cases are primary CBP, for which peripheral etiology cannot be identified, and maintenance factors include fear, avoidance, and beliefs that pain indicates injury.

**Objective:**

To test whether a psychological treatment (pain reprocessing therapy [PRT]) aiming to shift patients’ beliefs about the causes and threat value of pain provides substantial and durable pain relief from primary CBP and to investigate treatment mechanisms.

**Design, Setting, and Participants:**

This randomized clinical trial with longitudinal functional magnetic resonance imaging (fMRI) and 1-year follow-up assessment was conducted in a university research setting from November 2017 to August 2018, with 1-year follow-up completed by November 2019. Clinical and fMRI data were analyzed from January 2019 to August 2020. The study compared PRT with an open-label placebo treatment and with usual care in a community sample.

**Interventions:**

Participants randomized to PRT participated in 1 telehealth session with a physician and 8 psychological treatment sessions over 4 weeks. Treatment aimed to help patients reconceptualize their pain as due to nondangerous brain activity rather than peripheral tissue injury, using a combination of cognitive, somatic, and exposure-based techniques. Participants randomized to placebo received an open-label subcutaneous saline injection in the back; participants randomized to usual care continued their routine, ongoing care.

**Main Outcomes and Measures:**

One-week mean back pain intensity score (0 to 10) at posttreatment, pain beliefs, and fMRI measures of evoked pain and resting connectivity.

**Results:**

At baseline, 151 adults (54% female; mean [SD] age, 41.1 [15.6] years) reported mean (SD) pain of low to moderate severity (mean [SD] pain intensity, 4.10 [1.26] of 10; mean [SD] disability, 23.34 [10.12] of 100) and mean (SD) pain duration of 10.0 (8.9) years. Large group differences in pain were observed at posttreatment, with a mean (SD) pain score of 1.18 (1.24) in the PRT group, 2.84 (1.64) in the placebo group, and 3.13 (1.45) in the usual care group. Hedges *g* was −1.14 for PRT vs placebo and −1.74 for PRT vs usual care (*P* < .001). Of 151 total participants, 33 of 50 participants (66%) randomized to PRT were pain-free or nearly pain-free at posttreatment (reporting a pain intensity score of 0 or 1 of 10), compared with 10 of 51 participants (20%) randomized to placebo and 5 of 50 participants (10%) randomized to usual care. Treatment effects were maintained at 1-year follow-up, with a mean (SD) pain score of 1.51 (1.59) in the PRT group, 2.79 (1.78) in the placebo group, and 3.00 (1.77) in the usual care group. Hedges *g* was −0.70 for PRT vs placebo (*P* = .001) and −1.05 for PRT vs usual care (*P* < .001) at 1-year follow-up. Longitudinal fMRI showed (1) reduced responses to evoked back pain in the anterior midcingulate and the anterior prefrontal cortex for PRT vs placebo; (2) reduced responses in the anterior insula for PRT vs usual care; (3) increased resting connectivity from the anterior prefrontal cortex and the anterior insula to the primary somatosensory cortex for PRT vs both control groups; and (4) increased connectivity from the anterior midcingulate to the precuneus for PRT vs usual care.

**Conclusions and Relevance:**

Psychological treatment centered on changing patients’ beliefs about the causes and threat value of pain may provide substantial and durable pain relief for people with CBP.

**Trial Registration:**

ClinicalTrials.gov Identifier: NCT03294148.

## Key Points

**Question:**

Can a psychological treatment based on the reappraisal of primary chronic back pain as due to nondangerous central nervous system processes provide substantial and durable pain relief?

**Findings:**

In this randomized clinical trial, 33 of 50 participants (66%) randomized to 4 weeks of pain reprocessing therapy were pain-free or nearly pain-free at posttreatment, compared with 10 of 51 participants (20%) randomized to placebo and 5 of 50 participants (10%) randomized to usual care, with gains largely maintained through 1-year follow-up. Treatment effects on pain were mediated by reduced beliefs that pain indicates tissue damage, and longitudinal functional magnetic resonance imaging showed reduced prefrontal responses to evoked back pain and increased resting prefrontal-somatosensory connectivity in patients randomized to treatment relative to patients randomized to placebo or usual care.

**Meaning:**

Psychological treatment focused on changing beliefs about the causes and threat value of primary chronic back pain may provide substantial and durable pain relief.

## Teaser

This randomized clinical trial tests whether pain reprocessing therapy can provide durable and substantial relief from chronic back pain and investigates treatment mechanisms.

## Introduction

Chronic pain affects 20% of people in the US, with an estimated annual cost of more than $600 billion.^1,2^ The most common type is chronic back pain (CBP). In approximately 85% of cases, definitive peripheral causes of CBP cannot be identified, and central nervous system processes are thought to maintain pain.^3,4,5,6,7^ For people with this type of CBP— often referred to as primary, nonspecific, nociplastic, or centralized pain—psychological and behavioral treatments are recommended.^8,9,10^ Although these treatments can improve functioning, reductions in pain intensity are limited^11,12^ and better treatments are needed.

Advances in the neuroscience of pain^13,14,15,16,17^ and interoception^18,19,20,21^ suggest new directions for treatment development. In constructionist and active inference models, pain is a prediction about bodily harm, shaped by sensory input and context-based predictions.^18,19,22,23,24,25,26^ Fearful appraisals of tissue damage can cause innocuous somatosensory input to be interpreted and experienced as painful.^22,24,27,28^ Such constructed perceptions can become self-reinforcing: threat appraisals enhance pain, which is in turn threatening, creating positive feedback loops that maintain pain after initial injuries have healed.^27,29,30,31^

As pain becomes chronic, it is increasingly associated with activity in the affective and motivational systems tied to avoidance and less closely tied to systems encoding nociceptive input.^14,32,33,34^ Accordingly, brain regions serving allostasis and predictive control^18,23^—including the default mode network, somatosensory and insular cortices, amygdala, and nucleus accumbens—have been implicated in animal models^13,14,15,16,17^ and human studies of chronic pain^22,25,32,33,35,36^ and pain modulation.^24,25,28,37,38,39^

We developed pain reprocessing therapy (PRT) based on this understanding of primary chronic pain. Leading psychological interventions for pain typically present the causes of pain as multifaceted and aim primarily to improve functioning and secondarily to reduce pain. PRT emphasizes that the brain actively constructs primary chronic pain in the absence of tissue damage and that reappraising the causes and threat value of pain can reduce or eliminate it.

In this study, we conducted the first test of PRT. In a randomized clinical trial with 1-year follow-up, we compared PRT with both open-label placebo and usual care control conditions. We tested hypothesized mechanisms of PRT with mediation analyses and longitudinal functional magnetic resonance imaging (fMRI) during spontaneously occurring and evoked back pain. fMRI provided objective correlates of treatment effects and identified potential neurobiological treatment mechanisms.

## Methods

### Participants and Trial Design

The trial was preregistered on ClinicalTrials.gov (Identifier: NCT03294148) and conducted from August 2017 to November 2018, with 1-year follow-up completed by November 2019. Clinical and fMRI data were analyzed from January 2019 to August 2020, after data collection at each follow-up timepoint was complete. Participants aged 21 to 70 years with back pain for at least half the days of the last 6 months and 1-week average pain intensity score of 4 of 10 or greater at screening were recruited from the community in Boulder, Colorado. We targeted primary CBP, excluding patients with leg pain worse than back pain (eMethods in Supplement 2). Power analysis targeted 80% power (α = .05) to detect a medium effect (*d* = 0.62) on pain intensity at the primary end point (eMethods in Supplement 2). Participants provided written informed consent as approved by the University of Colorado Institutional Review Board. The study followed the Consolidated Standards of Reporting Trials (CONSORT) reporting guideline for social and psychological intervention trials.

**Figure 1.**
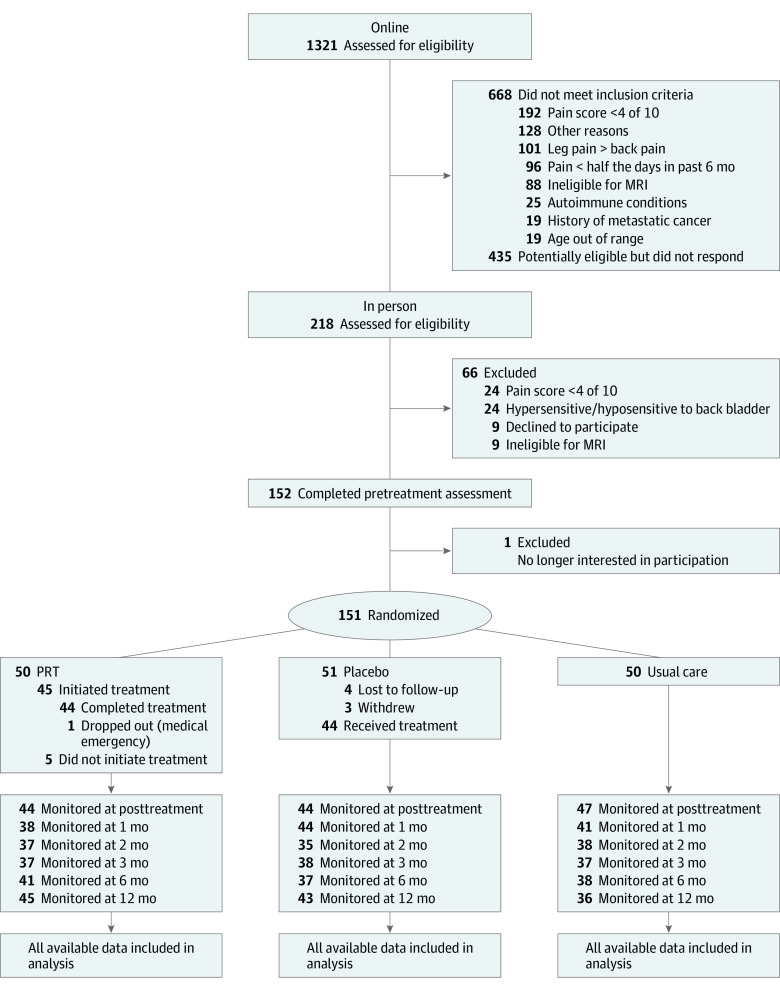
CONSORT Participant Flow Diagram MRI indicates magnetic resonance imaging.

Participants completed an eligibility and consent session, followed by a baseline assessment session with fMRI. They were subsequently randomized to PRT, placebo, or usual care with equal probability, balancing on age, sex, baseline pain, and opioid use using an imbalance-minimization algorithm^40^ (eMethods in Supplement 2). The primary end point (posttreatment fMRI session) occurred 1 month after the baseline fMRI. Participants completed online follow-up assessments at 1, 2, 3, 6, and 12 months after the primary end point (Figure 1).

### Interventions

#### PRT

PRT seeks to promote patients’ reconceptualization of primary (nociplastic) chronic pain as a brain-generated false alarm. PRT shares some concepts and techniques with existing treatments for pain^41,42,43,44,45,46,47,48^ and with the cognitive behavioral treatment of panic disorder.^66^

Participants completed a 1-hour telehealth evaluation and education session with a physician (H.S.) assessing likely centralized vs peripheral contributions to pain, including a review of available preexisting spinal imaging. Assessment findings and centralized pain education were shared with the patient (eAppendix 1 in Supplement 2).

Participants then completed 8 individual 1-hour therapy sessions with a therapist with extensive PRT experience (A.G. or C.U.) twice weekly for 4 weeks. Techniques included (1) providing personalized evidence for centralized pain; (2) guided reappraisal of pain sensations while seated and while engaging in feared postures or movements; (3) techniques addressing psychosocial threats (eg, difficult emotions) potentially amplifying pain; and (4) techniques to increase positive emotions and self-compassion. PRT followed the treatment protocol found in eAppendix 2 in Supplement 2.

Treatment fidelity was assessed by independent raters coding audiorecordings of PRT sessions (eMethods and eAppendix 3 in Supplement 2). A mean (SD) of 4.93 (0.87) of 6 PRT elements were present in each session, and all sessions included at least 3 elements, indicating high treatment fidelity.

#### Open-label Placebo Plus Usual Care

Participants watched 2 videos describing how placebo treatments can powerfully relieve pain even when known to be inert (eg, they can automatically trigger the body’s natural healing response).^49^ A subcutaneous injection described as saline was administered by a physician (K.K.) at the site of greatest back pain during an empathic, validating clinical encounter at an orthopedic medical center. Open-label placebo treatments are as effective or nearly as effective as traditional (deceptive) placebos for CBP and other chronic symptoms when administered in this manner (eMethods in Supplement 2).^50,51,52^ Participants in this group were also asked to continue their ongoing care as usual and not start other new treatments until after the study period.

#### Usual Care

Participants in this group were given no additional treatment. They agreed to continue their ongoing care as usual and not start new treatments before the posttreatment assessment. After the posttreatment assessment, they were given a chronic pain workbook^53^ and access to http://www.unlearnyourpain.com.

### Clinical Measures

The primary outcome was average pain over the last week on a numerical rating scale from 0 to 10 from the Brief Pain Inventory Short Form, assessed at the 1-month postbaseline session. We also calculated the proportion of participants reporting pain reduction of 30% or more, pain reduction of 50% or more, and a pain score of 0 or 1, indicating a pain-free or nearly pain-free state. Secondary outcomes included pain interference (Oswestry Disability Index); Patient-Reported Outcome Measurement Information System (PROMIS) short forms for depression, anxiety, anger, and sleep quality; and the Positive and Negative Affect Scale (measure details in the eMethods in Supplement 2).

We considered 3 measures of pain beliefs as potential mediators: (1) the Tampa Scale of Kinesiophobia (TSK-11), assessing belief that pain indicates injury and fear of movement; (2) the Pain Catastrophizing Scale (PCS); and (3) the Survey of Pain Attitudes Emotion subscale (SOPA-Emotion), assessing beliefs that stress and negative emotion increase pain. Adverse events were recorded when participants spontaneously reported them to study personnel. Baseline pain was computed as the average score from 2 prerandomization assessments (eligibility session and pretreatment fMRI session).

### Neuroimaging Measures

Structural T1 and multiband blood oxygenation level–dependent functional imaging was conducted on a 3-T Siemens Prisma Fit MRI scanner with standard fMRI preprocessing (eMethods in Supplement 2). During fMRI, participants completed (1) an evoked back pain task with a series of randomly ordered trials distending the back to 1 of 4 intensity levels and (2) a spontaneous pain scan in which participants rested and rated ongoing pain once per minute (design details in the eMethods in Supplement 2; fMRI data quality measures shown in eFigures 6 and 7 in Supplement 2). Participants rated pain during scanning on a visual analog scale from 0 (no pain) to 100 (worst pain imaginable).

### Statistical Analyses

Intent-to-treat analyses (including all randomized patients) were performed for the primary outcome with a mixed-effects model (*fitlme*, MATLAB 2020a), including 2 group × time interactions (PRT vs placebo × posttreatment vs pretreatment and PRT vs usual care × posttreatment vs pretreatment), covariates for age and sex, and a random intercept per participant. Treatment response rates for 30% or greater reduction in pain, 50% or greater reduction in pain, and a pain-free or nearly pain-free state at posttreatment and 1-year follow-up were based on all randomized patients; those missing data were considered nonresponders. For follow-up time points and secondary outcomes, we calculated Hedges *g* for the PRT vs placebo and PRT vs usual care comparisons. Follow-up time points were analyzed individually, testing group differences in change from baseline to each time points. The placebo vs usual care comparison will be reported elsewhere.

To investigate psychological treatment mechanisms, we (1) correlated pretreatment to posttreatment changes in pain intensity with pretreatment to posttreatment changes in pain beliefs (TSK-11, PCS, and SOPA-Emotion) within each group and (2) tested pretreatment to posttreatment changes in pain beliefs as mediators of treatment effects on pain at follow-up timepoints (1 through 12 months posttreatment), controlling for baseline pain. PRT vs placebo and PRT vs usual care were tested in separate models. We also tested the reverse: whether pretreatment to posttreatment pain reductions mediated treatment effects on pain beliefs at follow-up, controlling for baseline pain beliefs (eMethods in Supplement 2). Correlational and mediation analyses were not prespecified in the trial protocol.

#### Evoked Back Pain Analyses

An evoked back pain localizer identified brain regions positively associated with evoked back pain intensity at baseline. The localizer was conducted within a mask of regions of interest (medial prefrontal, posteromedial, insula, cingulate, and somatosensory cortices; amygdala; and nucleus accumbens; eMethods and eFigure 1 in Supplement 2; localizer task design in eFigure 8 in Supplement 2). We tested for treatment effects (group × time interactions) in the average activity of clusters positively associated with evoked back pain using a mixed-effects (random-effects) model, applying a 1-tailed threshold of *P* < .05 owing to directional hypotheses that PRT would reduce activity in pain-positive clusters.

#### Spontaneous Pain Connectivity Analyses

Evoked pain analyses identified group × time interactions in the anterior insula, anterior midcingulate (aMCC), and a prefrontal region. We submitted these 3 regions as seeds to connectivity analyses in the spontaneous pain scan. We conducted permutation tests (threshold-free cluster-enhancement; eMethods in Supplement 2) testing for group × time interactions in connectivity between these seed regions and 2 areas most often demonstrating altered connectivity in chronic pain: (1) the midline default mode network, including the medial prefrontal and posteromedial cortex, and (2) primary somatosensory cortex (S1)^36,54,55,56,57,58,59^ (masks in eFigure 2 in Supplement 2).

## Results

We randomized 151 participants (54% female; mean [SD] age, 41.1 [15.6] years; mean [SD] CBP duration, 10.0 [8.9] years). At baseline, patients reported low to moderate pain intensity scores (mean [SD], 4.10 [1.26]) to 4.41 [1.29]) and disability (mean [SD], 23.34 [10.12] on the Oswestry Disability Index), with similar pain and demographic characteristics across groups (Table 1).

**Table 1.** Baseline Patient Characteristics

Characteristic	No. (%)		
	Pain reprocessing therapy	Placebo	Usual care
**Demographic characteristics**			
Age, mean (SD), y	42.6 (16.2)	39.4 (14.9)	41.3 (15.9)
Sex			
Female	29 (58)	25 (49)	27 (54)
Male	21 (42)	26 (51)	23 (46)
Education			
High school or less	0	0	0
Some college	11 (22)	15 (29)	15 (30)
College graduate	39 (78)	36 (71)	35 (70)
Married	26 (52)	25 (49)	30 (60)
Race^a^			
American Indian or Alaskan Native	0	0	1 (2)
Asian/Pacific Islander	3 (6)	2 (4)	0
Black (not of Hispanic origin)	0	2 (4)	1 (2)
White (not of Hispanic origin)	46 (92)	45 (88)	43 (86)
Other or unknown	1 (2)	2 (4)	5 (10)
Hispanic ethnicity	0	2 (4)	2 (4)
Employment status			
Full-time (>30 h/wk)	33 (66)	26 (51)	28 (56)
Part-time (5-30 h/wk)	10 (20)	12 (24)	13 (26)
Unemployed/lightly employed (<5 h/wk)	7 (14)	13 (25)	9 (18)
Subjective socioeconomic status, mean (SD), 1-10^60^	6.8 (1.8)	6.4 (2.0)	6.7 (1.6)
Exercise			
Almost none	6 (12)	1 (2)	4 (8)
1 h/wk	4 (8)	7 (14)	9 (18)
3 h/wk	17 (34)	23 (45)	14 (28)
7 h/wk	19 (38)	18 (35)	21 (42)
≥14 h/wk	4 (8)	2 (4)	2 (4)
**Pain-related characteristics**			
Pain duration, mean (SD), y	10.7 (9.7)	8.9 (8.2)	10.5 (8.9)
Current opioid use (yes/no)	5 (10)	2 (4)	2 (4)
Pain in body sites besides back?			
None	5 (10)	9 (18)	4 (8)
A little	29 (58)	24 (47)	28 (56)
A moderate amount	11 (22)	15 (29)	16 (32)
A lot	5 (10)	3 (6)	2 (4)

^a^ Race and ethnicity were collected in accord with National Institutes of Health guidelines by multiple choice self-report.

Of 50 participants randomized to PRT, 44 (88%) completed all treatment sessions and the posttreatment assessment. Five participants dropped out prior to initiating PRT and 1 had an unrelated medical emergency. Of 51 participants randomized to placebo, 44 (86%) received the treatment, all of whom completed the posttreatment assessment. Of the 50 participants randomized to usual care, 47 (94%) completed the posttreatment assessment (Figure 1).

Twenty patients in the PRT group had preexisting spinal imaging, all of which showed at least 1 spinal anomaly (median of 4 findings per patient; eTable 1 in Supplement 2) assessed by a physician (H.S.) as not causal of pain (eMethods and eAppendix 1 in Supplement 2).^61^

### Clinical Outcomes

**Figure 2.**
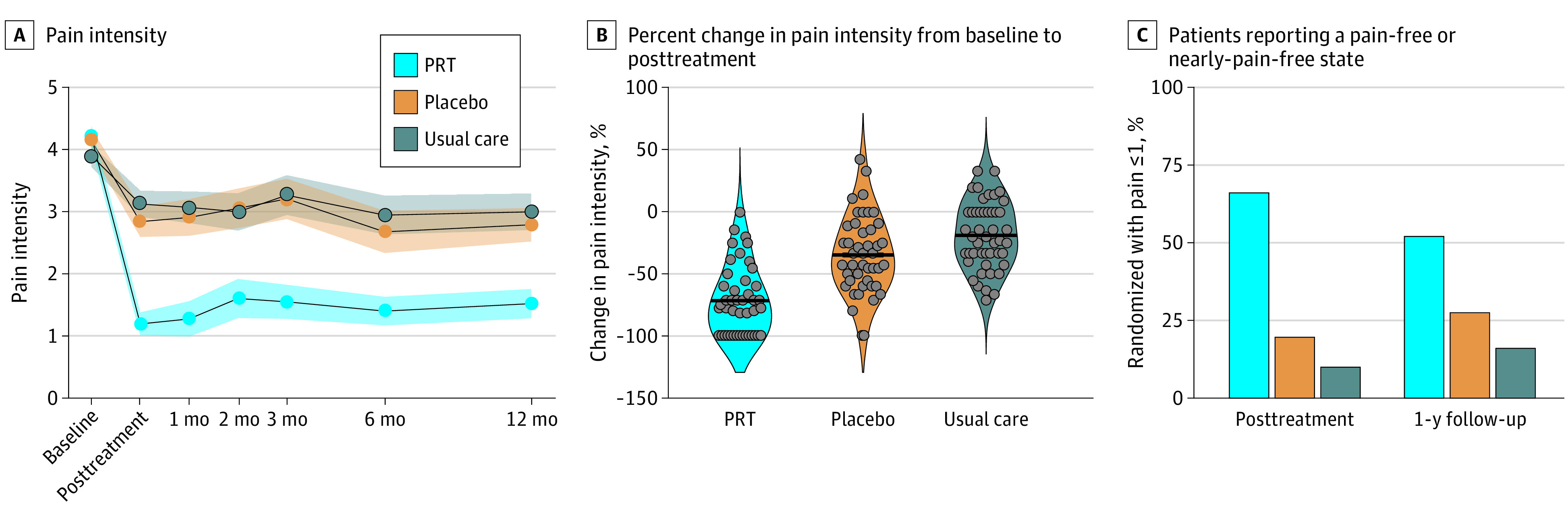
Clinical Outcomes A, Shading indicates standard error. B, Dots represent individual participants; thick lines represent the group mean. C, Percentage of patients reporting pain scores of 0 or 1 of 10 (ie, pain-free or nearly pain-free) at posttreatment and at 1-year follow-up. PRT indicates pain reprocessing therapy.

Patients randomized to PRT reported substantial reductions in pain intensity at posttreatment compared with both control groups, with a mean (SD) pain score of 1.18 (1.24) in the PRT group, 2.84 (1.64) in the placebo group, and 3.13 (1.45) in the usual care group (Figure 2; Table 2). Patients in the PRT group reported a pain reduction of 1.79 (on the 0 to 10 numerical rating scale) relative to placebo (*t*_137.63_ = 6.06; *P* < *.*001; *g*, −1.14; 95% CI, −1.65 to −0.71) and reported a pain reduction of 2.40 relative to the usual care group (*t*_135.69_ = 8.13; *P* < *.*001; *g*, −1.74; 95% CI, −2.28 to −1.32). A total of 33 of 50 patients randomized to PRT (66%), corresponding to 73% of the 45 patients who initiated PRT, were pain-free or nearly pain-free at posttreatment, compared with 10 of 51 patients (20%) in the placebo group and 5 of 50 patients (10%) in the usual care group. At 1-year follow-up, effects of PRT on pain remained large relative to both control groups, with a mean (SD) pain score of 1.51 (1.59) in the PRT group, 2.79 (1.78) in the placebo group, and 3.00 (1.77) in the usual care group. Hedges *g* was −0.70 for PRT vs placebo (*P* = .001) and −1.05 for PRT vs usual care (*P* < .001) (Table 2; treatment response rates in eTable 2 in Supplement 2; individual patient pain trajectories in eFigure 3 in Supplement 2).

**Table 2.** Primary and Secondary Clinical Outcomes

Between-group differences^a^	Mean (SD)			PRT vs placebo, *g* (SE)^b^	*P* value	PRT vs usual care, *g* (SE)^b^	*P* value
	PRT	Placebo	Usual care				
**Primary outcome**							
Pain intensity (0-10)							
Baseline	4.22 (1.21)	4.16 (1.33)	3.91 (1.24)	NA	NA	NA	NA
Posttreatment	1.18 (1.24)	2.84 (1.64)	3.13 (1.45)	−1.14 (0.24)	<.001	−1.75 (0.24)	<.001
At 1 mo	1.26 (1.77)	2.91 (1.97)	3.07 (1.63)	−0.83 (0.27)	<.001	−1.24 (0.29)	<.001
At 2 mo	1.59 (1.92)	3.06 (1.89)	3.00 (1.86)	−0.84 (0.28)	.001	−1.03 (0.28)	<.001
At 3 mo	1.54 (1.68)	3.21 (2.02)	3.27 (1.95)	−0.93 (0.23)	<.001	−1.35 (0.25)	<.001
At 6 mo	1.39 (1.48)	2.68 (2.08)	2.95 (1.93)	−0.74 (0.23)	.001	−1.14 (0.26)	<.001
At 12 mo	1.51 (1.59)	2.79 (1.78)	3.00 (1.77)	−0.70 (0.21)	.001	−1.05 (0.24)	<.001
**Secondary outcome**							
Oswestry Disability Index (0-100)							
Baseline	23.70 (10.70)	23.06 (10.14)	23.26 (9.67)	NA	NA	NA	NA
Posttreatment	10.14 (10.63)	19.00 (11.07)	20.68 (10.68)	−1.30 (0.28)	<.001	−1.70 (0.26)	<.001
At 1 mo	10.58 (14.26)	18.68 (11.95)	20.30 (9.04)	−1.04 (0.25)	<.001	−1.61 (0.27)	<.001
At 2 mo	9.57 (12.86)	19.43 (11.84)	21.37 (11.07)	−1.30 (0.29)	<.001	−1.55 (0.23)	<.001
At 3 mo	9.68 (13.39)	21.42 (14.32)	23.57 (13.36)	−1.26 (0.28)	<.001	−1.61 (0.25)	<.001
At 6 mo	9.80 (11.94)	18.50 (13.43)	20.84 (11.57)	−0.96 (0.26)	<.001	−1.3 (0.28)	<.001
At 12 mo	11.16 (13.13)	18.52 (12.60)	18.78 (12.59)	−0.23	<.001	−0.83 (0.24)	<.001
PROMIS depression, raw score (8-32)							
Baseline	14.66 (4.39)	13.17 (4.67)	12.85 (4.74)	NA	NA	NA	NA
Posttreatment	12.23 (4.94)	11.75 (4.05)	11.81 (4.45)	−0.35 (0.24)	.099	−0.56 (0.24)	.009
At 1 mo	12.87 (5.23)	10.64 (3.57)	11.57 (4.61)	0.13 (0.23)	.555	−0.54 (0.25)	.019
At 2 mo	12.51 (4.88)	11.11 (4.95)	11.76 (5.17)	−0.08 (0.24)	.723	−0.51 (0.24)	.028
At 3 mo	11.47 (4.64)	12.45 (6.09)	12.30 (4.51)	−0.57 (0.24)	.015	−0.90 (0.22)	<.001
At 6 mo	12.90 (5.28)	10.97 (4.00)	11.84 (4.65)	−0.09 (0.24)	.701	−0.47 (0.23)	0.40
At 12 mo	12.53 (5.12)	11.95 (5.86)	12.75 (4.50)	−0.20 (0.23)	.360	−0.62 (0.24)	.007
PROMIS anger, raw score (5-25)							
Baseline	12.46 (3.73)	10.97 (3.18)	11.17 (3.18)	NA	NA	NA	NA
Posttreatment	9.52 (3.91)	9.89 (3.81)	10.45 (3.86)	−0.62 (0.21)	.004	−0.78 (0.21)	<.001
At 1 mo	9.50 (4.40)	8.84 (3.27)	10.55 (3.19)	−0.23 (0.25)	.291	−0.91 (0.25)	<.001
At 2 mo	10.70 (4.68)	9.37 (3.30)	10.00 (3.92)	−0.11 (0.23)	.652	−0.28 (0.25)	.231
At 3 mo	9.31 (4.06)	9.87 (4.78)	10.49 (3.52)	−0.52 (0.21)	.027	−0.92 (0.25)	<.001
At 6 mo	9.83 (4.49)	9.31 (2.96)	10.51 (3.44)	−0.38 (0.25)	.099	−0.90 (0.23)	<.001
At 12 mo	10.49 (4.15)	9.64 (3.55)	10.89 (3.38)	−0.16 (0.21)	.454	−0.61 (0.22)	.008
PROMIS anxiety, raw scores (8-40)							
Baseline	16.37 (5.88)	15.52 (5.83)	15.11 (6.40)	NA	NA	NA	NA
Posttreatment	15.02 (6.16)	13.89 (5.78)	14.11 (6.99)	0 (0.22)	1.00	−0.21 (0.21)	.318
At 1 mo	14.58 (6.45)	12.25 (4.81)	13.75 (6.78)	0.36 (0.20)	.109	−0.29 (0.21)	.203
At 2 mo	14.14 (7.07)	13.23 (6.74)	13.58 (6.75)	0.02 (0.24)	.923	−0.22 (0.25)	.348
At 3 mo	13.75 (6.45)	14.50 (7.42)	14.08 (6.42)	−0.34 (0.23)	.147	−0.62 (0.21)	.009
At 6 mo	14.88 (7.12)	13.00 (5.14)	14.59 (6.90)	0.03 (0.24)	.907	−0.50 (0.24)	.028
At 12 mo	14.09 (6.79)	14.07 (7.51)	14.81 (6.94)	−0.20 (0.22)	.362	−0.56 (0.23)	.014
PROMIS sleep, raw score (8-40)							
Baseline	22.21 (6.54)	22.65 (6.38)	22.63 (6.26)	NA	NA	NA	NA
Posttreatment	17.73 (6.75)	20.50 (6.17)	20.89 (6.02)	−0.41 (0.23)	.056	−0.63 (0.22)	.003
At 1 mo	17.18 (6.38)	21.02 (6.34)	21.62 (6.45)	−0.46 (0.25)	.039	−0.89 (0.27)	<.001
At 2 mo	17.08 (6.71)	19.71 (6.72)	21.74 (7.19)	−0.38 (0.24)	.112	−0.84 (0.27)	<.001
At 3 mo	16.67 (6.67)	20.16 (7.05)	21.73 (6.26)	−0.44 (0.24)	.061	−1.08 (0.24)	<.001
At 6 mo	17.85 (7.24)	19.42 (6.22)	21.38 (6.03)	−0.29 (0.23)	.198	−0.85 (0.23)	<.001
At 12 mo	18.11 (7.36)	19.95 (5.79)	21.19 (6.73)	−0.23 (0.22)	.272	−0.60 (0.25)	.009

Abbreviations: NA, not applicable; PROMIS, Patient-Reported Outcome Measurement Information System; PRT, pain reprocessing therapy.

^a^ Effect sizes show the group difference in change from baseline (group × time interaction), including all available data at the follow-up time point and corresponding baseline data for effect size computation.

^b^ Hedges *g* and SE estimated with bootstrapping procedure.

Analyses of secondary outcomes at posttreatment revealed significant reductions in disability and anger for PRT vs both controls (*g*, −0.62 to −1.7; *P* < .005) and improvements in sleep (*g*, −0.56; *P* = .009) and depression (*g*, −0.63; *P* = .003) relative to usual care (Table 2). Treatment gains on secondary outcomes were largely maintained at 1-year follow-up (Table 2). Significant PRT vs control effects were observed at posttreatment for positive affect (Positive and Negative Affect Schedule; *g* for PRT vs placebo, 0.63, *g* for PRT vs usual care, 0.59; *P* < .005; eTable 3 in Supplement 2) but not for negative affect or alcohol, cannabis, or opioid use (eTable 3 in Supplement 2). Treatment satisfaction was high among participants in the PRT group (eTable 4 in Supplement 2).

### Mediation Analyses

Pretreatment to posttreatment reductions in TSK-11 and pain intensity scores were correlated among participants in the PRT group (*r*_42_ = 0.44; *P* = .003; eFigure 4 in Supplement 2). This correlation was not significant for the placebo condition (*r*_42_ = 0.16; *P* = .29) or usual care condition (*r*_45_ = 0.27; *P* = .07). Pretreatment to posttreatment changes in PCS and SOPA-Emotion scores did not correlate with pain reductions within any group.

Pretreatment to posttreatment reductions in TSK-11 scores mediated PRT vs placebo and PRT vs usual care effects on pain intensity at most follow-up time points (eFigure 4 and eTables 5 and 6 in Supplement 2). The reverse was also true: pretreatment to posttreatment pain reductions mediated PRT vs placebo and PRT vs usual care effects on TSK-11 at follow-up. Pretreatment to posttreatment changes in PCS and SOPA-Emotion did not mediate PRT vs control effects at any follow-up time point. Treatment effects on TSK-11 were very large at posttreatment (*g* for PRT vs placebo, −1.90; *g* for PRT vs usual care,−1.67; *P* < .001).

Neither age nor sex moderated the treatment effect on pain intensity (eMethods in Supplement 2). No adverse events were reported for PRT.

### Neuroimaging Outcomes

#### Evoked Back Pain

At baseline, increased back distention led to increased pain (mean [SD] for distention level 1, 32.15 [18.57]; distention level 2, 37.91 [20.30]; distention level 3, 46.70 [21.71]; distention level 4, 52.73 [21.78]). There was a significant effect of distention level on pain (mean [SD] β for inflation, 7.05 [5.06]; *t*_95_ = 13.64; *P* < .001. Individual patient-evoked pain data are shown in eFigure 5 in Supplement 2.

**Figure 3.**
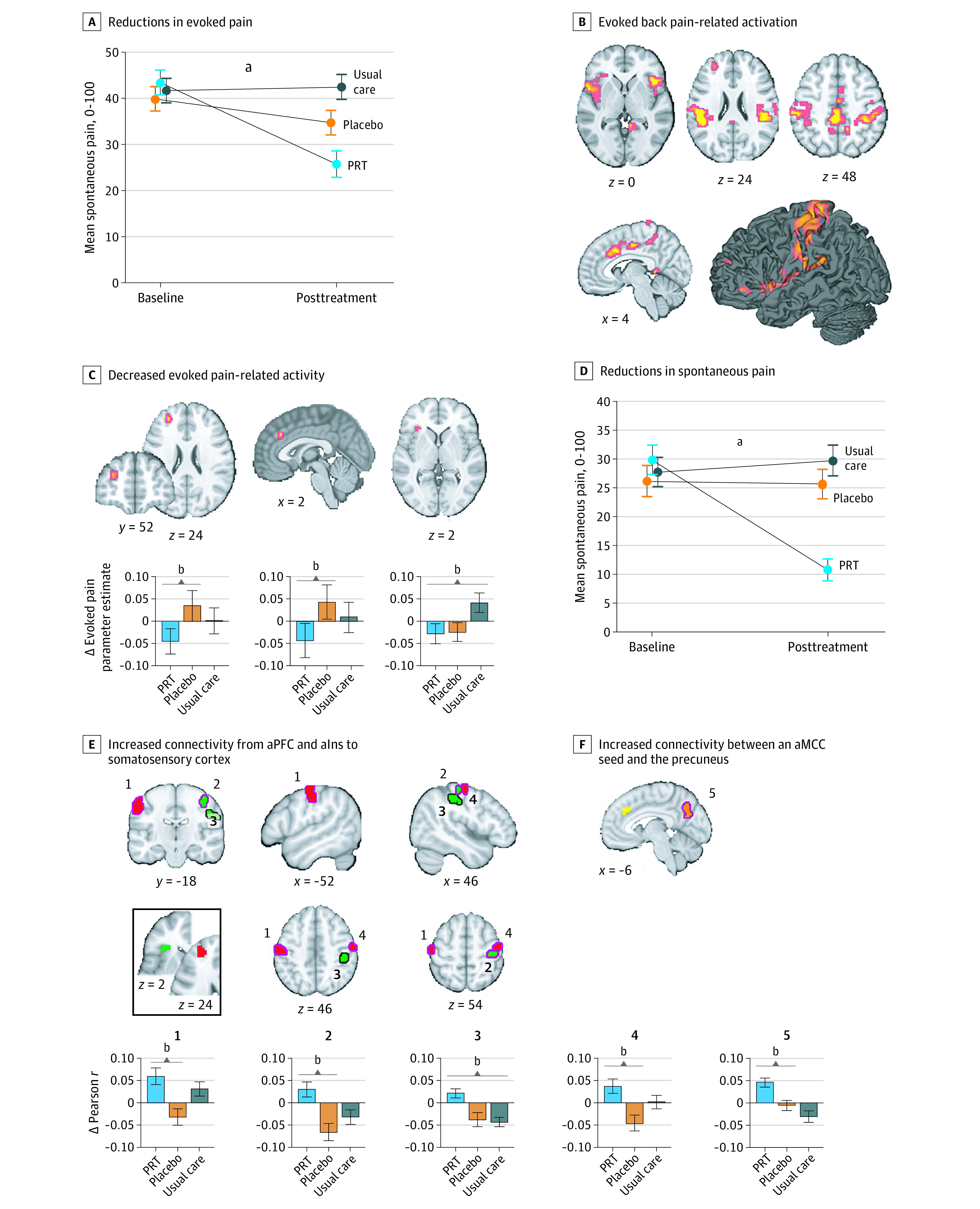
Effects of Treatment on Evoked and Spontaneous Back Pain and Related Brain Function A, Error bars show standard error. B, Coordinates and statistics for activations provided in eTable 7 in Supplement 2; analyses conducted within a mask of regions of interest; eFigure 1 in Supplement 2. C, Decreased evoked pain-related activity was observed in anterior midcingulate (aMCC) and anterior prefrontal regions for PRT vs placebo and left anterior insula for PRT vs usual care. D, Error bars show standard error. E, PRT vs control conditions increased aPFC-seeded (red clusters) and aIns-seeded (green clusters) connectivity with primary somatosensory cortex (permutation test, *P* < .05). Inset shows seed regions, derived from evoked pain analyses; magenta outlines, PRT vs placebo; black outlines, PRT vs usual care. F, PRT vs usual care increased connectivity between an aMCC seed (yellow; derived from evoked back pain analyses) and the precuneus (orange). Connectivity analyses were conducted within primary somatosensory cortex and medial default mode network masks. ^a^*P* < .001. ^b^*P* < .05.

Patients receiving PRT reported significant pretreatment to posttreatment reductions in evoked back pain relative to placebo (β, −13.05 on a 101-point visual analog scale; *t*_122.85_ = −2.82; *P* = .006; *g*, −0.60; 95% CI, −1.06 to −0.16) and relative to usual care (β, −19.61; *t*_79.52_ = −4.03; *P* < .001; *g*, −0.99; 95% CI, −1.50 to −0.55; Figure 3A). Among patients in the PRT group, pretreatment to posttreatment reductions in evoked back pain and 1-week average back pain intensity were correlated (*r*_32_ = 0.47; *P* = .005).

Localizer analyses identified 16 regions within the mask of interest positively associated with evoked pain intensity, including bilateral insula, cingulate, bilateral somatotopic back areas S1 and secondary somatosensory cortex, and prefrontal regions (Figure 3B; eFigure 1 and eTable 7 in Supplement 2). Relative to placebo, PRT reduced pain-related activity in aMCC (*t*_133.48_ = −1.73; *P* = .04) and the anterior prefrontal cortex (aPFC; *t*_133.48_ = −1.85; *P* = .03). Relative to usual care, PRT reduced pain-related activity in the left anterior insula (aIns; *t*_120.1_ = −2.34; *P* = .01; Figure 3C).

#### Spontaneous Pain

Patients receiving PRT reported reductions in spontaneous pain relative to placebo (β, −18.24 on a 101-point visual analog scale; *t*_140.66_ = −4.59; *P* < .001; *g*, −0.92; 95% CI, −1.44 to −0.47) and relative to usual care (β, −21.53; *t*_79_ = −5.26; *P* < .001; *g*, −1.11; 95% CI, −1.66 to −0.66; Figure 3D).

We submitted the aMCC, aPFC, and aIns regions exhibiting treatment effects in evoked pain analyses as connectivity seed regions in the spontaneous pain task. Within S1, PRT vs placebo and PRT vs usual care led to increased aPFC- and aIns-seeded connectivity to 4 distinct S1 subregions (permutation test COPE-MAX, 3.55-3.91; *P* < .05). Within the medial default mode network, PRT vs usual care increased aMCC-precuneus connectivity (permutation test COPE-MAX, 4.23; *P* = .01; Figure 3E; cluster coordinates and statistics in eTable 8 in Supplement 2). No group × time interactions were found for aPFC- or aIns-seeded connectivity to default mode network regions or for aMCC-seeded connectivity to S1.

## Discussion

PRT yielded large reductions in CBP intensity relative to open-label placebo and usual care control conditions in a community sample, with nearly two-thirds of randomized patients and 73% of those initiating PRT reporting they were pain-free or nearly pain-free at posttreatment. Large effects of PRT on pain continued at 1-year follow-up. PRT also reduced experimentally evoked back pain and spontaneous pain during fMRI with large effect sizes, and several secondary outcomes (eg, disability and anger) also improved for PRT relative to the control groups.

PRT targets primary (nociplastic) pain by shifting patients’ beliefs about the causes and threat value of pain. It presents pain as a reversible, brain-generated phenomenon not indicative of peripheral pathology, consistent with active inference and constructionist accounts of interoception and pain.^18,19,22,23,24,25,26,27^ PRT builds on and extends existing psychological treatment models. Cognitive-behavioral, acceptance-based, and mindfulness-based interventions typically aim to improve functioning by decreasing pain catastrophizing, enhancing pain coping or acceptance, and promoting engagement in valued life activities.^41,44,46,48,62^ Exposure-based treatments share with PRT an emphasis that painful activities are not injurious,^42,63,64,65^ but do not emphasize reappraising pain sensations and reattributing the causes of pain. Some pain neuroscience education interventions present pain in a similar way as PRT,^43^ though they typically lack guided exposure and reappraisal exercises.

Large reductions in pain are rarely observed in CBP psychological treatment trials.^11,12^ Relatively unique components of PRT potentially contributing to the observed effects include (1) an in-depth medical and psychological assessment generating personalized evidence for centralized pain; (2) reattribution of pain to reversible learning- and affect-related brain processes rather than bodily injury; and (3) a unique combination of cognitive, somatic, and exposure-based techniques supporting pain reappraisal (eDiscussion in Supplement 2).

Correlational and mediational analysis results support changes in fear-inducing pain beliefs as a potential PRT mechanism. Effects of PRT on pain beliefs were also mediated by pain intensity reductions, perhaps because pain reductions promote beliefs in pain modifiability (eDiscussion in Supplement 2). Changes in pain beliefs are not unique to PRT, but PRT may more strongly change these beliefs compared with existing therapies (eTable 6 in Supplement 2).

These hypothesized mechanisms are consistent with extinction-based treatment approaches to anxiety disorders.^42,65^ For example, 85% of patients became free of panic symptoms following treatment focused on reappraising somatic symptoms as caused by nondangerous central nervous system processes (eg, false alarms).^66^

PRT reduced evoked pain-related activity in aPFC, aMCC, and aIns. The aPFC and adjacent dorsolateral prefrontal cortex (dlPFC) are implicated in the detection and inhibition of pain.^67^ aPFC reductions following PRT suggest a potential reduction of pain-related signals or decreased prioritization of pain control. The aMCC and aIns are cortical convergence zones in the construction of negative affect in pain and other domains.^20,68,69,70^ Cognitive pain regulation strategies, including mindful acceptance^38,39^ and placebo analgesia,^24,25,28^ have been found to reduce aMCC and aIns responses to pain, demonstrating parallels between experimental findings and our clinical findings. The aIns reductions in our study were not specific to PRT vs placebo and may reflect processes common to both these interventions.

PRT also increased aPFC and aIns connectivity to S1, aligning with previous findings that cognitive behavioral therapy for fibromyalgia^57^ and acupuncture for CBP^55^ increased aIns-S1 connectivity. Increased aPFC and aIns connectivity to S1 may reflect increased attention to somatosensory input in constructing pain.^71^ This is congruent with mindfulness-based treatments promoting nonreactive attention to bodily sensations, reducing catastrophizing.^38,39,48,71^ Yet, increased S1 connectivity has also been associated with increased clinical pain,^72^ and the role of S1 connectivity remains unclear.^55^ PRT vs usual care also increased aMCC-precuneus connectivity, with intermediate effects observed in participants receiving placebo treatment. Altered default mode connectivity has often been reported in chronic pain, although typically with heightened connectivity for patients vs controls (eDiscussion in Supplement 2).^36,54,56,58,59^

### Limitations

This study has limitations. The study sample was relatively well educated and active and reported long-standing low to moderate pain and disability at baseline. The physician and therapists were experts in the treatment model. Future studies should test generalizability to other patient populations, therapists, and treatment contexts (eDiscussion in Supplement 2). The fMRI effect sizes were modest, with some results not surviving whole-brain correction (eMethods in Supplement 2). Future trials should test PRT efficacy relative to leading psychological and medical treatments (eDiscussion in Supplement 2).

## Conclusions

Overall, our findings raise key possibilities about the nature and treatment of primary CBP. Changing fear- and avoidance-inducing beliefs about the causes and threat value of pain may provide substantial, durable pain relief for people with primary CBP.

## Notes

Supplement 1. Trial protocol

Supplement 2. eMethods eResults eDiscussion eTable 1. Spinal anomalies among participants randomized to PRT eTable 2. Treatment response rates eTable 3. Secondary clinical outcomes measured only at pretreatment and posttreatment eTable 4. Treatment satisfaction and patient global impression of change eTable 5. Mediation results eTable 6. Values for mediators at each timepoint eTable 7. Evoked back pain localizer results eTable 8. Regions showing pretreatment to posttreatment connectivity changes for PRT vs placebo or PRT vs usual care eFigure 1. Evoked back pain localizer eFigure 2. Target masks for seed connectivity analyses eFigure 3. Individual trajectories of pain intensity for participants in the PRT, placebo and usual care groups eFigure 4. Effects of PRT on pain-related fear and avoidance and beliefs that pain indicates injury eFigure 5. Evoked back pain at pretreatment eFigure 6. High vs low thumb pressure stimulation eFigure 7. Histogram of quality control-functional connectivity correlations for spontaneous pain scans eFigure 8. Continuous pain regressors for 4 randomly chosen sample individuals eAppendix 1. Initial medical pain assessment and education session eAppendix 2. Pain reprocessing therapy description eAppendix 3. PRT treatment fidelity checklist eReferences

Supplement 3. Data sharing statement.
