# Supplementary material for: Effect of Pain Reprocessing Therapy vs Placebo and Usual Care for Patients With Chronic Back Pain: A Randomized Clinical Trial
Source: JAMA Psychiatry. 2021 Sep 29;79(1):1–12. doi: 10.1001/jamapsychiatry.2021.2669 (PMC8482298; doi:10.1001/jamapsychiatry.2021.2669)
Supplement: Supplement 1. — Trial protocol [file jamapsychiatry-e212669-s001.pdf]

University of Colorado Boulder

TITLE: Mind-body treatments for chronic back pain

PROTOCOL VERSION DATE: September 5, 2018
VERSION: 4.4

NCT03294148

PRINCIPAL INVESTIGATOR (PI):

Name: Tor Wager
Address: 345 UCB
Telephone: (303)895-8739
Email: tor.wager@colorado.edu

KEY PERSONNEL

Name: Tor Wager
Role in project: PI

Name: Jonathan Ashar
Role in project: Co-investigator with edit

Name: Sona Dimidjian
Role in project: Co-investigator

Name: Daniel Ott
Role in project: Co-investigator

Name: Thomas Flood
Role in project: Co-investigator

Name: Howard Schubiner
Role in project: Co-investigator

Name: Alan Gordon
Role in project: Co-investigator

Name: Daniel Lyman
Role in project: Co-investigator

Name: Judith Carlisle
Role in project: Co-investigator

Name: Joseph Clark
Role in project: Co-investigator

Name: David Messinger
Role in project: Co-investigator

Name: Christie Uipi
Role in project: Co-investigator

Name: Ed Walton
Role in project: Co-investigator

Name: Karen Knight
Role in project: Co-investigator

Name: Zachary Anderson
Role in project: Co-investigator

Name: Laurie Polisky
Role in project: Co-investigator

Name: Jordan Griffin
Role in project: Research coordinator

Name: Daniel Kusko
Role in project: Research assistant

Name: Victoria Schelkun
Role in project: Research assistant

I. OBJECTIVES

Hypotheses:

1) Non-deceptive, transparently administered placebo treatment can provide clinically meaningful relief of chronic back pain (CBP)
2) Psychotherapy including a novel psychoeducational component can provide clinically meaningful relief of chronic back pain (CBP)
3) Brain systems supporting learning about pain will be altered in CBP patients

Specific Aims:

1) To test the efficacy of a safe, non-deceptive, transparently administered placebo treatment for chronic back pain (CBP) relative to waitlist. Outcomes include self-report measures of pain and functioning, and measures of brain and immune function.

2) To test the efficacy of psychotherapy including a novel psychoeducational component for chronic back pain (CBP) relative to waitlist. Outcomes include self-report measures of pain and functioning, and measures of brain and immune function.
3) To characterize brain systems supporting learning about pain in CBP patients

II. BACKGROUND AND SIGNIFICANCE

Placebo treatments provide a window into the therapeutic encounter. Because they are pharmacologically inert, the effects of placebo treatments on the body are mediated by the patient's mind and brain. Placebo effects are caused by the treatment context, including the supportive care provided during the therapeutic encounter (Kaptchuk, 2002; F. G. Miller & Kaptchuk, 2008; Wager & Atlas, 2015), and can be estimated by comparing placebo and no-treatment control groups. Recent studies (Kam-Hansen et al., 2014; Kaptchuk et al., 2010; Kelley, Kaptchuk, Cusin, Lipkin, & Fava, 2012), including one from our group (Schafer, Colloca, & Wager, 2015), suggest that a transparently prescribed "open-label" placebo can provide effective pain relief—even though participants know they are receiving an inert treatment. Importantly, the treatment is non-deceptive, and thus can ethically be administered to patients in clinical settings (Blease, Colloca, & Kaptchuk, 2016). Patients believe that open-label placebo treatments are ethical and can be therapeutic (Hull et al., 2013; Ortiz, Chandros Hull, & Colloca, 2016; Tilburt, Emanuel, Kaptchuk, Curlin, & Miller, 2008). By comparing open-label placebo treatment with a no-treatment control group, one can assess the effects of the therapeutic encounter independent of beliefs in the specific treatment itself. This is the goal of the present study.

Chronic low back pain is, in many respects, an ideal domain for investigating clinical effects of the therapeutic encounter. It is debilitating and highly prevalent: Low back pain is a leading cause of disability in the industrialized world, with lifetime prevalence estimates ranging from 50 to 80% (Cohen, Argoff, & Carragee, 2008; Freburger et al., 2009). Thus, both the availability of patients and the impact of placebo studies on back pain are high. Back pain is also influenced by placebo treatments such as sham acupuncture or placebo pills in previous clinical studies (Haake et al., 2007; Hashmi, Baria, et al., 2012; Hashmi, Baliki, et al., 2012; Tuttle et al., 2015). However, only one study to our knowledge has compared placebo to a no treatment condition (Müller et al., 2016), which is necessary to differentiate placebo effects from other factors, like regression to the mean. Further, open-label placebo treatment is a new frontier.

Among treatments for back pain, steroid injection for pain is a medical procedure well suited to placebo studies. Steroid injection is the single most commonly performed intervention for back pain in the United States, with more than 2 million being performed in 2008 for Medicare patients alone (Manchikanti et al., 2012). Our collaborator at the Panorama Orthopedics and Spine Center, Dr. Karen Knight, performs several dozens such injections each month. But surprisingly, despite its prevalence, a recent meta-analysis concluded that steroid injection is no more effective than placebo injections (Bicket, Gupta, Brown, & Cohen, 2013). The beneficial results that have led to its widespread use are likely due to the therapeutic encounter and injection procedure itself; the steroid is superfluous. For these reasons, we have chosen a placebo injection procedure (subcutaneous saline injection into the lower back) for the current study.

Additionally, cognitive behavioral psychotherapies have established efficacy for CBP, with meta-analyses estimating small to medium sized effects relative to no treatment (Cherkin et al., 2016; Hoffman, Papas,

Chatkoff, & Kerns, 2007). Here, we test a novel psychotherapy that builds off existing psychotherapeutic approaches. Most patients with CBP have no pathology in the back that can be identified as the cause of pain (Chou, 2007; Deyo et al., 2014). In other words, medical examination reveals that the back appears fundamentally healthy and intact, rather than damaged. Here, we will test a psychotherapy intervention that helps patients consider whether their back is healthy or damaged, and suggests that if the back is healthy, the pain is a false alarm that does not connote harm.

In this study, we will provide the first answers to questions that could influence medical training and practice. Are open-label subcutaneous placebo injections into the back effective in relieving pain, and do they produce objective, physiological evidence of pain relief? Such physiological evidence is needed, as self-reported pain is subject to several biases, including demand characteristics (patient compliance with perceived research objectives), which may be especially prominent in open-label placebo trials. Additionally, we will test a novel psychotherapy that has the potential to significantly advance the treatment of CBP.

Physiological measures of pain relief will be provided by brain MRI and measures of immune function. In CBP, structural and functional MRI of the brain have documented reliable changes in resting state activity, responses to experimentally induced pain, and gray matter density relative to healthy controls (Kregel et al., 2015). Further, functional and structural MRI measures can normalize after successful treatment for back pain (Seminowicz et al., 2011; Shpaner et al., 2014). These measures have also been shown to track the natural course of recovery vs. chronification of acute back pain (Baliki et al., 2012; Hashmi et al., 2013; Vachon-Presseau et al., 2016). The immune system is also a central contributor to chronic pain (Grace, Hutchinson, Maier, & Watkins, 2014). Two previous studies have reported that peripheral (blood) measures of inflammation reflect the severity of CBP (Pedersen, Schistad, Jacobsen, Røe, & Gjerstad, 2015) and predict the future course of CBP (Schistad et al., 2014).

A second, related aim of this study is to investigate pain learning-related brain function in CBP patients. Chronic pain is closely tied to dysfunctional pain expectancy and pain avoidance learning (Gatchel, Peng, Peters, Fuchs, & Turk, 2007; Jensen, Ehde, & Day, 2016). Recent work from our group (Roy et al., 2014) and others (Delgado, Li, Schiller, & Phelps, 2008; Eldar, Hauser, Dayan, & Dolan, 2016; Seymour et al., 2005) has begun to identify the prefrontal, striatal, and brainstem systems supporting pain avoidance learning in healthy samples using fMRI. Broad differences in these brain systems have also been observed in chronic pain (Baliki et al., 2012; Bushnell, Ceko, & Low, 2013; Hashmi et al., 2013; Lee et al., 2015; Seminowicz et al., 2011; Vachon-Presseau et al., 2016), but their specific role in chronic pain is unknown. Here, we propose to investigate alterations in prefrontal-striatal-brainstem systems supporting pain avoidance learning in chronic pain patients. This area has been relatively understudied. Advances along this front may make critical contributions to our understanding of chronic pain. We additionally will test whether the treatment alters these pain avoidance learning systems, relative to waitlist.

Additionally, electroencephalography (EEG) provides a potentially promising measurement channel for developing brain markers of pain intensity (Davis et al., 2017). EEG markers of pain intensity have expanded translational value relative to fMRI, as EEG is cheap, portable, quick, and easily administered. Existing work points to altered EEG signals in chronic pain (Ploner, Sorg, & Gross, 2016). However, previous studies have not investigated EEG responses to different *intensities* of evoked chronic back pain. Here, we measure EEG while evoking back pain of different intensities, which may facilitate the development of an EEG marker for chronic pain intensity.

III. PRELIMINARY STUDIES

Our laboratory has extensive expertise in functional magnetic resonance imaging of placebo analgesia and pain (Geuter & Büchel, 2013; Geuter, Eippert, Hindi Attar, & Büchel, 2013; Wager et al., 2004, 2013; Wager, Atlas, Leotti, & Rilling, 2011). A previous study from our laboratory demonstrated placebo analgesia in acute experimental pain even when participants know the treatment is inert (a placebo) (Schafer et al., 2015). We have also conducted numerous studies administering acute pain stimulations to participants (Atlas, Bolger, Lindquist, & Wager, 2010; Colloca, Petrovic, Wager, Ingvar, & Benedetti, 2010; Koban & Wager, 2015), including a currently ongoing study administering pressure pain stimulations with the same device as proposed here (IRB protocol #15-0483). Co-I Sona Dimidjian has much experience conducting randomized controlled psychotherapy trials (i.e., Dimidjian et al., 2006, 2015; Shallcross et al., 2015), and Co-I Howard Schubiner has previously conducted randomized controlled trials of psychotherapy for chronic pain (Hsu et al., 2010). And, our other collaborators have previously used EEG to identify brain correlates of chronic pain (Prichep, Shah, Merkin, & Hiesiger, 2017).

IV. RESEARCH STUDY DESIGN

Study design: The study design is presented in the following figure. Participants with chronic back pain will complete an online prescreen. Those who are likely eligible will then be randomized to one of two parallel studies, with randomization stratified on pain intensity, age, gender, and opioid use. The first study compares a placebo treatment to waitlist, and the second study compares a psychotherapy treatment to waitlist (Figure, panel A). We do not use a standard three-way randomization (psychotherapy vs. placebo vs. waitlist) since we want participants receiving the placebo treatment to believe they are getting *the active treatment*, not a control treatment (which is the usual function of the placebo in a study). Beliefs and expectations are central to placebo effects (Ashar, Chang, & Wager, 2017), so we constrain participant's expectations in the placebo vs. waitlist study to necessarily think they are in the active treatment arm.

Patients in both studies will undergo identical study procedures (Figure, panel B). The one exception is which treatment they receive—placebo injection or a psychotherapy treatment, depending on to which study they are randomized. Thus, for the remainder of this protocol, we discuss both studies as one. We simply refer to **"the treatment"** to refer to placebo or to psychotherapy (depending on the study). The only place we describe the two studies separately is regarding the treatment procedures; all other descriptions apply identically to both studies.

We plan to combine data from both studies in the data analysis phase, since patients in both studies complete identical study procedures (besides the treatment administered). To allocate subjects efficiently, patients will be randomized 2:1 treatment to waitlist. This will result in three equally sized groups—placebo, psychotherapy, and waitlist—for the analysis phase.

After patients are randomized to study, we will call them and describe the study, including which treatment they might receive. Interested patients will then complete an in-person eligibility session, and eligible participants will be scheduled for the baseline assessment session. Following the baseline assessment session, participants will then be randomized to the treatment group or the waitlist group (with a ratio of 2:1 treatment:waitlist), using a computer-generated random sequence. The wait-list group is needed to isolate treatment effects from the effects of time and any effects associated with simply enrolling in a study. Thus, patients are **randomized twice:** first to study (placebo or psychotherapy study), and then, within study, to treatment vs. waitlist.

Participants randomized to treatment will be scheduled for treatment. Approximately one month after the baseline assessment session, all participants will return for a second assessment. After

this second, post-treatment assessment, participants who were on the waitlist in the placebo study will be offered the option of receiving the open-label placebo treatment. Participants who were on the waitlist in the psychotherapy study will be offered a chronic pain self-help book (Unlearning your Pain, written by Howard Schubiner) and free access to an online chronic pain self-help program based on the psychotherapy treatment (total market value: ~$125). Receiving the treatment will be optional for waitlisted participants.

A brief follow-up survey will be sent at months 1, 2, 3, 6, and 12 after the final assessment session. These will provide longer term data about the trajectory and durability of patient improvement.

Additionally, a group of healthy controls, with no history of back pain, will complete the baseline assessment (Figure, panel C). They will serve as a comparison group for brain activity related to the novel back pain device and task described below. Healthy controls will have only one in-person session.

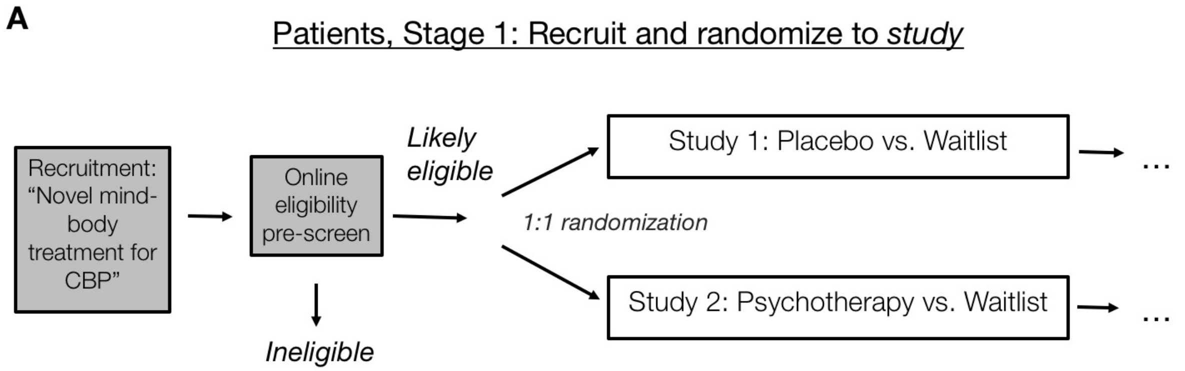

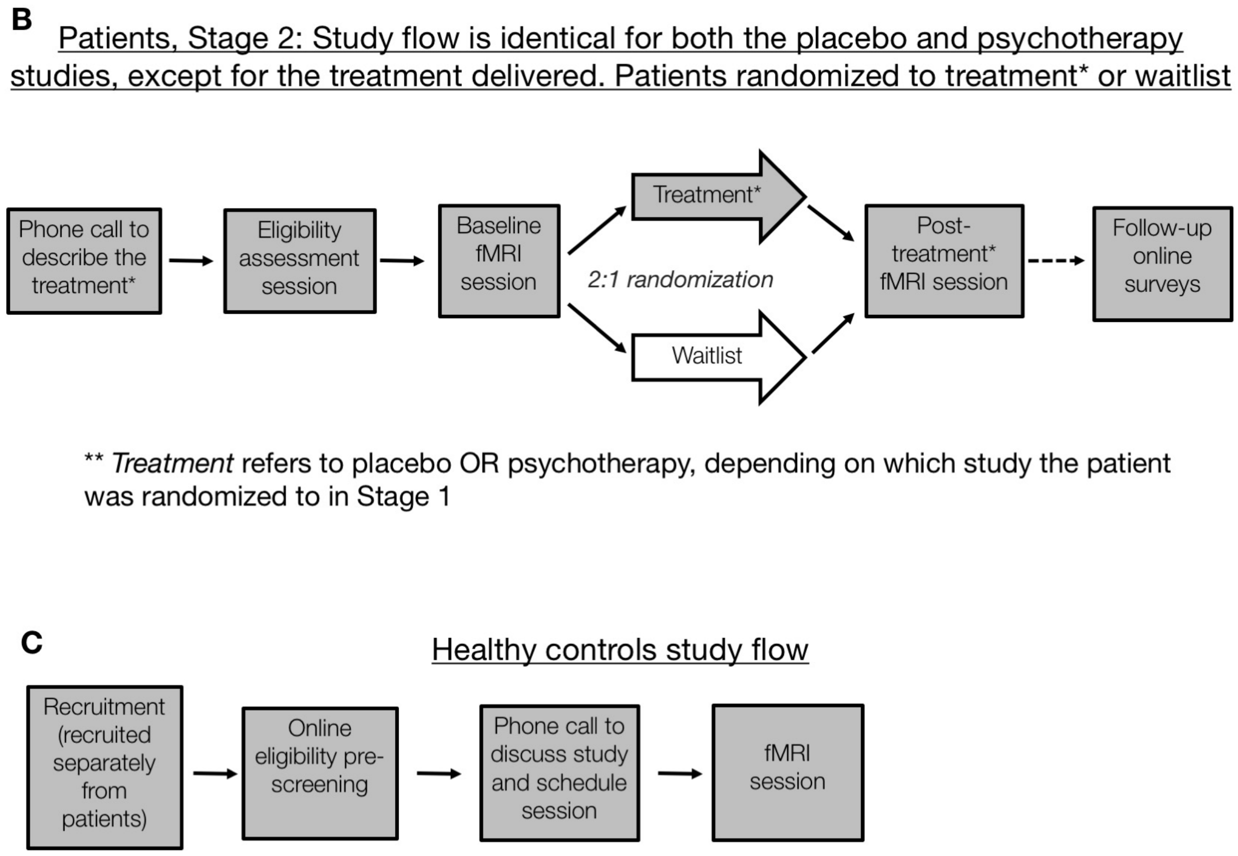

Figure. Study design. A) First stage of randomization: patients are randomized to study. B)

A description of each session is provided in the Procedures section below.

Sample size: We will randomize N = 150 patients in the study, 50 per treatment group. Since we estimate a ~15% rate of attrition/technical problems, we estimate this will yield a sample of N = 126 completers (about N = 42 per group). We estimate that an additional N = 25 patients will be enrolled in the study but will not be randomized to a treatment group for a variety of reasons (e.g. no shows to the baseline scan, decisions to withdraw after the baseline scan). Additionally, we will enroll N = 65 healthy controls to target N = 50 controls with high quality fMRI data.

Power analysis: Effect size estimates come from two sources. First, four previous trials comparing sham acupuncture (i.e., placebo) to no treatment found an average placebo effect size of d = -0.68 (95% CI -0.85 to -0.50) (Hróbjartsson & Gøtzsche, 2010). Second, a previous trial of open-label placebo for irritable bowel syndrome found an effect of d = -.79 relative to no treatment (Kaptchuk et al., 2010). These two effect sizes require two groups each of sizes n = 44 and n = 33, respectively for each effect size estimate, to yield 80% power at two-tailed α = .05. Thus, with a sample of N = 42 completers per group, we estimate that our study will have approximately 80% power, assuming the effect size of the placebo treatment in this study is in the range of what was reported in these previous studies. The psychotherapy is expected to have an effect size at least as large as the placebo treatment, since psychotherapy includes placebo effects in addition to other treatment components (Ashar et al., 2017).

Study length: The study will take 2 - 3 months for each participant (not including follow-up online surveys, the last of which will be sent 1 year later), and 2 years to complete data collection.

Data analysis plan: The primary outcome is pain intensity, as measured by the Brief Pain Inventory – Short Form (BPI-SF). We choose pain intensity as the primary outcome as it is the main target for both the placebo treatment and the psychotherapy, and pain intensity may be most relatable to the brain measures collected. We will use the mean value of the four BPI-SF pain intensity questions, or, if the mean BPI-SF score is highly correlated with the single BPI-SF item measuring average pain over the last week, we will use that single "average pain" item. The "average pain" item is a more interpretable outcome to a broad audience (Pek et al., 2018), and it aligns with our operationalization of "remission" below.

The primary endpoint of the trial is the post-treatment MRI session, approximately 5 weeks after the baseline assessment and randomization. Follow up measures are collected for one year following the post-treatment assessment and may be included in the main analysis or presented in a separate manuscript. Secondary analyses will investigate pain intensity each week during the treatment phase, to explore the time courses of treatment responses. One question of special interest is the durability of the placebo response (Ashar et al., 2017). Pain reduction may peak soon after the placebo treatment and diminish by the time of the primary endpoint, perhaps because a common lay concept of steroid injections is that they provide time-limited pain relief.

Treatment effects will be estimated using mixed models, which are considered to be well suited for handling missing data. For all analyses, waitlisted participants from both studies (i.e., psychotherapy waitlist and placebo waitlist patients) will be combined into one waitlist group. Secondary outcomes will be analyzed in a similar manner as the primary outcome.

Clinical significance will be determined by calculating the percentage of patients in each condition who meet the following criteria regarding pain reduction from baseline: at least 30% reduction in pain intensity, at least 50% reduction in pain intensity, and "pain free or nearly pain free"—defined as average pain intensity of 0 or 1 over the past week (on the single BPI-SF item measuring average pain over the last week).

Primary analyses will be conducted on an intent-to-treat (ITT) sample, and if necessary, the ITT sample will be modified by excluding any randomized patients who are found not to meet study criteria (e.g., patients unable or unwilling to initiate the treatment or control group they are randomized to). Secondary analyses will be conducted on a per protocol ("completer") sample.

We will test three contrasts of particular theoretical interest rather than conducting an omnibus test (Rosenthal et al, 2000; Steiger 2004; Judd et al. 2009). The three planned comparisons are: 1) psychotherapy vs. placebo, to estimate psychotherapy effects relative to a control treatment, 2) placebo vs. waitlist, to estimate placebo effects, and 3) psychotherapy vs. waitlist, which provides a benchmark for comparing our findings to the broader literature, and which may be the most practically relevant question (e.g., what benefit might a patient expect from enrolling in this psychotherapy). Reporting of results will emphasize estimation of effect size rather than rejection of null hypotheses (Cumming, 2013; Pek et al. 2018).

To create study groups that are matched across covariates of no interest we will use a minimization procedure. Minimization is regarded as an optimum assignment procedure for studies with relatively

small sample sizes that seek to balance groups on several covarites (Hu, Hu, Ma, & Rosenberger, 2014; Lin & Su, 2012). Covariates that will be balanced on are baseline pain, age, gender, and opioid use. The minimization algorithm we will use (Xiao, Yank, & Ma, 2012) handles continuous covariates (age, pain), so these variables will not be stratified. We will control for baseline scores of these covariates in analyses, as described above, although the minimization algorithm is likely to evenly balance these covariates across groups. We will minimize patients twice: first, to study; second, to treatment or waitlist within-study (Figure 1). Although disability is a co-primary outcome measure, we do not balance group assignment on disability. This is because it is not measured in the eligibility pre-screening form (since it is not an inclusion criteria), and thus cannot be used to assign patients to study, which happens upon completion of the eligibility pre-screening form.

fMRI images will be preprocessed and analyzed using standard in-house tools (c.f. Wager et al., 2013) and other existing toolboxes (i.e., SPM, Conn). A secondary goal of the study is to identify patterns of brain activity that track subjective back pain intensity (pre-treatment) using a penalized regression model to handle correlated predictors (brain voxels). Our research group has developed several such patterns accurately tracking pain intensity (Wager et al., 2013) and other affective states (Ashar, Andrews-Hanna, Dimidjian, & Wager, 2016; Chang, Gianaros, Manuck, Krishnan, & Wager, 2015; Krishnan et al., 2016), and similar methods will be used to develop a back pain pattern in this study. Pattern intensity scores (indicating to what extent the back pain-related pattern of brain activity is expressed) will be submitted to a group by time interaction via a repeated-measures ANOVA test to test for treatment effects. Analyses of resting state data will follow previous studies of resting state brain function in chronic back pain (reviewed in Kregel et al., 2015). Additionally, we will conduct a group by time interaction for pain-related brain activity at each brain voxel independently, with corrections for multiple comparisons (Genovese, Lazar, & Nichols, 2002), as is standard practice in the field.

Another secondary goal is to determine the relationship between measures of immune function and chronic back pain, as various measures of immune function have been previously associated with chronic pain (see Background and Significance). These data be analyzed by Dr. Michael Irwin (UCLA) and Dr. Mark Hutchinson (University of Adelaide, Australia), who are both collaborators with expertise in this domain (Grace et al., 2014; Irwin & Miller, 2007; Kwok et al., 2013). We will send blood samples that have been de-identified (i.e., tagged only with a random ID, not with any identifying information). Only measures of immune system function will be extracted from these samples, and then they will be destroyed. The relationship between the measures of immune function and chronic back pain will be analyzed for group by time interactions via a repeated-measures ANOVA test. Primary markers of immune function that can be extracted from blood samples include IL-6 (E. M. Miller & McDade, 2012), and potentially other immune markers as well (Skogstrand et al., 2005) depending on the technology that will be available to our collaborators at the time of analysis. Age, gender, and opioid use will also be included as covariates in analyses of immune function due to potential influence of these factors on immune function.

Another secondary goal is to develop an EEG marker of chronic pain intensity. EEG data will be filtered and cleaned using standard procedures (e..g, Prichep et al., 2017). We will use machine learning techniques, as described for the fMRI analyses above, to identify patterns of brain activity across electrodes that predict pain intensity.

V. FUNDING

This research is being funded by the Study of the Therapeutic Encounter Foundation, the Radiological Society of North America, and by the Psychophysiologic Disorders Society.

VI. ABOUT THE SUBJECTS

Subject Population(s)	*Number to be randomized to each group*
**Chronic back pain community sample—placebo treatment**	50
**Chronic back pain community sample—placebo waitlist**	25
**Chronic back pain community sample—psychotherapy**	50
**Chronic back pain community sample—psychotherapy waitlist**	25
**Healthy controls**	25

We anticipate enrolling 240 participants (N = 150 patients randomized to treatment group, N = 25 patients who enroll but are never randomized, and N = 65 healthy controls), 176 of whom we anticipate will complete the study (N = 126 patients, N = 50 healthy controls completing).

Inclusion criteria:

- Participants aged 21 to 70 with CBP will be enrolled.
- CBP will be defined according to the criteria established by a recent NIH task force (Deyo et al., 2014). Pain duration must be at least 3 months, with back pain being an ongoing problem for at least half the days of the last 6 months. That is, patients can meet criteria by either reporting pain every day for the past 3 months, or by reporting pain on half or more of the days for the past 6+ months. This will be determined by asking patients: (1) How long has back pain has been an ongoing problem for you? (2) How often has low back pain been an ongoing problem for you over the past 6 months? A response of greater than 3 months to question 1 and a response of ''at least half the days in the past 6 months'' to question 2 would define CBP.
- Patients must rate pain intensity at 4/10 or greater on the Brief Pain Inventory-Short Form (BPI-SF) (on the item measuring average pain over the last week), in keeping with inclusion criteria from previous CBP trials (Baliki et al., 2012; Cherkin et al., 2016; Hashmi et al., 2013; Seminowicz et al., 2011). Since pain intensity fluctuates from week to week for many CBP patients, we will assess patients' pain intensity twice (at the online pre-screen and a few days prior to their eligibility session, described further below) to increase the likelihood that patients' pain intensity is at least 4/10 at the baseline pre-treatment brain scan. Patients must endorse 4/10 pain at both these measurements to be eligible. After endorsing 4/10 pain at both these measurements, changes in pain report at later study time points will not serve as basis for exclusion.
- Back pain must be elicited by our back pain device (see below).
- Participants must also be comfortable and able to communicate via email or text message, as several study measures are collected in this manner (see below).

Exclusion criteria:

- Back pain associated with compensation or litigation issues as determined by self-report within the past year.
- Leg pain is greater than back pain. This suggests neuropathic pain, which may be less responsive to placebo or psychotherapy.
- Difficulty participating for technical/logistical issues (e.g., unable to get to assessment sessions).
- Self-reported diagnoses of schizophrenia, multiple personality disorder, or dissociative identity disorder.
- Self-reported use of intravenous drugs, due to concerns about infections and subject compliance with experimental protocols.
- Inability to undergo MRI as determined by MRI safety screen (e.g., pregnancy, metal in body, claustrophobia, using the standard screen conducted by the MRI imaging facility).
- Hypersensitive or hyposensitive to pressure pain: unable to tolerate $7kg/cm^2$ stimulation or reporting no pain for $4kg/cm^2$ stimulation; see further details below.
- Current regular use of an immunosuppressant drug, such as steroids. Such drugs interfere with immunoassay results.
- Self-reported history of metastasizing cancers—cancer of the breast, thyroid, lung, kidney, prostate or blood cancers.
- Self-reported history of stroke, brain surgery, or brain tumor.
- Self-reported diagnosis of a specific inflammatory disorder: rheumatoid arthritis, polymyalgia rheumatica, scleroderma, Lupus, or polymyositis.
- Unexplained, unintended weight loss of 20 lbs. or more in the past year.
- Cauda Equina syndrome, as screened for by self-reported inability to control bowel or bladder function.

Patients enrolled in the study will continue their normal pharmacological and psychosocial treatment for pain. Patients will also agree not to change, add, or remove any of their current treatments during course of study, unless indicated otherwise by their physician. Patients will also agree not to make any large lifestyle changes (e.g., diet or exercise) during the course of the study. Patients will be asked to notify the study team of changes to their medication and treatments.

We will also recruit a healthy control group as a comparison group for the back pain fMRI task and other tasks. Inclusion and exclusion criteria for healthy controls are identical to inclusion/exclusion criteria for patients, besides back pain. Healthy controls will be enrolled to match the patient group on mean age and on gender ratio.

Inclusion criteria for healthy controls:

- Aged 21 – 70.
- Participants must also be comfortable and able to communicate via email or text message.

Exclusion criteria for healthy controls:

- Self-reported history of back pain lasting more than 2 weeks.
- Self-reported history of any chronic pain diagnosis.
- Difficulty participating for technical/logistical issues (e.g., unable to get to assessment sessions).
- Self-reported diagnoses of schizophrenia, multiple personality disorder, or dissociative identity disorder.

- Self-reported use of intravenous drugs, due to concerns about infections and subject compliance with experimental protocols.
- Inability to undergo MRI as determined by MRI safety screen (e.g., pregnancy, metal in body, claustrophobia, using the standard screen conducted by the MRI imaging facility).
- Hypersensitive or hyposensitive to pressure pain: unable to tolerate 7kg/cm^2 stimulation or reporting no pain for 4kg/cm^2 stimulation; see further details below.
- Current regular use of an immunosuppressant drug, such as steroids. Such drugs interfere with immunoassay results.
- Self-reported history of metastasizing cancers—cancer of the breast, thyroid, lung, kidney, prostate or blood cancers.
- Self-reported history of stroke, brain surgery, or brain tumor.
- Self-reported diagnosis of a specific inflammatory disorder: rheumatoid arthritis, polymyalgia rheumatica, scleroderma, Lupus, or polymyositis.
- Unexplained, unintended weight loss of 20 lbs. or more in the past year.
- Cauda Equina syndrome, as screened for by self-reported inability to control bowel or bladder function.

VII. VULNERABLE POPULATIONS

None.

VIII. RECRUITMENT METHODS

Patients will be recruited in four ways: 1) referral from pain management providers, 2) advertisement via flyers, electronic bulletin boards, local listservs relevant to chronic pain (i.e., chronic pain patient groups listervs), local newspapers, and social media sites such as Facebook (described further below), 3) direct contact: patients who contact our research group inquiring about chronic pain research will be informed about the present study, 4) advertising through Google (described further below).

We attach to this protocol seven recruitment materials: a flyer, an online posting, a newspaper ad, a Facebook ad, a pamphlet for providers to give interested patients, the Google advertisement,and a screen capture of a study webpage providing an overview of the study and a link to the pre-screening document. Recruitment materials will describe the treatment as a "novel mind-body treatment". The nature of the treatment (placebo or psychotherapy, depending on participant randomization) will be explained to patients when they come in for the eligibility session (see below) to avoid confusion and misunderstanding, given the unusual and potentially confusing nature of placebo treatments. The flyer contains a link, which will take participants to a webpage that provides a brief overview of the study as well as a link to the REDCap online eligibility pre-screening document.

Facebook advertising will target subjects in the Boulder-Denver area in the eligible age range. Ads may be targeted to subjects who are "known or likely buyers of pain relief products" as determined by Facebook analyses that allow advertisers to target specific populations. The Facebook ad will link to a webpage that provides a brief overview of the study a well as a link to the REDCap online eligibility pre-screening document. The Facebook ad will be posted by a Facebook Page created for the study. This Page will display only approved recruitment materials (i.e., approved flyers and recruitment blurbs). The

Facebook ad will be as attached (please see Facebook Ad.pdf), and the back pain image will be randomly chosen from stock back pain images provided by Facebook (please see Facebook Example Images.pdf).

We will also advertise using the Google AdWords platform. The advertisement (attached) will be shown to subjects in the Boulder-Denver area who are searching on Google for terms related to back pain (e.g., “sore back”, “back pain relief”). The Google ad will link to a webpage that provides a brief overview of the study as well as a link to the REDCap online eligibility pre-screening document. The Google ad includes only text, no images.

Individuals who contact our study will be sent an online eligibility pre-screen form (see attached), which was designed in accordance with the Pre-Screening Guidance Document. Patients who pass the pre-screen (i.e., who endorse at least 4/10 pain and meet other study criteria) will be randomized to either the placebo study or the psychotherapy study and will be scheduled for an eligibility session. At the eligibility session, the “Provider pamphlet” document will be used to explain what a placebo is to patients in the Placebo study and how it might relieve back pain. This is the first randomization for patients. Later, patients are randomized a second time, to treatment or waitlist, described further below and in Study Design above.

Patients who do not pass the pre-screen will be emailed a list of treatment referral options (attached to this protocol). Patients who provided ambiguous information on the online pre-screen may be called or emailed with clarifying questions. Dr. Sona Dimidjian, a licensed psychotherapist, will provide clinical supervision as needed for these calls when related to mental health conditions.

Healthy controls will be recruited through the general recruitment protocol used in the Wager lab (approved IRB protocol #10-0243, title: Screening protocol for pain studies). This protocol describes the recruitment and screening of healthy subjects for pain fMRI studies, which describes the experience of healthy controls in this study. This protocol describes recruitment through fliers on university bulletin boards, university mailing lists (e.g., Buff Bulletin), newspaper ads and online bulletin boards such as craigslist. Potential subjects will be directed to email Screening Coordinators for more information and to undergo eligibility prescreening with a REDCap survey.

Undue influence and coercion are avoided because subjects must initially contact us, and then must complete the online pre-screen of their own volition in their own homes, with no pressure from the study team. During the eligibility session, it will be emphasized that participants are free not to continue with the study, and that they may also take some time to think about whether they want to participate.

List recruitment methods/materials and attach a copy of each in eRA
1. **Flyer**
2. **Online bulletin board posting**
3. **Pamphlet for providers to give to potential participants with referral information**
**4. Facebook recruiting**
**5. Local newspapers**
**6. Local email listservs**

IX. COMPENSATION

All participants will be paid $50 for an eligibility session with EEG or $25 for an eligibility session without EEG, $75 for each MRI session, and $5 for each follow-up survey completed (total of 5 follow-up surveys sent). Participants would thus earn up to $225 for completing this study with EEG, or $200 without EEG. Participants will be paid only for visits they have completed. Participants deemed ineligible or decide they are not interested in the study at the eligibility session will be paid $25 or $50 if they completed the EEG collection.

Participants will also be given an option to receive a 3D print-out of their brain instead of the $75 payment for one of the MRI sessions. Our lab has recently developed the capacity for 3D printing of brains. The market cost of a 3D brain print-out is over $250 (for people who already have a brain image, which we will give to our participants freely). Participants who choose this option would thus be paid up to $125 and will receive a 3D brain printout as well.

Participants can also earn several dollars at each assessment session from the progressive ratio button pressing task and from the willingness to experience pain task. The exact amount will vary depending on participants' choices, but is estimated to be $0 - $5 for the progressive ratio task and is $0 - $10 for the willingness to experience pain task, at each of the pre- and post-treatment assessment sessions.

All payment will be in cash given at the conclusion of the participant's final assessment session, except for the payment for the follow-up surveys, which will be in the form of an Amazon.com gift card sent electronically or through the mail, depending on participant preference. We use Amazon.com gift cards because they are substantially simpler to disburse from grant funds relative to creating personal checks for each participant. A single payment for all completed follow-up surveys will be disbursed after the final 12-month survey.

Healthy controls will be paid $75, or instead can choose to receive a 3D print-out of their brain.

X. CONSENT PROCESS

Participants will be seated in a private room in CINC and given a paper copy of the consent form. Each study has its own consent form—one for placebo vs. waitlist participants and one for psychotherapy vs. waitlist participants. Additionally, healthy controls have their own consent form. A member of the research team will also give a verbal explanation of the purpose, procedures, risks and benefits of the study. Participants will be given a chance to read the consent form and ask any questions. The informed consent form for the MIND data sharing initiative (attached) will also be explained and offered to participants, giving them the option of sharing their data with the MIND research network (see also below). Participants will also be asked to note whether the study personnel have permission to recontact them regarding future studies. Coercion and undue influence will be minimized by reminding participants that their participation is voluntary, and that the placebo treatment is unusual and not appropriate for everyone. Regarding EEG collection, the benefits (e.g., contributing to scientific research), the additional compensation, and the added burdens (e.g., some gel residue in hair, increased time commitment) will be explained to participants, and they will freely choose whether to participate in the EEG collection.

XI. PROCESS TO DOCUMENT CONSENT IN WRITING

Participants and a member of the research team will both sign the consent form.

XII. PROCEDURES

Research sites: All data collection will be conducted at the Center for Innovation and Creativity at 1777 Exposition Dr., Boulder, CO 80301. This building houses the Intermountain Neuroimaging Consortium MRI facility. The MRI device is FDA approved for research with human subjects and has all the safety inherent in a clinical MRI scanner. The radio frequency fields conform to guidelines determined by the FDA and the FDA has designated MRI scanners to be a non-significant risk device. The MRI scanning will be performed according to the safety and procedural standards of INC.

The placebo treatment will be administered at the Panorama Orthopedics and Spine Center in Golden, CO, by Dr. Karen Knight or at the Wardenburg Clinical Translational Research Center (CTRC) with Dr. Thomas Flood MD PhD. Psychotherapy will be conducted in rooms at CINC appropriately set up for this purpose (i.e., comfortable seating, white-noise machines outside the doors).

The study procedures described below are identical for patients in the psychotherapy study and patients in the placebo study. The only point of divergence is in the treatment delivered. Study procedures for healthy controls are described separately later in this section.

Pre-eligibility session verification of pain severity: Approximately 5 days prior to their scheduled eligibility session, patients will be sent a BPI-SF. If they endorse less than 4/10 average pain intensity over the last week, the patient's eligibility session will be cancelled along with an explanation that based on their latest responses they are not eligible. If patients endorse at least 4/10 pain on this measure, their pain report at the eligibility session will not be used as the basis for exclusion (even if they report less than 4/10 at that time). If patients fail to complete this pre-eligibility session BPI-SF, the BPI-SF collected at the eligibility session will be used as a basis for inclusion/exclusion. Our motivation for administering this additional online measurement *before* patients come in to the eligibility session is to save patients' and study time and resources, as the BPI-SF can easily be measured remotely and does not require patients to come in in-person only to be quickly told they are not eligible, after completing our first survey.

Informed consent and eligibility assessment session: Informed consent will be obtained as described above, with participants signing a placebo consent form or a psychotherapy consent form, as appropriate. We will then confirm that a back pain device we have recently developed does elicit back pain for the patient.

The back pain device consists of three parts: an inflatable bladder that is placed under the patient's back, a controller which controls inflation level using a pressure regulator, and a pressurized air tank (see attachment 'Back Pain Device.pdf'). The device design is based on a thumb pressure pain device which our lab has used in several studies with no adverse events. The back pain device is functionally equivalent to placing a small pillow under patients' backs, where the pillow thickness can be dynamically controlled. Pilot studies with N = 10 patients have confirmed that this device is painful for many back pain patients, the pain is tolerable, and the pain returns to baseline almost immediately after the stimulation ends. No pilot patient reported any adverse events or lingering pain 1 hour or 1 day after

testing. We have consulted with physicians with expertise in back pain (Co-I Dr. Howard Schubiner and collaborator Dr. Rachael Rzasa Lynn), who have confirmed that this device cannot cause damage to patients backs, since laying with a pillow under one's back is fundamentally innocuous though it may be painful. The largest bladder we will use will have a 6" diameter. At any point, if participants indicate that pain is above the level they are willing to tolerate, we will discontinue participation.

The bladder controller is programmed such that it cannot inflate beyond 0.25 kg/cm^2 units of pressure. The bladders cannot burst at this pressure level, which is relatively low (i.e., the bladders still have a fair amount of give when pressing on them at this inflation level). In tests manually inflating the bladder to higher levels, the bladder developed a leak only at 7 kg/cm^2 (more than 25 times the maximum pressure which the controller can administer). Even at this inflation level, the bladder did not burst, but began to slowly leak air from a seam. Thus, we believe there no risk of bladder bursting. If unexpected external pressure were placed on the bladder, it might either a) fail to maintain the desired inflation level, or b) develop a small leak along a seam, neither of which pose a risk to participants. We are using this back pain device simply to elicit a pain response. We are not collecting data on its safety or efficacy for submission to the FDA.

To calibrate the back pain device for each patient and determine their eligibility, we will ask patients to lay on the bladder while it is inflated and deflated to various inflation levels for approximately 12 minutes. We will start at the lowest inflation level, so patients can withdraw if desired without experiencing higher stimulation levels. During inflations and deflations, patients will be asked to continuously report their pain using a trackball or similar device. These continuous pain ratings will be used for analysis of the EEG data collected during the task for patients who consent to such (see below), and for developing models of continuous pain experience elicited by the bladder that will be applied to the fMRI data. Pain report is expected to vary substantially between patients, due to variable baseline (pre-existing) levels of pain and to variable responses to the device. Patients who report no additional pain with this device will be deemed ineligible and thanked and paid.

During this back pain calibration task, we will collect electroencephalography (EEG) on participants who consent to such. EEG will be recorded using a standard EEG data acquisition device (devices provided by Evoke Neuroscience) using an electrode cap (from ANT Neuro) which places electrodes on the scalp over the 19 scalp locations of the International 10/20 Electrode Placement System, referenced to linked earlobes. Electrodes in the cap are filled with an electrode gel to facilitate connection to the scalp. Data will be sampled at a rate of 200 Hz with 12 bit resolution. Patients will be monitored during EEG recording. We will also collect 20 minutes of eyes closed resting state EEG as a reference scan. EEG collection is optional, and patients may decline to do EEG for any reason (e.g., do not want gel in their hair, do not have the time available). Declining EEG will not impact participation in the study in any other way. Compensation provided for the eligibility session will be higher for patients completing EEG, since the EEG adds a substantial time demand to the eligibility session.

Participants who consent to EEG will also be asked to complete a "tone tracking" control task. Participants will listen to a tone whose volume will fluctuate over approximately 12 minutes. Participants will be asked to continuously rate the loudness of the tone. The tone will never be painfully loud; its loudness will remain in the silent to moderately loud range. The tone frequency will have a relatively low frequency, such that it is in the audible range even for people with the loss of high frequency hearing. This task will control for several aspects of the back pain intensity rating task, including estimating sensory intensity and the sensorimotor demands of making continuous ratings. Participants who do not complete EEG will not be asked to complete this tone tracking task.

We will also conduct a pressure pain task to a) determine whether the participant is *hyper*-sensitive or *hypo*-sensitive to pressure pain stimulations and thus ineligible for our study, and b) to familiarize participants with the procedures and device and show them how to terminate the stimulation should they need to. We will administer different levels of pressure pain stimuli in a random order (between 4 and 7 kg/cm^2, max duration = 10 seconds) and ask participants to rate their pain. Inclusion based upon hyper- or hypo-sensitivity to painful stimulations will be done using the following criterion: We will include participants who report that the stimulations are painful (i.e., not non-painful) but tolerable. We will also administer an 8 kg/cm^2 pressure stimulation to ensure that the participant can remove his or her thumb from the pressure device and knows how to do so (see "RISKS TO PARTICIPANTS" and "MANAGEMENT OF RISKS"). We will include participants who can safely remove their thumb from the device. At any point, if participants indicate that pain is above the level they are willing to tolerate, we will discontinue participation.

During the consent process, when describing the sound pain task to participants, we will play participants the aversive sounds they will hear during fMRI scanning. This will minimize coercion by allowing potential participants to refuse participation prior to granting consent if the sound is unacceptable to them.

Eligible patients will be scheduled for the baseline assessment session. Ineligible patients will be thanked, paid, and given a list of treatment referral options. Eligible patients will also complete several questionnaires listed in the table below, under investigation as potential moderators of treatment response.

Eligible patients will also be asked to complete a task asking them what feelings they currently have and where they feel them in their body (if felt in the body). Recent research has revealed that many feelings have reliable spatial locations in the body (Nummenmaa, Glerean, Hari, & Hietanen, 2013). Since chronic pain patients have altered emotional functioning (Lumley et al., 2011), we hypothesize that they may also have altered bodily correlates of emotions or altered ability to locate emotion in their body. This "body mapping" task will be completed using a smartphone application, which provides an ideal interface for locating feelings in the body (see Figure 2 below). Participants click an "I feel" button to display a list of feelings (i.e., happiness, sadness, pain, fear, stress, etc.), and then can drag a feeling bubble to a bodily location (or to a location indicating "not felt in the body"), and then rate the intensity of the feeling on a visual analog scale.

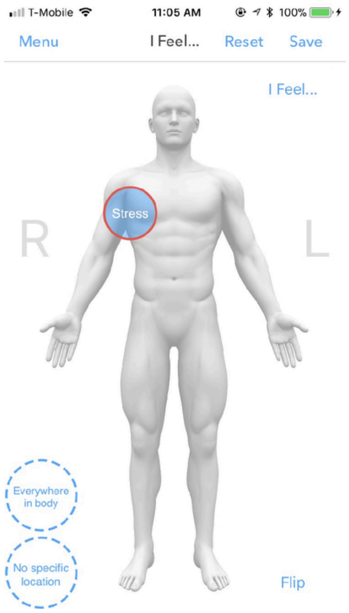

Figure 2. Screenshot of CliexaEase body mapping smartphone application.

Additionally, the application will ask participants to enter their current medications. We are interested in medications most specifically because of the current opioid crisis, and the potential of the mind-body treatments under investigation here to reduce opioid use. The application has an interface that makes it easy for participants to choose their medications from a dropdown list and to choose their dosage from commonly prescribed options.

The application, called CliexaEase, is developed by collaborators of ours and is freely available on application marketplaces for both Android and iPhone. We will ask participants to download the application to their smartphone. This element of the study is optional: If participants do not have a smartphone, or for any other reason prefer not to do this, this will not impact their study participation in any way.

The application will save participants' random study ID, their body maps of emotions as they report them, and their current medication use. This information will be saved to Cliexa servers, from which we will download it for our study analysis purposes. No identifying information will be captured by the app, and we will not otherwise provide Cliexa with any identifying information about our participants, and Cliexa will not attempt to identify any data (as attested to in the attached data sharing agreement). We will explain this arrangement to participants: that the application developer will receive no identifying information, only body maps tagged with random IDs.

Eligible participants will also be asked whether they would like to serve as scheduled back-up participants. Serving as a back-up is optional and has no bearing on any other aspects of their participation in the study. Back-up participants will be asked to arrive at the same time as another scheduled participant (the "primary" participant). If the primary participant fails to show, or is unable to be scanned for an unexpected reason (i.e., they got a tattoo since last study contact and did not notify the study team), then the back-up participant will be scanned instead. If the primary participant does show and is able to be scanned as expected, the back-up will be thanked, paid $25, and sent home. This determination will be made approximately 1 hour and 10 minutes after the back-up is asked to arrive, once the scan for the primary participant is successfully underway. Participants who would like to serve

as back-ups may also have a "primary" scan scheduled for them at a future date (after which they can no longer serve as back-ups), or, they may continue serving as a back-up indefinitely, and will have their primary scan scheduled only when enrollment nears its end.

3 days before the baseline assessment: For the 3 days before the baseline assessment, participants will be emailed or texted (according to their preference) once per day with a link to the Brief Pain Inventory – Short Form (BPI-SF), a primary outcome measure in our study. We conduct this additional measurement because previous research suggests that repeated daily measurement of outcome measures may be preferable relative to a single measurement collected in the laboratory (R. C. Moore, Depp, Wetherell, & Lenze, 2016). We also repeat this procedure for the 3 days after the baseline assessment, yielding a total of 7 pre-treatment measurements of the BPI-SF: 3 days before the baseline session, at the baseline session, and 3 days after the baseline session.

Baseline assessment: Each session will begin with welcoming participants, reminding them of the plan for that session, and addressing any initial questions. Participants will then complete self-report questionnaires (listed in the table below) at a computer, using the REDCap platform (see Data Management below), and will be asked to do a body mapping of their current feelings and their current medications using the Cliexa smartphone app as described above.

Participants will then complete a computerized version of a progressive ratio task previously found to be altered in chronic pain (Schwartz et al., 2014). In this task, participants can earn a fixed amount of money ($1) for a progressively increasing amount of effort. Effort will be operationalized by button presses. For the first $1, participants will have to complete 50 button presses. For each additional $1, the number of button presses will be increased by a factor of 2.3 (i.e., 115, 265, 608, etc.). Participants are free to stop at any time or will be stopped once 20 minutes have elapsed.

Participants will then be escorted to the MRI scanner. Prior to going into the MRI scanner, the MRI technologist on duty will ask participants to remove all jewelry and metal objects from their pockets. Participants will be required to change into scrubs to prevent any possible risk from metallic objects or decorations in their clothing.

During fMRI scanning, participants will complete several functional MRI tasks. Tasks completed during functional MRI include:

1. A scan during which participants are asked to rate their ongoing pain intensity using a visual analog scale every 30 – 60 seconds. We will use pillows and blankets to make participants as comfortable as possible during this scan to minimize back pain.
2. A second scan during which participants are asked to rate their ongoing pain intensity using a visual analog scale every 30 – 60 seconds. During this scan, we will elicit back pain using the inflation levels that different pain intensities in the eligibility session. Maximum exposure time to the bladder in the scanner will be 15 minutes.
3. An acute pain and aversive sounds task. Participants will receive a pseudo-randomized sequence of pressure pain stimulations and unpleasant sound stimulations (i.e., the sound of a knife moving across glass). Pressure stimulations will be administered to the left thumbnail. Pressure pain stimulations will be administered using a pressure pain device (see attachment "Pressure pain device") and will be within the safe range based on our pilot data and previous studies (see attachments "Pressure pain pilot study results" and "Pressure pain stimulation guideline"). Aversive sounds will serve as a control condition for non-pain related negative affect. These aversive sounds will be taken from a widely used standardized database of

unpleasant sounds, the International Affective Digital Sounds (IADS) database, and will be mildly to moderately unpleasant. Another ongoing study (IRB protocol #15-0483) has run over 50 participants to date with both the thumb pressure device and the aversive sounds with no adverse events. Following stimulations, participants will be asked to rate the intensity and/or unpleasantness of the pain or sound.

We will also collect heart rate and respiration during the fMRI scans using MRI-compatible devices routinely used at CINC. These physiological measures will be used for de-noising the fMRI data.

A venous blood sample with be collected at this session and again at the post-treatment assessment scan. The blood sample will consist of an upper extremity blood draw via standard venipuncture technique (https://www.ncbi.nlm.nih.gov/books/NBK138650/pdf/Bookshelf_NBK138650.pdf, 2017). 8cc of blood will be collected per draw (16cc total/subject over the entire study). All study personnel performing the blood draws with be trained by Dr. Thomas Flood in accordance with the 'WHO guidelines on drawing blood: best practices in phlebotomy' (https://www.ncbi.nlm.nih.gov/books/NBK138650/pdf/Bookshelf_NBK138650.pdf, 2017). Dr. Flood is a senior radiology resident with the University of Colorado Diagnostic Radiology Residency (post-graduate year 5) with clinical training in numerous medical procedures, including blood draws. Since Colorado state law does not require a certification for performing blood draws, the WHO guidelines will serve as our standards for compliance. Areas of phlebotomy training will include: anatomy of acceptable sites to be used for venous sampling, infection prevention and control, protection of the study subject, protection of the study personnel working with blood samples, types and proper use of equipment, practice obtaining blood samples, adverse events/occupational exposure and management, waste management (including disposal of sharps and biologic waste, and procedures for spillage/breakage), and proper labelling, handling, and transportation of samples. All study personnel will demonstrate appropriate skill and mastery of the training content prior to performing venous blood draws on study subjects. The risks of blood draws via standard venipuncture technique are minimal and include pain, bruising at the venipuncture site, redness and swelling of the vein and infection, and a rare risk of vagovasal response.

If there is a contraindication to performing the blood draw (i.e., cutaneous infection or scarring at the site of sampling, difficulty locating a vein, excessive fear/phobia of needles that the subject had not previously reported during informed consent), a blood spot will be collected instead. The blood spot will be collected with a finger stick collection device sold by Spot On Sciences HemaSpot™ device. The device draws 2 -3 drops of blood from the fingertip, is mildly painful, and has minimal associated risks. All assessment sessions for a given participant will be scheduled at approximately the same time of day, because of the influence of circadian rhythm on biological measures. Blood samples will be used to measure the immune markers IL-1, IL-6, and IL-10.

Participants will also complete a task designed to measure **willingness to experience pain**, which has been identified as a maintenance factor in chronic back pain. Participants will be given a $10 endowment for this task, which they can keep or spend as they choose. They will place bids in a series of Becker-DeGroot-Marschak (BDM) (Becker, Degroot, & Marschak, 1964) auctions on how much not experiencing pain is worth to them. Participants will be asked to bid a dollar amount ($0 - $10, in $1 increments) on how much they would like to not experience pain of X minutes. The duration of pain (X minutes) will range from 0 to 10 minutes and will be randomly varied across trials. After the last trial, one of the trials will be randomly chosen to be implemented as a BDM auction. A random number will be chosen. If the random number is larger than the participant's bid, the participant will keep the full

$10 and experiences the pain indicated on that trial. If the random number is smaller than the participant's bid, the participant will not experience any pain. Instead, they must pay back the amount bid and keep the remainder of the endowment. The optimal strategy in such auctions is to bid the true value of not experiencing pain for X minutes, and participants will be informed of this. We will also carefully explain this task to participants and provide an opportunity for questions, given the potentially confusing nature of this auction. Similar BDM auctions for experiencing pain have been previously conducted (Vlaev et al., 2012; Vlaev, Seymour, Dolan, & Chater, 2009; Winston, Vlaev, Seymour, Chater, & Dolan, 2014). But here, we will do this with chronic pain. The pain administered will be the bladder inflation level eliciting approximately 5/10 intensity back pain.

Randomization: At the end of the baseline session, we will randomize patients to treatment or wait-list. Randomization will be stratified by baseline pain severity, age, gender, and opioid use. Patients will be randomized at a 2:1 ratio of treatment to waitlist, as described in the Study Design. Patients in the placebo study randomized to treatment will receive the placebo injection. Patients in the psychotherapy study randomized to treatment will start psychotherapy.

For 3 days after the baseline assessment: For the 3 days after the baseline assessment, participants will be emailed or texted (according to their preference) once per day with a link to the Brief Pain Inventory – Short Form (BPI-SF).

Treatment: Treatment details are described separately for each study below.

Treatment (placebo study): The placebo treatment session will be with Dr. Karen Knight at the Panorama Orthopedics and Spine Center in Golden, CO or with Dr. Thomas Flood MD PhD at the Wardenburg Clinical Translational Research Center (CTRC). Dr. Knight is a physical medicine and rehabilitation specialist with broad experience in both rehabilitative medicine and pain management. She is board certified in Physical Medicine & Rehabilitation with additional certifications in Sports Medicine, Integrative Medicine and Pain Medicine. She has received several awards and honors for her clinical work, including being listed as a 5280 Top Doc several times, receiving the National Bernard Baruch Medal, and the Vitals Patients' Choice Award. She has over a decade of experience in administering therapeutic injections for pain management. Dr. Thomas Flood is a senior radiology resident with the University of Colorado Diagnostic Radiology Residency (post-graduate year 5). Dr. Flood has clinical training in numerous medical procedures, including the proper administration of subcutaneous injections. Dr. Flood has extensive research training with numerous peer-reviewed first-author publications in basic, translational, and clinical research. Dr. Flood was awarded a competitive grant from the Radiological Society of North American to carry out the research outlined in this proposal. Dr. Flood's malpractice insurance covers his practice at the CTRC. An IAA has been arranged for COMIRB (which covers Dr. Flood) to cede to the CU Boulder IRB to cover Dr. Flood's engagement in this research.

To schedule patients, we will send a secure email (from University of Colorado email servers to a secure email server at the Panorama clinic) to the clinic front desk with demographic and contact information of patients who are ready to be scheduled for treatment (demographic information is required for patients to be entered into the clinic's computer system). All Panorama clinic staff are HIPPA trained and compliant and will handle participants' data as protected health information. During the informed consent process, patients will sign a HIPPA form allowing the release of PHI to the clinic. The clinic will

then contact patients and schedule them. Dr. Flood is a co-investigator on the current proposal and will schedule subjects at the CTRC, according to HIPPA compliant procedures.

The open-label placebo treatment we will use is based on past open-label placebo trials (Kam-Hansen et al., 2014; Kaptchuk et al., 2010; Kelley et al., 2012). Prior to treatment administration, patients will view a brief (~3 min) video summarizing scientific findings regarding the therapeutic power of placebo treatments. The video will describe established findings regarding placebo and suggest that placebos may still work even when patients know the treatment is a placebo. The placebo treatment will consist of a subcutaneous injection of saline into the participant's lower back according to the following procedure. The physician will wash their hands (either with soap and water or an alcohol-based hand rub) before entering and leaving the procedure room. The placebo injection procedure will be described to the participant and verbal acknowledgment of understanding of the procedure will be obtained; the opportunity to ask questions and obtain clarifying information will be given. The participant will also be informed of the procedural steps (as described below), concurrently as they occur. Next, the participant will be asked to lay prone on the examining table. The participant's lower back will be exposed, with the use of proper draping technique. The participant's exposed skin will be examined for signs of any dermatologic abnormality, which may be a contraindication to the injection (as detailed below). The participant will be asked to point to the area of localized pain. The location of the subcutaneous injection will be selected in proximity to the area of subjective pain within the soft tissues of the lower back (lateral to the midline). Prior to administering the injection, the physician will again wash their hands (with soap and water or an alcohol-based hand rub) and put on non-sterile nitrile examination gloves. Additional supplies include: a 10 ml disposable single-use vial of sterile, nonpyrogenic, isotonic, medical grade sodium chloride and water solution (0.9% sodium chloride; the solution will not contain any bacteriostatic/antimicrobial agents, added buffer, or preservatives), a sterile deposable syringe (1 ml) and needle (27 gauge; ½ -1 inch in length), a sterile 2x2 inch gauze pad, alcohol pads, a "sharps" container for syringe and needle disposal, and a Band-Aid. The vial of saline will be opened and the top will be cleansed with an alcohol pad. The syringe/needle will be opened and 1 ml of saline will be withdrawn from the vial to fill the syringe; air bubbles will be removed. The participant's skin (at the area of injection) will be cleansed with an alcohol pad and allowed to air dry. Then the needle will be introduced into the subcutaneous tissue of the lower back and 1ml of saline will be injected (using a slow steady rate of injection). The needle will be removed, sterile gauze will be gently applied to the site of injection, and then a Band-Aid will be placed. The needle/syringe will be disposed of in an appropriate "sharps" container located within the procedure room. Contraindications to the procedure include: an excessive fear/phobia of needles, an inability to arrive at the treatment site on their own, or a significant cutaneous abnormality that is located within proximity to the lower back injection site, including signs of infection (erythema, warmth, swelling, induration), an active wound, scar tissue, or other significant dermatologic abnormality. Following the treatment, the Panorama clinic will send us a secure email saying patient with initials YZ (for example) got the treatment on X date.

Treatment (psychotherapy study): Psychotherapy will consist of an initial medical history session with Co-I Schubiner, a brief intake phone call with lead psychotherapist Alan Gordon, and twice weekly 50 minute psychotherapy sessions for 4 weeks with a therapist.

The purpose of the initial medical history session is to help evaluate the likelihood that the patient's back pain is caused by structural conditions in the back. No patients will be excluded as a result of this evaluation. Patients will send Dr. Schubiner any medical or radiological reports they have available using secure fax or secure email provided by Dr. Schubiner's hospital. Patients will complete a HIPAA authorization during the informed consent process to cover this sharing of PHI. Dr. Schubiner will then

speak with patients for a 1 hour session in which he collects their medical history and discusses different possible causes of their back pain with them. This session will be conducted by phone or by HIPAA-compliant videoconferencing technology arranged in consultation with the OIT team at Dr. Schubiner's hospital. Patients can conduct this session at any location they prefer, and will be offered to do it from a private room in CINC if they would like. Dr. Schubiner has conducted hundreds of such assessments in the context of his clinical practice and in previous research trials (Hsu et al., 2010). He will not provide patients with a formal diagnosis but with a diagnostic impression of the causes of their pain according to his clinical judgment. Dr. Schubiner will prepare a brief report regarding his impressions for each patient that will be stored in REDCap and will be accessible by therapists. Dr. Schubiner is licensed to practice medicine in Colorado (pro bono license).

Alan Gordon (lead psychotherapist) will then have a brief phone call with the patient (~10 minutes) orienting them to the psychotherapeutic approach and scheduling their first psychotherapy session.

Psychotherapists in this trial will be from the Pain Psychology Center (www.painpsychologycenter.com), a well-established private practice clinic in Los Angeles with expertise in psychotherapy for CBP. Therapists will come to Boulder to see patients for the study. Alan Gordon, LCSW and executive director of the Pain Psychology Center, will supervise all therapists in weekly supervision and case consultation meetings. All therapists will be licensed to practice in Colorado and will be experienced in the treatment of CBP. The psychotherapy incorporates techniques from established modalities with known efficacy, including cognitive behavioral therapy (CBT) and acceptance-based approaches. Additionally, leveraging the medical history report, therapists will help patients consider whether their back pain is caused by structural conditions in the back, and if it is not, whether the pain truly signals damage to the back. While this component is present to some in existing psychotherapies, the emphasis on it here presents a novelty of our psychotherapeutic approach. The therapists seeing patients include: Alan Gordon, Daniel Lyman, Jonathan Ashar, Christie Uipi, and Ed Walton. Dr. Schubiner will be available to therapists for consultation in case of back-related medical questions. Dr. Dimidjian will also provide consultation as needed, given her extensive experience in psychotherapy research trials.

Psychotherapy sessions will be video-taped, for later coding of therapist and client behaviors, as is standard procedure in psychotherapy trials. Recent advances in affective computing have allowed the automatic detection of emotional experience from facial expression, opening new methods for probing affective experience during psychotherapy. These tools require capturing facial features that also make subjects identifiable from the videos. For patients who consent, excerpts from videos may be shown for educational purposes (i.e., at conference presentations). At any point, patients may ask the therapist to stop the video and the therapist will comply. Patients will be told at the outset that they can request this at any time.

In the rare event that a therapist must unexpectedly leave Boulder or is unable to continue in-person treatment, the patient will be transferred to a different therapist. However, if the patient is near the end of treatment, the patient and therapist may agree to conduct the last sessions over phone or secure videoconferencing instead. The patient will be given the option of transferring therapists or continuing with the same therapist via secure videoconferencing.

Brief weekly surveys during the treatment phase (this and following procedures again apply to all patients in both studies): All patients will be sent a weekly questionnaire between treatment initiation and the post-treatment assessment session. For waitlisted participants, questionnaires will start

approximately one week after the baseline assessment session. Participants will be emailed or texted (according to their preference) a few brief surveys as listed in the table below.

Daily surveys for 3 days before post-treatment session: As at the baseline assessment, for 3 days before the post-treatment assessment, participants will be emailed or texted (according to their preference) once per day with a link to the to the Brief Pain Inventory – Short Form (BPI-SF).

Post-treatment assessment: About 4 weeks after initiating treatment (i.e., the medical history session for psychotherapy patients or receiving the injection for placebo patients), all patients will return to complete a second assessment. Waitlisted patients will return about a month after the baseline session. This session is identical to the baseline assessment, but with two additional questionnaires administered (the PGIC and the treatment satisfaction questionnaire).

Patients will then be asked if they would be willing to speak on camera about their experiences with the treatment, in their own words. Qualitative measures such as this can detect important information that quantitative measures may completely miss, and they can offer a different view on the effect of treatment (i.e., Allen, Bromley, Kuyken, & Sonnenberg, 2009; K. M. Moore & Martin, 2015). We collect this qualitative data to supplement the many quantitative measures already being collected. If patients agree, a member of the research team will conduct a semi-structured interview asking about their history of back pain, their sense of whether the treatment was of benefit, and what they think was most and least helpful about the treatment they received. The interview will be video recorded in a study testing room affording privacy to the patient. The interview is optional and declining will not impact study participation in any way. It will take 10 – 15 minutes, and we will offer participants some snacks and drinks prior to the interview, as it is at the end of a 3-hour session and they may appreciate the refreshments. Participants who agree to the interview will be asked to indicate allowed uses of the recording on a form (see attached). They will be administered this form immediately prior to the interview, rather than during the initial consent process, so that they can make a decision better informed by their study experience thus far. Participants who completed the study prior to the addition of this interview to the study protocol will be invited to come in for this interview. They will be paid $30 for doing so. Declining to come in for the interview will not impact their participation in the study in any other way.

Daily surveys for 3 days after post-treatment session: For 3 days after the post-treatment assessment, participants will be emailed or texted (according to their preference) once per day with a link to the to the Brief Pain Inventory – Short Form (BPI-SF). This yields a total of 7 post-treatment measurements of the BPI-SF: 3 days before the post-treatment session, at the session, and 3 days after the post-treatment session.

Offering treatment to wait-listed patients: Wait-listed patients in the placebo injection study will be offered the opportunity to receive the placebo treatment (optional). Waitlisted participants in the psychotherapy study will be given a copy of Dr. Schubiner's book and free access to his online self-help program (optional to accept these).

Follow-up: At months 1, 2, 3, 6, and 12 after their final assessment, all patients will be emailed an online survey including the self-report outcome measures (listed in table below), along with a request to open the CliexaEase application on their smartphone and complete a body mapping of their current feelings. We will attempt up to 4 total contact attempts via phone or email over a maximum of 3 weeks to patients who do not respond to the survey.

Healthy Controls

Healthy controls will be recruited through the Wager lab general screening protocol (approved IRB protocol #10-0243). This protocol covers the recruitment and screening of healthy subjects for pain fMRI experiments, which describes the experience of healthy controls in the current study. Participants recruited through this protocol complete an online eligibility pre-screening REDCap survey regarding MRI eligibility and basic demographic and contact information.

Participants who are likely eligible based on this form will be called. The procedures for healthy controls in our study will be briefly explained to them. We will describe the general purpose of the study as investigating the brain bases of chronic back pain, and explain to these participants that they will serve as a healthy control group for back pain patients.

If participants are interested, we will ask them some additional questions about inclusion/exclusion criteria that are not covered by the general lab screening protocol. The experimenter will read a list of yes/no questions, and instruct the participant to say after the last question whether the answer was 'yes' to any of the questions that had been read. This procedure will help protect participants' privacy in leaving unclear the exact reason for exclusion. The questions are:

- Have you ever had back pain lasting more than 2 weeks?
- Have you ever been diagnosed with a chronic pain condition?
- Have you ever been diagnosed with schizophrenia, multiple personality disorder, or dissociative identity disorder?
- Have you ever had a metastasizing cancers—a cancer of the breast, thyroid, lung, kidney, prostate or blood cancer?
- Have you ever had a stroke, brain surgery, or brain tumor?
- Have you ever been diagnosed with rheumatoid arthritis, polymyalgia rheumatica, scleroderma, Lupus, or polymyositis?
- Have you had an unexplained, unintended weight loss of 20 lbs. or more in the past year?
- Do you use intravenous drugs?
- Do you regularly use of an immunosuppressant drug, such as steroids?
- Do you have difficulty controlling bowel or bladder function?

We are also in the process of submitting an amendment to the Wager lab general screening form protocol to ask the above questions so we do not need to do so by phone. Once this amendment is approved and participants and completing the updated screening form, we will no longer need to ask these questions by phone.

Participants who are interested and likely eligible will be scheduled for an fMRI session. This session is similar to the eligibility and baseline assessment sessions for patients described above. It consists of the following procedures:

1. Informed consent
2. The thumb pressure pain eligibility task, described above, to ensure that tolerable pain is elicited. If the device is not painful or is intolerably painful, participants will be excluded. Based on previous experience with this device, very few participants will be excluded for this reason.

3. Back pain device familiarization. This will follow the procedure described above for patients, in which we expose participants to different inflation levels and ask them to provide continuous ratings of pain experience. Unlike patients, controls will not be excluded based on pain report or lack thereof. The device is not expected to cause more than mild back pain for healthy controls, as pilot subjects with no history of back pain (n = 5) reported no pain or mild pain at the maximum inflation level. EEG will not be collected on healthy controls during this task, as it is for patients only, and healthy controls will not complete the tone tracking control task.
4. Questionnaires at a computer, using REDCap. Questionnaires are listed below.
5. Participants will be asked to download the CliexaEase application and complete a body mapping of their current feelings (optional) and to list current medications.
6. fMRI scanning session identical to that completed by patients, described above.
7. The progressive ratio task. (Healthy controls will not do the willingness to experience pain task, because the back pain device will not be painful for most of these participants.)
8. A single blood sample will be collected: a serum draw, or if contraindicated for the reasons listed above, a blood spot.
9. Participants will be thanked and paid.

Healthy control participants who are interested and eligible will also be asked whether they would like to serve as scheduled back-up participants. Serving as a back-up is optional and has no bearing on any other aspects of their participation in the study. Back-up participants will be asked to arrive at the same time as another scheduled participant (the "primary" participant). If the primary participant fails to show, or is unable to be scanned for an unexpected reason (i.e., they got a tattoo since last study contact and did not notify the study team), then the back-up participant will be scanned instead. If the primary participant does show and is able to be scanned as expected, the back-up will be thanked, paid $25, and sent home. This determination will be made approximately 1 hour and 10 minutes after the back-up is asked to arrive, once the scan for the primary participant is successfully underway. Participants who would like to serve as back-ups may also have a "primary" scan scheduled for them at a future date (after which they can no longer serve as back-ups), or, they may continue serving as a back-up indefinitely, and will have their primary scan scheduled only when enrollment nears its end.

Name of instrument/tool/procedure	*Purpose (i.e. what data is being collected?*	*Time to Complete*
**Positive and Negative Affect Scale short form (PANAS-SF) ("panas 10" file)**	*Secondary outcome measure*	1 minute
**Brief Pain Inventory—short form (BPI-SF)**	*Co-primary outcome measure and administered weekly during treatment*	1 minute
**Oswestry Low Back Pain Disability Questionnaire (OLBPD)**	*Co-primary outcome measure*	4 minutes
**PROMIS short forms: anger (5 items), sleep disturbance (8 items),**	*Secondary outcome measure and*	2 minutes altogether

**anxiety (8 items) and depression (8 items)**	*administered weekly during treatment*	
**Tampa Scale of Kinesiophobia (TSK)**	*Secondary outcome measure and administered weekly during treatment*	1 minute
**Survey of Pain Attitudes Short Form, Emotion subscale (SOPA-Emo)**	*Secondary outcome measure and administered weekly during treatment*	< 1 minute
**Patient Global Impression of Change (PGIC) scale**	*Post-treatment-only outcome measure*	1 minute
**Treatment satisfaction questionnaire**	*Post-treatment-only outcome measure*	1 minute
**Semi-structured interview about treatment experience**	*Post-treatment-only outcome measure*	10 – 15 minutes
**Pain Catastrophizing Questionnaire (PCS)**	*Secondary outcome measure*	2 minutes
**Fear of Pain Questionnaire**	*Potential moderator*	3 minutes
**General Self-Efficacy Scale (GES)**	*Potential moderator of treatment effect*	2 minutes
**Mindful Attention Awareness Scale (MAAS)**	*Potential moderator of treatment effect*	2 minutes
**Emotion Regulation Questionnaire (ERQ)**	*Potential moderator of treatment effect*	2 minutes
**Life Orientation Test-Revised (LOT-R)**	*Potential moderator of treatment effect*	1 minutes
**Timeline Follow-Back Measure for alcohol and drug use (TLFB)**	*Secondary outcome measure*	8 minutes
**Treatment history form, asking how effective previous injection treatments have been for patients' CBP**	*Potential moderator of treatment effect*	1 minutes
**Demographic information**	*Potential moderator of treatment effect*	1 minutes
**Adverse Childhood Experiences questionnaire (ACE)**	*Potential moderator of treatment effect*	2 minutes
**BMQ-Specific modified**	*Potential moderator of treatment effect*	1 minute
**MRI**	*Outcome measure*	1 hour
**Dried blood spot**	*Outcome measure*	5 minutes
**Baseline and post-treatment daily surveys**	*To better measure treatment effect on primary clinical outcomes*	1 minute/day

**Progressive ratio button pressing task**	*Outcome measure*	0 – 20 minutes maximum, estimated mean of 5 minutes
**Willingness to experience pain task**	*Potential moderator and outcome measure*	5 – 15 minutes, estimated mean of 10 minutes
**Pressure pain eligibility task**	*Eligibility measure*	10 minutes
**Back pain elicitation eligibility task**	*Eligibility measure*	10 minutes
**Emotion body mapping and medication use, collected via smartphone application**	*Potential moderator and outcome measure*	5 minutes
**EEG**	*Potential brain marker of pain intensity*	50 minutes

The total time commitment for a subject is 8 hours over 1.5 – 2 months (not including follow up surveys which may add 1 hour over the following year). Psychotherapy subjects will have an additional 7 – 9 hour time commitment.

Visit #	***Procedures/Tools***	***Location***	***How much time the visit will take***
Online pre-screening	• *Online eligibility pre-screening form*	*online*	*15 minutes*
Pre-eligibility session online screen	• *BPI-SF*	*online*	*1 minute*
Eligibility session	• *Informed consent*<br>• *LOT-R*<br>• *ERQ*<br>• *GES*<br>• *TSK*<br>• *SOPA-Emo*<br>• *MAAS*<br>• *TLFB*<br>• *BPI-SF*<br>• *OLBPD*<br>• *IPQ*<br>• *Fear of Pain Questionnaire*<br>• *PCS*<br>• *PANAS-10*<br>• *ACE*<br>• *BMQ-Specific modified*	*CINC*	*1.5 hours without EEG, 2.5 hours with EEG*

	• *Treatment history form*<br>• *Demographic information*<br>• *Pressure pain eligibility task*<br>• *Back pain elicitation eligibility task*<br>• *Emotion body mapping and medication use*<br>• *EEG (optional)*		
Baseline assessment session	• *MRI session*<br>• *Blood sample*<br>• *Positive and Negative Affect Scale short form (PANAS-SF) ("panas 10" file)*<br>• *PROMIS short forms: anger, sleep disturbance, anxiety, and depression*<br>• *BPI-SF*<br>• *OLBPD*<br>• *TLFB*<br>• *TSK*<br>• *SOPA-Emo*<br>• *Fear of Pain Questionnaire*<br>• *BMQ-Specific modified*<br>• *Pain Catastrophizing Questionnaire (PCS)*<br>• *PROMIS short forms: anger, sleep disturbance, anxiety, and depression*<br>• *Emotion body mapping and medication use*	*CINC*	*3 hours*

	• *Progressive ratio button pressing task*<br>• *Willingness to experience pain task*		
Placebo treatment session	• *Subcutaneous injection*	*Panorama Orthopedics and Spine Center*	*30 minutes*
Psychotherapy treatment sessions	• *Initial medical history followed by twice weekly psychotherapy sessions for 4 weeks (8 sessions max)*	*CINC*	*1 hour per session = 7 – 9 hours total, depending on how many sessions a patient attends*
Weekly surveys during treatment phase	• *BPI-SF*<br>• *TSK*<br>• *SOPA-Emo*<br>• *PROMIS short forms: anger, sleep disturbance, anxiety, and depression*	*At home*	*10 minutes*

Post-treatment assessment session	*MRI session* *Blood sample* *Positive and Negative Affect Scale short form (PANAS-SF) (“panas 10” file)* *BPI-SF* *OLBPD* *TLFB* *TSK* *SOPA-Emo* *Fear of Pain Questionnaire* *Pain Catastrophizing Questionnaire (PCS)* *PROMIS short forms: anger, sleep disturbance, anxiety, and depression* *Patient Global Impression of Change (PGIC) scale* *Treatment satisfaction questionnaire* *Emotion body mapping and medication use* *Progressive ratio button pressing task* *Willingness to experience pain task* *Interview*	*CINC*	*3 hours, or 3 hours 15 minutes if agree to the interview*
6-month follow-up online survey	*Positive and Negative Affect Scale short form (PANAS-SF) (“panas 10” file)* *BPI-SF*		*10 – 15 minutes*

	- OLBPD - PROMIS short forms: anger, sleep disturbance, anxiety, and depression - TSK - Patient Global Impression of Change (PGIC) scale - Treatment satisfaction questionnaire - Emotion body mapping and medication use	
Session for healthy controls	- Informed consent - Demographic information - Pressure pain eligibility task - Back pain task	*3 hours*
	- Positive and Negative Affect Scale short form (PANAS-SF) ("panas 10" file) - BPI-SF - OLBPD - Pain Catastrophizing Questionnaire (PCS) - PROMIS short forms: anger, sleep disturbance, and depression - LOT-R - ERQ - Fear of Pain Questionnaire - MAAS - GES - MRI session	

- *Progressive ratio button pressing task*
- *Blood sample*

XIII. SPECIMEN MANAGEMENT

Blood samples will be stored in a locked cabinet on site (CINC) in a facility designated specifically for storing biological specimens or within a secure -80C research grade freezer at the CU Boulder main campus. All stored data will be coded with a randomly generated number, and the master list linking the numbers to participants' names will be stored on a password protected server. At study closure, all biological specimens will be destroyed. DNA will not be extracted from these specimens.

XIV. DATA MANAGEMENT

According to HRP-111, this is a low risk (Level 1) study.

Strict standards of confidentiality will be maintained. All self-report data will be collected with the REDCap platform managed by UC Denver. REDCap is a secure, widely used data collection tool. Only research team members will have access to the REDCap data platform. The only pen-and-paper data that will be collected is the informed consent form. This will be stored in a locked file cabinet in a locked room at CINC. Identifying information will be collected during the online pre-screen, which asks for participants' names, phone numbers, and email addresses. After study completion, this information will be removed from the REDCap database, permanently de-identifying the REDCap data.

The only exception to the above are the measures of emotion body mapping and current medications collected by the Cliexa smartphone application. This data will be tagged only with random IDs and does not constitute PHI. Cliexa will periodically send the data to the research team, who will save it on secure, password-protected servers. Cliexa will never receive any identifying information and will make no attempts to re-identify the data.

MRI data will be stored according to standard INC data management procedures. MRI images and psychotherapy session videos will be housed on a password protected CU Boulder server. Metadata (name and contact information) will be entered into the COINS database by study personnel. Each COINS entry will receive a unique research subject identifier. This code will be associated with the images. A copy of the MRI images will be sent to the Mind Research Network. No identifying information is included in the images.

EEG data will be collected using an Evoke Neuroscience EEG headset. The headset wirelessly transmits data back to the company's servers, tagged only with a random identifier. The data is then available for download by the research team. The data will also be available for download by our collaborators at PainQX, Inc. who may use it to develop EEG markers of pain intensity. For this purpose, we will share with PainQX measures of self-reported chronic pain intensity, as well as relevant covariates include depression, anxiety, age, and gender. We will not share identifying information with PainQX, and PainQX will make no effort to identify the data, as attested to in the attached form.

Medical and radiological reports sent to Dr. Schubiner will be stored on a password protected server or a locked file cabinet administered by his hospital accessible only to him.

The video recordings will all be collected on one camera which will be stored in a locked room between interviews. The recordings will be regularly downloaded from the camera, transferred to a secure, password-protected server accessible only to the research team, and deleted from the camera.

XV. WITHDRAWAL OF PARTICIPANTS

We may withdraw participants from the study if: we learn that they added, changed, or stopped any concurrent treatment for their pain (for example, undergoing back surgery mid-study); participants' back pain dramatically worsens and we judge they need more intensive treatment than that provided in this study.

Withdrawn subjects will have no further data collected from them, and there will be no follow up contact with them. If they request it in writing, we will delete all previously collected data linked to them. They will be replaced with new subjects.

XVI. RISKS TO PARTICIPANTS

Subcutaneous injection: subcutaneous injection has minimal risk of adverse events when delivered in accordance with guidelines (Annersten & Willman, 2005; https://www.cc.nih.gov/ccc/patient_education/pepubs/subq.pdf, 2017). Adverse events include bruising or hematoma at the site of injection, typically resolving within a few days (Annersten & Willman, 2005), and in very rare cases, infection. Risks will be minimized by following the injection procedure, (as detailed above; see the "Treatment (placebo study)" subsection within the "Research Design and Methods" section), and by not administering the lower back subcutaneous injection within proximity to a significant cutaneous abnormality, including signs of infection (erythema, warmth, swelling, induration), an active wound, scar tissue, or other significant dermatologic abnormality. Subcutaneous injections are performed as part of routine medical appointments. They are considered safe even for at-home self-administration (i.e., insulin for diabetics), although this will not be done in this study.

MRI: The risks of MRI are:

- The MRI may cause discomfort due to scanner noise.
- There may be some discomfort from lying still and in one position for a long time.
- Peripheral nerve stimulation (PNS/tingling). At sufficient exposure levels, peripheral nerve stimulation is perceptible as "tingling" or "tapping" sensations. PNS symptoms will usually subside shortly after the scan is completed.
- Participants may feel nervousness or feelings of claustrophobia.
- MRI may pose risks to fetuses, we thus exclude potentially pregnant women.

While the images are taken for research purposes, participants will be notified should an anomaly of clinical importance be observed, as is standard practice for INC/MRN images.

Injury due to pressure pain device malfunction: There is a very slight risk to the participant in case of pressure pain device malfunction. We have tested the device on 50 participants in an ongoing study and 12 in-lab participants (see attachment "Pressure pain pilot") without any incidents. In addition, we systematically tested if participants could readily remove their thumb from the device under high-pressure stimulation (e.g., 8 kg/cm^2) and specifically when pressure was experienced as too high to tolerate for an extended period. Eleven participants finished the test, and all were able to remove their thumb from the device. One participant terminated the experiment after the first few trials due to hypersensitivity to pressure. We also asked participants whether they experienced any long-term harmful effects of pressure pain. The survey showed that there was no remaining mark on the thumb after 3 hours for all participants who finished the test, and minimal tenderness was found after three hours. In addition, all participants who finished the test had no remaining sensation after three hours. The participant who discontinued the pilot experiment early reported discomfort for several hours after the experiment, but it disappeared within 24 hours. In the ongoing study (current N = 50), no adverse events have been reported. Therefore, we expect that most participants will experience no risk due to the pressure pain device.

Back pain device: The inflatable bladder can cause pain and psychological discomfort. The levels of pain elicited will be within participants' tolerable level and will not cause any damage to participants' backs. The back pain elicited will be similar to what they feel during the course of daily life. The bladder cannot inflate beyond a 6" diameter. Additionally, the device has a low maximum pressure (0.25 kg/cm^2) beyond which it will not inflate, so the bladder will not burst from pressure.

Risk of psychotherapy: Psychotherapy contains inherent risks (Dimidjian & Hollon, 2010). The therapy being tested here, while novel in some respects, is closely related to existing cognitive-behavioral and acceptance-based therapies that are widely used in research and practice. While systematic data on psychotherapy risks are lacking (Dimidjian & Hollon, 2010), it is widely believed that cognitive-behavioral and acceptance-based therapies pose a low level of risk to patients. A recent large, well-conducted trial systematically measured adverse events associated with two psychotherapies for CBP similar to the psychotherapy under investigation here. This trial reported that about one third of patients experienced a temporary increase in pain as a result of increased activity levels, and no other adverse events or serious adverse events were reported (Cherkin et al., 2016). In addition to temporary increases in pain, emotionally difficult material may arise during psychotherapy, which may cause psychological discomfort.

Risks of blood sample collection: The risks of blood draws via standard venipuncture technique are minimal and include pain, bruising at the venipuncture site, redness and swelling of the vein and infection, and a rare risk of vagovasal response. The dried blood spot kit involves a finger prick, which may cause mild discomfort. There is also a minor risk of bruising.

Risks of EEG: EEG is considered safe. The electrodes only measure electrical activity, and do not administer any stimulations, so there is no risk of electrical shock. Skin preparation for the electrode cap may cause minor discomfort.

Ethical concerns: There is no deception of either patients or treatment providers at any point during this study, and patients will not be prevented from seeking adjunctive treatment. We believe there are no ethical concerns present regarding this treatment.

XVII. MANAGEMENT OF RISKS

The risks associated with subcutaneous injection will be minimized by adhering to our injection procedure, (as detailed above; see the "Treatment (placebo study)" subsection within the "Research Design and Methods" section), adhering to standard safety protocols (i.e., Annersten et al., 2005; https://www.cc.nih.gov/ccc/patient_education/pepubs/subq.pdf, 2017), and by not administering the lower back subcutaneous injection within proximity to a significant cutaneous abnormality, including signs of infection (erythema, warmth, swelling, induration), an active wound, scar tissue, or other significant dermatologic abnormality.

The MRI-related risks will be managed in the following way. The MRI scan will be performed using an MR scanner employing pulse sequences and hardware that have been approved by the FDA for human clinical use. The field strength is 3 Tesla and all relevant operating characteristics (RF power deposition, rate of change of the field gradients, coil design) fall within the limits of FDA guidelines for NMR exposure. Participants will be carefully screened to exclude those who may have metal in or on their bodies that cannot be removed (e g., bullets, metal filings, body piercings, etc.). MR Facility rules strictly forbid staff from entering the magnet room carrying metal objects. Additionally,

- Discomfort from scanner noise will be minimized with high-quality noise-blocking earbuds.
- Discomfort from laying in the scanner will be minimized by making sure the subject is lying comfortably with head and neck supported.
- With regard to PNS, participants are given a squeeze ball to use in case of an emergency. They are informed that if they experience PNS related sensations or are otherwise uncomfortable, they can alert the MRI technologist via the squeeze ball and the technologist will stop the scan immediately.
- The risk of claustrophobia is minimized by screening subjects for self-reported claustrophobia. and, providing a mirror to see out, a button to signal distress, and an intercom.
- Pregnant women are excluded to minimize risk to the fetus. In accordance with standard INC procedures, female participants unsure as to whether they are pregnant will be given the opportunity to complete a urine pregnancy test immediately before the scanning period, and those with a positive result will not be scanned. Alternatively, female participants may sign a waiver (attached) that they do not believe themselves to be pregnant.

The risks associated with the pressure stimulations will be minimized in the following ways. We will exclude participants who are hypersensitive to pressure pain or have difficulty to remove their fingers from the device when pressure is high using the calibration procedure. In addition, the pressure pain software has a "Stop" button, which can be used to stop the pressure stimuli anytime by experimenters. The participants will be given a hand-squeezable pneumatic signaling device for communicating with experimenters during scanning and therefore should be able to signal intolerable discomfort of any kind. The device is regularly maintained and tested by our trained personnel. All personnel who use the equipment are trained on equipment procedures.

The risks associated with the back pain device will be minimized by performing an individual calibration for each participant testing how painful different levels of inflation are, as described above. Additionally, the device will be immediately deflated or removed from under participants' backs upon request. The device is functionally equivalent to placing a pillow or rolled towel under a patients' back—it may be painful but cannot cause damage to the back, as ascertained in consultations with pain physicians.

The risks associated with psychotherapy will be minimized by emphasizing to participants that psychotherapy participation is optional, and they can always choose not to attend sessions while still completing all other aspects of the study. Weekly supervision meetings among all therapists and the lead therapist, and a pain physician as needed, will also minimize risks by helping provide high quality of psychotherapeutic care to all patients in this study.

Risks of blood sample collection will be managed through phlebotomy training of study personnel to ensure the use of proper standard technique to prevent complications and adverse events such as infection. The risk of the blood spot will be managed by using an alcohol swab to clean the finger as well as a Band-Aid immediately after the blood collection.

The risks associated with EEG will be managed by training all research personnel in EEG recording protocols.

XVIII. POTENTIAL BENEFITS

Subjects may experience a reduction in back pain from the treatment.

The benefits to society stem from advancing scientific understanding of chronic pain, which imposes a large societal burden. This study will a) be the first to test the efficacy of an open-label placebo injection for chronic back pain, and will provide an estimate of treatment efficacy, b) be the first open-label placebo study to include biological outcome measures, which are needed to establish the efficacy of this treatment, and c) provide a novel understanding of brain mechanisms supporting pain learning in a chronic pain population. Additionally, this study will be the first to test the benefits of a psychotherapy with a novel psychoeducational component for CBP.

XIX. PROVISIONS TO MONITOR THE DATA FOR THE SAFETY OF PARTICIPANTS

A member of the research team will email or text subjects 3 days after receiving treatment to enquire about any adverse events. We have found that email/text is the most reliable way to reach most subjects (subjects often do not answer their phone when called). During the online pre-screening, we confirm with each subject that they are comfortable with email or text message communication, which is needed for completion of the daily surveys described above.

Jonathan Ashar will monitor these reports and be responsible for reporting adverse events to the IRB.

When half of the sample is collected, an interim analysis will be conducted on primary clinical outcome measures (i.e., PROMIS short forms). If the effect of treatment vs. waitlist is small (i.e., $d < .3$), the study may be halted or revised (with IRB approval).

XX. PROVISIONS TO PROTECT THE PRIVACY INTERESTS OF PARTICIPANTS

CINC is a large facility that a person might enter for any number of reasons, and it does not clearly associate participants with any condition or state. If participants are seen entering the Panorama Orthopedics and Spine Center, this will strongly suggest to an observer that they or a friend or family

member suffer from pain. This is unavoidable. Participants enroll in this study because they are seeking treatment for their pain, and for that reasons are presumably more than willing to enter a pain treatment clinic. All data collected will be coded with randomly assigned study IDs.

XXI. MEDICAL CARE AND COMPENSATION FOR INJURY

No research related injuries are expected. In the event one does occur, no compensation is available. This is standard for all our research protocols and other protocols that we are familiar with, and is stated clearly in the consent form. In case of any injury or discomfort we ask that participants contact us and will refer them to medical or psychological services as appropriate. For any complication resulting from the placebo treatment, we will refer them back to Dr. Karen Knight.

XXII. COST TO PARTICIPANTS

There will be no cost to participants. Parking at CINC and at the Anschutz Medical Campus is free.

XXIII. DRUG ADMINISTRATION

Only placebo will be used. The placebo will be 1mL of medical grade saline purchased from a supplier of medical equipment. This will be “normal” saline (0.9% NaCl).

XXIV. SHARING OF RESULTS WITH PARTICIPANTS

There are no plans to share research results with participants. Participants will be offered structural MRI images of their brain immediately after the second scan session.

References

Allen, M., Bromley, A., Kuyken, W., & Sonnenberg, S. J. (2009). Participants' experiences of mindfulness-based cognitive therapy: "It changed me in just about every way possible". *Behavioural and Cognitive Psychotherapy*, *37*(4), 413–30. http://doi.org/10.1017/S135246580999004X

Annersten, M., & Willman, A. (2005). Performing subcutaneous injections: A literature review. *Worldviews on Evidence-Based Nursing*, *2*(3), 122–130. http://doi.org/10.1111/j.1741-6787.2005.00030.x

Ashar, Y. K., Andrews-Hanna, J. R., Dimidjian, S., & Wager, T. D. (2016). Empathic Care and Distress: Predictive Brain Markers and Dissociable Brain Systems. *Neuron*, 1–11. http://doi.org/10.1016/j.neuron.2017.05.014

Ashar, Y. K., Chang, L. J., & Wager, T. D. (2017). Mechanisms of the placebo: an affective appraisal account. *Annual Reviews Clinical Psychology*.

Atlas, L. Y., Bolger, N., Lindquist, M. a, & Wager, T. D. (2010). Brain mediators of predictive cue effects on perceived pain. *The Journal of Neuroscience : The Official Journal of the Society for Neuroscience*, *30*(39), 12964–12977. http://doi.org/10.1523/JNEUROSCI.0057-10.2010

Baliki, M. N., Petre, B., Torbey, S., Herrmann, K. M., Huang, L., Schnitzer, T. J., … Apkarian, a V. (2012). Corticostriatal functional connectivity predicts transition to chronic back pain. *Nature Neuroscience*, *15*(8), 1117–1119. http://doi.org/10.1038/nn.3153

Becker, G. M., Degroot, M. H., & Marschak, J. (1964). Measuring utility by a single-response sequential method. *Behavioral Science*, *9*(3), 226–232. http://doi.org/10.1002/bs.3830090304

Bicket, M. C., Gupta, A., Brown, C. H. th, & Cohen, S. P. (2013). Epidural Injections for Spinal Pain: A Systematic Review and Meta-analysis Evaluating the "Control" Injections in Randomized Controlled Trials. *Anesthesiology*, *119*(4), 907–931. http://doi.org/10.1097/ALN.0b013e31829c2ddd

Blease, C., Colloca, L., & Kaptchuk, T. J. (2016). Are open-Label Placebos Ethical? Informed Consent and Ethical Equivocations. *Bioethics*, *0*(0), n/a-n/a. http://doi.org/10.1111/bioe.12245

Bushnell, M. C., Ceko, M., & Low, L. a. (2013). Cognitive and emotional control of pain and its disruption in chronic pain. *Nature Reviews. Neuroscience*, *14*(7), 502–11. http://doi.org/10.1038/nrn3516

Chang, L. J., Gianaros, P. J., Manuck, S. B., Krishnan, A., & Wager, T. D. (2015). A Sensitive and Specific Neural Signature for Picture-Induced Negative Affect. *PLOS Biology*, *13*(6), e1002180. http://doi.org/10.1371/journal.pbio.1002180

Cherkin, D. C., Sherman, K. J., Balderson, B. H., Cook, A. J., Anderson, M. L., Hawkes, R. J., … Turner, J. A. (2016). Effect of Mindfulness-Based Stress Reduction vs Cognitive Behavioral Therapy or Usual Care on Back Pain and Functional Limitations in Adults With Chronic Low Back Pain. *JAMA*, *315*(12), 1240. http://doi.org/10.1001/jama.2016.2323

Chou, R. (2007). Clinical Guidelines Diagnosis and Treatment of Low Back Pain : A Joint Clinical Practice Guideline from the American College of Physicians and the American Pain Society. *Annals of Internal Medicine*, (July).

Cohen, S. P., Argoff, C. E., & Carragee, E. J. (2008). Management of low back pain. *BMJ (Clinical Research Ed.)*, *337*(dec22_1), a2718. http://doi.org/10.1136/bmj.a2718

Colloca, L., Petrovic, P., Wager, T. D., Ingvar, M., & Benedetti, F. (2010). How the number of learning trials affects placebo and nocebo responses. *Pain*, *151*(2), 430–439. http://doi.org/10.1016/j.pain.2010.08.007

Cumming, G. (2013). The New Statistics: Why and How. *Psychological Science*, (November). http://doi.org/10.1177/0956797613504966

Davis, K. D., Flor, H., Greely, H. T., Iannetti, G. D., Mackey, S., Ploner, M., … Wager, T. D. (2017). Brain imaging tests for chronic pain: medical, legal and ethical issues and recommendations. *Nature Reviews Neurology*. http://doi.org/10.1038/nrneurol.2017.122

Delgado, M. R., Li, J., Schiller, D., & Phelps, E. A. (2008). The role of the striatum in aversive learning and aversive prediction errors. *Philosophical Transactions of the Royal Society of London. Series B, Biological Sciences*, *363*(1511), 3787–3800. http://doi.org/10.1098/rstb.2008.0161

Deyo, R. A., Dworkin, S. F., Amtmann, D., Andersson, G., Borenstein, D., Carragee, E., … Weiner, D. K. (2014). Report of the national institutes of health task force on research standards for chronic low back pain. *The Spine Journal*, *37*(7), 449–467. http://doi.org/10.1016/j.jmpt.2014.07.006

Dimidjian, S., Goodman, S. H., Felder, J. N., Gallop, R., Brown, A. P., & Beck, A. (2015). Staying Well During Pregnancy and the Postpartum: A Pilot Randomized Trial of Mindfulness-Based Cognitive Therapy for the Prevention of Depressive Relapse/Recurrence. *Journal of Consulting and Clinical Psychology*. http://doi.org/10.1037/ccp0000068

Dimidjian, S., & Hollon, S. D. (2010). How would we know if psychotherapy were harmful? *American Psychologist*, *65*(1), 21–33. http://doi.org/10.1037/a0017299

Dimidjian, S., Hollon, S. D., Dobson, K. S., Schmaling, K. B., Kohlenberg, R. J., Addis, M. E., … Jacobson, N. S. (2006). Randomized trial of behavioral activation, cognitive therapy, and antidepressant medication in the acute treatment of adults with major depression. *Journal of Consulting and Clinical Psychology*, *74*(4), 658–70. http://doi.org/10.1037/0022-006X.74.4.658

Eldar, E., Hauser, T. U., Dayan, P., & Dolan, R. J. (2016). Striatal structure and function predict individual biases in learning to avoid pain. *Proceedings of the National Academy of Sciences*, *113*(17), 201519829. http://doi.org/10.1073/pnas.1519829113

Freburger, J. K., Holmes, G. M., Agans, R. P., Jackman, A. M., Darter, J. D., Wallace, A. S., … Carey, T. (2009). The Rising Prevalence of Chronic Low Back Pain. *Arch Intern Med. February*, *9543*(1693), 251–258. http://doi.org/10.1001/archinternmed.2008.543

Gatchel, R. J., Peng, Y. B., Peters, M. L., Fuchs, P. N., & Turk, D. C. (2007). The biopsychosocial approach to chronic pain: Scientific advances and future directions. *Psychological Bulletin*, *133*(4), 581–624. http://doi.org/10.1037/0033-2909.133.4.581

Genovese, C. R., Lazar, N. a, & Nichols, T. (2002). Thresholding of statistical maps in functional neuroimaging using the false discovery rate. *NeuroImage*, *15*(4), 870–8. http://doi.org/10.1006/nimg.2001.1037

Geuter, S., & Büchel, C. (2013). Facilitation of pain in the human spinal cord by nocebo treatment. *The Journal of Neuroscience : The Official Journal of the Society for Neuroscience*, *33*(34), 13784–90. http://doi.org/10.1523/JNEUROSCI.2191-13.2013

Geuter, S., Eippert, F., Hindi Attar, C., & Büchel, C. (2013). Cortical and subcortical responses to high and low effective placebo treatments. *NeuroImage*, *67*, 227–236. http://doi.org/10.1016/j.neuroimage.2012.11.029

Grace, P. M., Hutchinson, M. R., Maier, S. F., & Watkins, L. R. (2014). Pathological pain and the neuroimmune interface. *Nat Rev Immunol*, *14*(4), 217–231. http://doi.org/10.1038/nri3621

Haake, M., Muller, H.-H., Schade-Brittinger, C., Basler, H. D., Schafer, H., Maier, C., … Molsberger, A. (2007). German Acupuncture Trials (GERAC) for Chronic Low Back Pain. *Archives of Internal Medicine*, *167*(17), 1892–1898.

Hashmi, J. a., Baliki, M. N., Huang, L., Baria, A. T., Torbey, S., Hermann, K. M., … Apkarian, a. V. (2013). Shape shifting pain: chronification of back pain shifts brain representation from nociceptive to emotional circuits. *Brain : A Journal of Neurology*, *136*(Pt 9), 2751–68. http://doi.org/10.1093/brain/awt211

Hashmi, J. a., Baria, A. T., Baliki, M. N., Huang, L., Schnitzer, T. J., & Apkarian, a. V. (2012). Brain

networks predicting placebo analgesia in a clinical trial for chronic back pain. *Pain*, *153*(12), 2393–2402. http://doi.org/10.1016/j.pain.2012.08.008

Hashmi, J. a, Baliki, M. N., Huang, L., Parks, E. L., Chanda, M. L., Schnitzer, T., & Apkarian, a. (2012). Lidocaine patch (5%) is no more potent than placebo in treating chronic back pain when tested in a randomised double blind placebo controlled brain imaging study. *Molecular Pain*, *8*, 29. http://doi.org/10.1186/1744-8069-8-29

Hoffman, B. M., Papas, R. K., Chatkoff, D. K., & Kerns, R. D. (2007). Meta-analysis of psychological interventions for chronic low back pain. *Health Psychology : Official Journal of the Division of Health Psychology, American Psychological Association*, *26*(1), 1–9. http://doi.org/10.1037/0278-6133.26.1.1

Hróbjartsson, A., & Gøtzsche, P. C. (2010). Placebo interventions for all clinical conditions. *Cochrane Database of Systematic Reviews*, *CD003974*(1). http://doi.org/10.1002/14651858.CD003974.pub3.www.cochranelibrary.com

Hsu, M. C., Schubiner, H., Lumley, M. A., Stracks, J. S., Clauw, D. J., & Williams, D. A. (2010). Sustained Pain Reduction Through Affective Self-awareness in Fibromyalgia: A Randomized Controlled Trial. *Journal of General Internal Medicine*, *25*(10), 1064–1070. http://doi.org/10.1007/s11606-010-1418-6

Hu, F., Hu, Y., Ma, Z., & Rosenberger, W. F. (2014). Adaptive randomization for balancing over covariates. *Wiley Interdisciplinary Reviews: Computational Statistics*, *6*(4), 288–303. http://doi.org/10.1002/wics.1309

Hull, S. C., Colloca, L., Andrew, A. A., Ziogas, D. C., Somkin, C. P., Kaptchuk, T. J., & Miller, F. G. (2013). Patients ' attitudes about the use of placebo treatments: telephone survey. *Bmj*, *3757*(July), 1–9. http://doi.org/10.1136/bmj.f3757

Irwin, M. R., & Miller, A. H. (2007). Depressive disorders and immunity: 20 years of progress and discovery. *Brain, Behavior, and Immunity*, *21*(4), 374–383. http://doi.org/10.1016/j.bbi.2007.01.010

Jensen, M. P., Ehde, D. M., & Day, M. A. (2016). The Behavioral Activation and Inhibition Systems: Implications for Understanding and Treating Chronic Pain. *The Journal of Pain*, *17*(5), 529.e1-529.e18. http://doi.org/10.1016/j.jpain.2016.02.001

Judd, C. M., Mcclelland, G., & Ryan, C. (2009). *Data Analysis: A Model Comparison Approach*. Routledge.

Kam-Hansen, S., Jakubowski, M., Kelley, J. M., Kirsch, I., Hoaglin, D. C., Kaptchuk, T. J., & Burstein, R. (2014). Altered placebo and drug labeling changes the outcome of episodic migraine attacks. *Science Translational Medicine*, *6*(218), 218ra5. http://doi.org/10.1126/scitranslmed.3006175

Kaptchuk, T. J. (2002). Academia and Clinic The Placebo Effect in Alternative Medicine : Can the Performance of a Healing Ritual Have Clinical Significance ?, (7), 817–825.

Kaptchuk, T. J., Friedlander, E., Kelley, J. M., Sanchez, M. N., Kokkotou, E., Singer, J. P., … Lembo, A. J. (2010). Placebos without deception: a randomized controlled trial in irritable bowel syndrome. *PloS One*, *5*(12), e15591. http://doi.org/10.1371/journal.pone.0015591

Kelley, J. M., Kaptchuk, T. J., Cusin, C., Lipkin, S., & Fava, M. (2012). Open-label placebo for major depressive disorder: a pilot randomized controlled trial. *Psychotherapy and Psychosomatics*, *81*(5), 312–4. http://doi.org/10.1159/000337053

Koban, L., & Wager, T. D. (2015). Beyond conformity: Social Influences on Pain Reports and Physiology. *Emotion*.

Kregel, J., Meeus, M., Malfliet, A., Dolphens, M., Danneels, L., Nijs, J., & Cagnie, B. (2015). Structural and functional brain abnormalities in chronic low back pain: A systematic review. *Seminars in Arthritis*

and Rheumatism, *45*(2), 229–237. http://doi.org/10.1016/j.semarthrit.2015.05.002
Krishnan, A., Woo, C. W., Chang, L. J., Ruzic, L., Gu, X., López-Solà, M., … Wager, T. D. (2016). Somatic and vicarious pain are represented by dissociable multivariate brain patterns. *eLife*, *5*(JUN2016), 1–42. http://doi.org/10.7554/eLife.15166
Kwok, Y. H., Tuke, J., Nicotra, L. L., Grace, P. M., Rolan, P. E., & Hutchinson, M. R. (2013). TLR 2 and 4 responsiveness from isolated peripheral blood mononuclear cells from rats and humans as potential chronic pain biomarkers. *PloS One*, *8*(10), e77799. http://doi.org/10.1371/journal.pone.0077799
Lee, M., Manders, T. R., Eberle, S. E., Su, X. C., James, D., Yang, X. R., … Wang, X. J. (2015). Activation of Corticostriatal Circuitry Relieves Chronic Neuropathic Pain, *35*(13), 5247–5259. http://doi.org/10.1523/JNEUROSCI.3494-14.2015
Lin, Y., & Su, Z. (2012). Balancing continuous and categorical baseline covariates in sequential clinical trials using the area between empirical cumulative distribution functions. *Statistics in Medicine*, *31*(18), 1961–1971. http://doi.org/10.1002/sim.5363
Lumley, M. A., Cohen, J. L., Borszcz, G. S., Cano, A., Radcliffe, A. M., Porter, L. S., … Keefe, F. J. (2011). Pain and emotion: A biopsychosocial review of recent research. *Journal of Clinical Psychology*, *67*(9), 942–968. http://doi.org/10.1002/jclp.20816
Manchikanti, L., Falco, F. J. E. E., Hirsch, J. a., Pampati, V., Falco, F. J. E. E., & Hirsch, J. a. (2012). Growth of Spinal Interventional Pain Management Techniques. *Spine*, *38*(2), 1. http://doi.org/10.1097/BRS.0b013e318267f463
Miller, E. M., & McDade, Th. W. (2012). A highly sensitive immunoassay for interleukin-6 in dried blood spots. *American Journal of Human Biology*, *24*(6), 863–865. http://doi.org/10.1002/ajhb.22324.A
Miller, F. G., & Kaptchuk, T. J. (2008). The power of context: reconceptualizing the placebo effect. *Journal of the Royal Society of Medicine*, *101*(5), 222–5. http://doi.org/10.1258/jrsm.2008.070466
Moore, K. M., & Martin, M. E. (2015). Using MBCT in a Chronic Pain Setting: A Qualitative Analysis of Participants' Experiences. *Mindfulness*, *6*(5), 1129–1136. http://doi.org/10.1007/s12671-014-0363-6
Moore, R. C., Depp, C. A., Wetherell, J. L., & Lenze, E. J. (2016). Ecological momentary assessment versus standard assessment instruments for measuring mindfulness, depressed mood, and anxiety among older adults. *Journal of Psychiatric Research*, *75*, 116–123. http://doi.org/10.1016/j.jpsychires.2016.01.011
Müller, M., Kamping, S., Benrath, J., Skowronek, H., Schmitz, J., Klinger, R., & Flor, H. (2016). Treatment history and placebo responses to experimental and clinical pain in chronic pain patients. *European Journal of Pain*, 1–12. http://doi.org/10.1002/ejp.877
Nummenmaa, L., Glerean, E., Hari, R., & Hietanen, J. K. (2013). Bodily maps of emotions. *Proceedings of the National Academy of Sciences of the United States of America*. http://doi.org/10.1073/pnas.1321664111
Ortiz, R., Chandros Hull, S., & Colloca, L. (2016). Patient attitudes about the clinical use of placebo: qualitative perspectives from a telephone survey. *BMJ Open*, *6*(4), e011012. http://doi.org/10.1136/bmjopen-2015-011012
Pedersen, L. M., Schistad, E., Jacobsen, L. M., Røe, C., & Gjerstad, J. (2015). Serum levels of the pro-inflammatory interleukins 6 (IL-6) and -8 (IL-8) in patients with lumbar radicular pain due to disc herniation: A 12-month prospective study. *Brain, Behavior, and Immunity*, *46*, 132–136. http://doi.org/10.1016/j.bbi.2015.01.008

Pek, J., & Flora, D. B. (2018). Reporting effect sizes in original psychological research: A discussion and tutorial. *Psychological Methods*, *23*(2), 208–225. http://doi.org/10.1037/met0000126

Ploner, M., Sorg, C., & Gross, J. (2016). Brain Rhythms of Pain. *Trends in Cognitive Sciences*, *21*(2), 100–110. http://doi.org/10.1016/j.tics.2016.12.001

Prichep, L. S., Shah, J., Merkin, H., & Hiesiger, E. M. (2017). Exploration of the Pathophysiology of Chronic Pain Using Quantitative EEG Source Localization. *Clinical EEG and Neuroscience*, 155005941773644. http://doi.org/10.1177/1550059417736444

Rosenthal, R., & Rosnow, R. (1985). *Contrast analysis: focused comparisons in the analysis of variance*. Cambridge: Cambridge University Press.

Roy, M., Shohamy, D., Daw, N., Jepma, M., Wimmer, G. E., & Wager, T. D. (2014). Representation of aversive prediction errors in the human periaqueductal gray. *Nature Neuroscience*, *17*(11), 1607–1612. http://doi.org/10.1038/nn.3832

Schafer, S. M., Colloca, L., & Wager, T. D. (2015). Conditioned placebo analgesia persists when subjects know they are receiving a placebo. *Journal of Pain*, *16*(5), 412–420. http://doi.org/10.1016/j.jpain.2014.12.008

Schistad, E. I., Espeland, A., Pedersen, L. M., Sandvik, L., Gjerstad, J., & R??e, C. (2014). Association between baseline IL-6 and 1-year recovery in lumbar radicular pain. *European Journal of Pain (United Kingdom)*, *18*(10), 1394–1401. http://doi.org/10.1002/j.1532-2149.2014.502.x

Schwartz, N., Temkin, P., Jurado, S., Lim, B. K., Heifets, B. D., Polepalli, J. S., & Malenka, R. C. (2014). Chronic pain. Decreased motivation during chronic pain requires long-term depression in the nucleus accumbens. *Science (New York, N.Y.)*, *345*(6196), 535–542. http://doi.org/10.1126/science.1253994

Seminowicz, D. a, Wideman, T. H., Naso, L., Hatami-Khoroushahi, Z., Fallatah, S., Ware, M. a, … Stone, L. S. (2011). Effective treatment of chronic low back pain in humans reverses abnormal brain anatomy and function. *The Journal of Neuroscience : The Official Journal of the Society for Neuroscience*, *31*(20), 7540–7550. http://doi.org/10.1523/JNEUROSCI.5280-10.2011

Seymour, B., O'Doherty, J. P., Koltzenburg, M., Wiech, K., Frackowiak, R., Friston, K., & Dolan, R. (2005). Opponent appetitive-aversive neural processes underlie predictive learning of pain relief. *Nature Neuroscience*, *8*(9), 1234–40. http://doi.org/10.1038/nn1527

Shallcross, A. J., Gross, J. J., Visvanathan, P. D., Kumar, N., Palfrey, A., Ford, B. Q., … Mauss, I. B. (2015). Relapse prevention in major depressive disorder: Mindfulness-based cognitive therapy versus an active control condition. *Journal of Consulting and Clinical Psychology*, *83*(5), 964–975. http://doi.org/10.1037/ccp0000050

Shpaner, M., Kelly, C., Lieberman, G., Perelman, H., Davis, M., Keefe, F. J., & Naylor, M. R. (2014). Unlearning chronic pain: A randomized controlled trial to investigate changes in intrinsic brain connectivity following Cognitive Behavioral Therapy. *NeuroImage: Clinical*, *5*, 365–376. http://doi.org/10.1016/j.nicl.2014.07.008

Skogstrand, K., Thorsen, P., Nørgaard-Pedersen, B., Schendel, D. E., Sørensen, L. C., & Hougaard, D. M. (2005). Simultaneous measurement of 25 inflammatory markers and neurotrophins in neonatal dried blood spots by immunoassay with xMAP technology. *Clinical Chemistry*, *51*(10), 1854–1866. http://doi.org/10.1373/clinchem.2005.052241

Steiger, J. H. (2004). Beyond the F test: Effect size confidence intervals and tests of close fit in the analysis of variance and contrast analysis. *Psychological Methods*, *9*(2), 164–182. http://doi.org/10.1037/1082-989X.9.2.164

Tilburt, J., Emanuel, E., Kaptchuk, T., Curlin, F., & Miller, F. G. (2008). Prescribing "placebo treatments": results of national survey of US internists and rheumatologists. *BMJ: British Medical Journal*, 1–5.

http://doi.org/10.1136/bmj.a1938
Tuttle, A. H., Tohyama, S., Ramsay, T., Kimmelman, J., Schweinhardt, P., Bennett, G. J., & Mogil, J. S. (2015). *Increasing placebo responses over time in U.S. clinical trials of neuropathic pain*. *Pain* (Vol. Publish Ah). http://doi.org/10.1097/j.pain.0000000000000333
Vachon-Presseau, E., Tétreault, P., Petre, B., Huang, L., Berger, S. E., Torbey, S., … Apkarian, A. V. (2016). Corticolimbic anatomical characteristics predetermine risk for chronic pain. *Brain*, aww100. http://doi.org/10.1093/brain/aww100
Vlaev, I., Seymour, B., Chater, N., Winston, J. S., Yoshida, W., Wright, N., … Dolan, R. (2012). Health Psychology Prices Need No Preferences : Social Trends Determine Decisions in Experimental Markets for Pain Relief Prices Need No Preferences : Social Trends Determine Decisions in Experimental Markets for Pain Relief. http://doi.org/10.1037/a0030372
Vlaev, I., Seymour, B., Dolan, R. J., & Chater, N. (2009). The Price of Pain and the Value of Suffering. *Psychological Science*, *20*(3), 309–317.
Wager, T. D., & Atlas, L. Y. (2015). The neuroscience of placebo effects: Connecting context, learning and health. *Nature Reviews Neuroscience*, *16*(7), 403–418. http://doi.org/10.1038/nrn3976
Wager, T. D., Atlas, L. Y., Leotti, L. a, & Rilling, J. K. (2011). Predicting individual differences in placebo analgesia: contributions of brain activity during anticipation and pain experience. *The Journal of Neuroscience : The Official Journal of the Society for Neuroscience*, *31*(2), 439–452. http://doi.org/10.1523/JNEUROSCI.3420-10.2011
Wager, T. D., Atlas, L. Y., Lindquist, M. A., Roy, M., Woo, C.-W., & Kross, E. (2013). An fMRI-based neurologic signature of physical pain. *The New England Journal of Medicine*, *368*(15), 1388–97. http://doi.org/10.1056/NEJMoa1204471
Wager, T. D., Rilling, J. K., Smith, E. E., Sokolik, A., Casey, K. L., Davidson, R. J., … Cohen, J. D. (2004). Placebo-induced changes in FMRI in the anticipation and experience of pain. *Science (New York, N.Y.)*, *303*(2004), 1162–1167. http://doi.org/10.1126/science.1093065
Winston, J. S., Vlaev, I., Seymour, B., Chater, N., & Dolan, R. J. (2014). Relative Valuation of Pain in Human Orbitofrontal Cortex. *Journal of Neuroscience*, *34*(44), 14526–14535. http://doi.org/10.1523/JNEUROSCI.1706-14.2014
Xiao, L., Yank, V., & Ma, J. (2012). Algorithm for balancing both continuous and categorical covariates in randomized controlled trials. *Computer Methods and Programs in Biomedicine*, *108*(3), 1185–1190. http://doi.org/10.1016/j.cmpb.2012.06.001
