# Supplementary material for: Effect of Pain Reprocessing Therapy vs Placebo and Usual Care for Patients With Chronic Back Pain: A Randomized Clinical Trial
Source: JAMA Psychiatry. 2021 Sep 29;79(1):1–12. doi: 10.1001/jamapsychiatry.2021.2669 (PMC8482298; doi:10.1001/jamapsychiatry.2021.2669)
Supplement: Supplement 2. — eMethods eResults eDiscussion eTable 1. Spinal anomalies among participants randomized to PRT eTable 2. Treatment response rates eTable 3. Secondary clinical outcomes measured only at pretreatment and posttreatment eTable 4. Treatment satisfaction and patient global impression of change eTable 5. Mediation results eTable 6. Values for mediators at each timepoint eTable 7. Evoked back pain localizer results eTable 8. Regions showing pretreatment to posttreatment connectivity changes for PRT vs placebo or PRT vs usual care eFigure 1. Evoked back pain localizer eFigure 2. Target masks for seed connectivity analyses eFigure 3. Individual trajectories of pain intensity for participants in the PRT, placebo and usual care groups eFigure 4. Effects of PRT on pain-related fear and avoidance and beliefs that pain indicates injury eFigure 5. Evoked back pain at pretreatment eFigure 6. High vs low thumb pressure stimulation eFigure 7. Histogram of quality control-functional connectivity correlations for spontaneous pain scans eFigure 8. Continuous pain regressors for 4 randomly chosen sample individuals eAppendix 1. Initial medical pain assessment and education session eAppendix 2. Pain reprocessing therapy description eAppendix 3. PRT treatment fidelity checklist eReferences [file jamapsychiatry-e212669-s002.pdf]

Supplemental Online Content

Ashar YK, Gordon A, Schubiner H, et al. Effect of pain reprocessing therapy vs placebo and usual care for patients with chronic back pain: a randomized clinical trial. *JAMA Psychiatry*. Published online September 29, 2021.
doi:10.1001/jamapsychiatry.2021.2669

eMethods
eResults
eDiscussion
eTable 1. Spinal anomalies among participants randomized to PRT
eTable 2. Treatment response rates
eTable 3. Secondary clinical outcomes measured only at pretreatment and posttreatment
eTable 4. Treatment satisfaction and patient global impression of change
eTable 5. Mediation results
eTable 6. Values for mediators at each timepoint
eTable 7. Evoked back pain localizer results
eTable 8. Regions showing pretreatment to posttreatment connectivity changes for PRT vs placebo or PRT vs usual care
eFigure 1. Evoked back pain localizer
eFigure 2. Target masks for seed connectivity analyses
eFigure 3. Individual trajectories of pain intensity for participants in the PRT, placebo, and usual care groups
eFigure 4. Effects of PRT on pain-related fear and avoidance and beliefs that pain indicates injury
eFigure 5. Evoked back pain at pretreatment
eFigure 6. High vs low thumb pressure stimulation
eFigure 7. Histogram of quality control-functional connectivity correlations for spontaneous pain scans
eFigure 8. Continuous pain regressors for 4 randomly chosen sample individuals
eAppendix 1. Initial medical pain assessment and education session
eAppendix 2. Pain reprocessing therapy description
eAppendix 3. PRT treatment fidelity checklist
eReferences

This supplemental material has been provided by the authors to give readers additional information about their work.

© 2021 Ashar YK et al. *JAMA Psychiatry.*

eMethods

Trial Design. Our randomization strategy created three conditions into which participants had equal probability of being randomized, as specified in the trial registration (clinicaltrials.gov NCT# 03294148). Rather than simple randomization to one of three arms, we designed the randomization strategy as a two-stage process. The pool of screened participants was first randomized to a PRT vs. usual care trial or to a placebo vs. usual care trial with a 1:1 ratio, and subsequently randomized again to one of the two conditions in each trial with a 2:1 ratio of PRT or placebo to usual care. The end result was 3 groups randomized to PRT, placebo, and usual care with equal probability.

We adopted this procedure, rather than a simple 3-way randomization, for two reasons. First, we anticipated comparing PRT vs. usual care and placebo vs. usual care in separate papers, due to their differing theoretical focus, or comparing all groups in an unbiased fashion if needed (as in the present manuscript). Second, with a traditional 3-way design, we believed that some participants might have been disappointed to be randomized to the open-label placebo injection rather than PRT, potentially attenuating placebo effects. Previous research has shown that the effects of open-label placebo treatments are sensitive to how they are presented to participants.[1,2] With this strategy, the placebo control provided (if anything) a stronger control condition with potentially greater pain relief.

All participants were exposed to identical recruitment materials and identical assessment procedures, and enrollment across the three condition was simultaneous and overlapping. In the analysis phase, we combined data from the usual care participants in both trials. Participants were paid for assessments, and treatment was provided at no charge. There were no major changes to protocols after study commencement.

Study Procedures. Participants first completed an online eligibility pre-screen. Potentially eligible participants were then randomized to trial (PRT vs. usual care or placebo vs. usual care). Participants then completed an in-person eligibility and consent session followed by a separate baseline fMRI session approximately 1 – 2 weeks later. Following the baseline fMRI session, a second randomization occurred within each trial

© 2021 Ashar YK et al. *JAMA Psychiatry.*

to treatment (PRT or placebo) vs. usual care at a 2:1 ratio, yielding a balanced sample size of n = 50 or 51 for each of the three groups.

Participants. Participants were recruited from the community using electronic and print announcements, social media, and referrals. Recruitment materials described a "mind-body treatment" for CBP.

Exclusion criteria, determined by self-report on the online pre-screen, targeted participants with primary (centralized) CBP. We excluded people with self-reported physician-diagnosed inflammatory disorders, a history of metastasizing cancer, unexplained unintended weight loss of 20 lbs. or more in the past year, and self-reported inability to control bowel or bladder function (a potential indicator of cauda equina syndrome), in addition to the inclusion/exclusion criteria reported in the main text. We also excluded participants unable or unlikely to comply with study procedures: people with self-reported diagnoses of schizophrenia, multiple personality disorder, or dissociative identity disorder; self-reported use of intravenous drugs; difficulty participating for technical/logistical issues (e.g., unable to get to assessment sessions or to complete remote surveys); pain-related compensation or litigation in the past year; and inability to undergo MRI (standard safety screen). People with self-reported history of stroke, brain surgery, or brain tumor were excluded due to difficulties normalizing such brains to standard templates. We also excluded a small number of participants who did not report increased pain during stimulation with a back pain evocation device (described below), because evoked pain was required for our planned fMRI analyses. Numbers excluded are provided in Figure 1.

PRT Treatment Fidelity Assessment. Treatment fidelity was assessed by independent raters coding audio recordings of two randomly selected sessions from half the patients receiving PRT. Coders indicated the presence or absence of six PRT components, as described in Appendix III. Treatment fidelity was high (see results presented in the main text).

Open-label Placebo. The placebo treatment was based on previous open-label placebo implementations.[3–6] Participants watched two videos communicating that a) placebos can powerfully reduce clinical pain, b) this can happen even when the treatment is known to be a placebo, due to "automatic engagement of the body's natural

healing responses", including brain, autonomic, and neuroendocrine pathways, and c) believing that the placebo will work is not necessary. Participants completed a warm, empathic clinical interview with author KK including patient history and rationale for the placebo treatment, with confirmation of the patient's understanding that the injection would be inert. Finally, the patient changed into a medical gown, lay prone on an examination table, and a subcutaneous saline injection was administered at the location of greatest pain.

Clinical Measures. We chose average pain intensity over the past week (measured with the Brief Pain Inventory-Short Form) as our primary outcome for several reasons: 1) It is highly interpretable to a broad audience,[7] 2) it is endorsed as a clinical outcome measure by both the IMPAACT committee for low back pain research and the creators of the BPI,[8–10] 3) it aligns with the "pain-free or nearly pain-free" measure of treatment response we defined, and 4) it correlated strongly with mean BPI-SF scores in our data (r ~= .90 at each timepoint).

Secondary outcomes measures included: the Oswestry Disability Index,[11] the 10-item version of the Positive and Negative Affect Scale (PANAS),[12] PROMIS short forms for depression (form 8a), anxiety (form 8a), sleep disturbance (form 8a), and anger (form 5a),[13,14] the Pain Catastrophizing Scale,[15] the 11-item version of the Tampa Scale of Kinesiophobia,[16,17] the two-item version of the Emotions subscale from the Survey of Pain Attitudes,[18] the timeline followback measure of self-reported use of alcohol, cannabis, and opioids in the previous two weeks,[19] the Patient Global Impression of Change scale (PGIC), and the Treatment Satisfaction Questionnaire.[20]

Three measures of pain-related beliefs were tested as potential treatment mechanisms in mediation analyses: a) Tampa Scale of Kinesiophobia (TSK-11), assessing belief that pain indicates injury and fear of movement, b) Pain Catastrophizing Scale (PCS), assessing pain magnification, rumination, and helplessness; and c) Survey of Pain Attitudes Emotion subscale (SOPA-Emotion), assessing the belief that pain is increased by stress and difficult emotions. We hypothesized that the TSK-11 would be most strongly related to treatment effects, as a central aim of PRT is reducing fear that pain indicates injury.

© 2021 Ashar YK et al. *JAMA Psychiatry.*

To reduce participant burden and increase the likelihood of obtaining data, only the following secondary outcomes were collected at follow-up: the ODI, four PROMIS measures, TSK-11, SOPA-Emotion, PGIC, and treatment satisfaction. The PANAS and the measures of alcohol, cannabis, and opioid use were only measured at pre- and post-treatment.

All measures were collected using the REDCap data collection system. At the pre- and post-treatment assessment sessions, participants completed these measures in a behavioral testing room with no investigators present. Follow up measures were collected remotely using REDCap survey links sent via email or SMS. To increase measurement reliability, baseline measures were defined as the average score from the two pre-randomization timepoints: the in-person eligibility and consent session and the pre-treatment session. Participants were required to report only >= 4/10 1-week-average pain intensity at the eligibility/consent session, so some participants had averaged baseline pain scores < 4/10.

Sample size and power analyses. Power analysis determined sample size using a meta-analytic estimate of Cohen's *d* = .62 for CBP pain intensity for psychological treatment vs. treatment-as-usual.[21] Effects of this size require n = 43 per group to achieve 80% power at α = .05. We aimed to enroll 50 per group, accounting for anticipated attrition, with n = 50 or 51 patients ultimately randomized to each group.

Randomization. Patients were randomized to study and then to PRT vs. usual care or placebo vs. usual care using an imbalance-minimization (matching) algorithm,[22] which balanced groups in number and on four covariates: pain intensity, age, gender, and opioid use (yes/no). Minimization algorithms are recommended when there are more than 2 covariates and/or continuous covariates, as was the case in our trial.[22–24] Randomization and patient notification of group assignment was performed by YA, who had no patient contact during data collection, and group assignment was concealed from research assistants conducting data collection.

Moderation of treatment response by age and gender. Moderation of treatment response by age and gender was tested by regressing pre-to-post-treatment changes in pain intensity on the group *x* age and group *x* gender interaction.

Computation of effect sizes. Treatment effect sizes were computed as the PRT vs. control difference in change from baseline to the given post-treatment or follow-up timepoint, divided by the pooled standard deviation of change scores, applying the Hedge's *g* correction and bootstrapped confidence intervals (10,000 iterations, computed with the *mes* toolbox).[25]

Correlational and mediation analyses. To examine potential treatment mechanisms, we conducted analyses of both co-occurring and time-lagged relationships between pain intensity and patient-reported pain beliefs. We hypothesized bidirectional influences between pain intensity and pain beliefs: pain beliefs can shift pain experience and relevant behaviors, and pain reductions achieved using psychological techniques can increase beliefs that pain is modifiable.

To investigate co-occurring changes, we computed the correlations between pre-to-post-treatment changes in pain and pre-to-post-treatment changes in the three measures of pain beliefs (TSK-11, PCS, SOPA-Emotion) among participants randomized to PRT. To investigate time-lagged changes we conducted mediation analyses, as well as "reverse" mediation analyses, described in the main text. Positive "reverse mediation" findings would support the plausibility of bidirectional effects, though nonsignificant findings would not rule this possibility out.[26,27] We intended mediation analyses to provide statistical evidence on whether results are consistent with hypothesized mechanisms, but not as definitive evidence for causal interactions. Statistical significance of mediation was computed with 10,000 bootstrapped iterations, using the CanlabCore MATLAB© toolbox.

MRI acquisition parameters. Structural images were acquired using a single shot T1 MPRAGE sequence with repetition time = 2.4 s, echo time = 2.07 ms, flip angle = 8°, number of slices = 224, slice orientation = sagittal, voxel size = 0.8 mm isotropic, field of view = 256 × 256 mm^2, GRAPPA acceleration factor = 2; echo spacing = 7.6 ms; bandwidth = 240 Hz per pixel.

Functional images were acquired using a multiband gradient-echo EPI sequence with repetition time = 460 ms, multi-band acceleration factor = 8, echo time = 27.2 ms, flip angle = 44°, number of slices = 56, slice orientation = transversal, phase encoding =

© 2021 Ashar YK et al. *JAMA Psychiatry.*

posterior to anterior, voxel size = 2.7 mm isotropic, gap between slices = 0 mm, field of view = 220 × 220 mm^2, echo spacing = 0.49 ms, bandwidth = 3,048 Hz per pixel.

Durations of functional scans were 16 minutes for the evoked back pain scan, 8 minutes for the spontaneous pain scan, and 6 minutes for the thumb-pressure scan. The evoked back pain scan was missing for one subject at post-treatment due to technical issues.

fMRI tasks design. Functional scans included an evoked back pain task, a “spontaneous pain” scan, and a thumb pressure-pain task serving as a positive control task for data quality assessment (see fMRI data quality assessment, below).

The evoked back pain task utilized a novel device providing experimental control over back pain during fMRI. Participants lay on a pneumatically-controlled cylindrical balloon, with increasing inflation causing increasingly painful back distention. The inflatable cylindrical balloon was placed under participants’ lower back immediately superior to the iliac crest. Distance from the balloon to the lateral malleolus was measured at pre-treatment and the balloon was placed was in the same location at post-treatment. Each subject received 20 trials (37 sec duration) at one of four inflation levels, and patients rated post-trial pain on a visual analog scale (VAS; 0 = no pain, 100 = worst pain imaginable). The balloon was never fully deflated during this task to limit larger head motions. The order of inflation levels for each subject was randomly permuted but constrained to optimize design efficiency by avoiding correlation with low frequency signals: Trials of inflation level 1 or 2 were always followed by inflation level 3 or 4 and vice versa, and consecutive trials always had different inflation levels. We adopted an extensive set of strategies for mitigating and controlling for head motion, described below.

Following the evoked back pain task, participants completed a scan measuring spontaneously occurring pain (no stimulation). Participants fixated on a foveal crosshair and provided a VAS rating (7 sec) of current back pain intensity each minute. We regressed out the rating task and analyzed residual resting-state connectivity, as described below.

MRI preprocessing pipeline. Standard fMRI preprocessing procedures were used, implemented in *fMRIprep* 1.2.4[28] which is based on Nipype 1.1.6.[29] Anatomical

T1-weighted (T1w) images from both scanning sessions were corrected for intensity non-uniformity (INU) sing N4BiasFieldCorrection[30] (ANTs 2.2.0). A T1w-reference map was computed after registration of the two T1w images (after INU-correction) using mri_robust_template.[31] The T1w-reference was then skull-stripped using antsBrainExtraction.sh (ANTs 2.2.0), using OASIS as target template. Spatial normalization to the ICBM 152 Nonlinear Asymmetrical template version 2009c[32] was performed through nonlinear registration with antsRegistration, using brain-extracted versions of both the T1w volume and the template.

For the functional run, first a reference volume and its skull-stripped version were generated using a custom methodology of fMRIPrep. A deformation field to correct for susceptibility distortions was estimated based on two echo-planar imaging (EPI) references with opposing phase-encoding directions, using 3dQwarp[33] (AFNI 20160207). Based on the estimated susceptibility distortion, an unwarped BOLD reference was calculated for a more accurate co-registration with the anatomical reference. The BOLD reference was then co-registered to the T1w reference using flirt[34] (FSL 5.0.9) with the boundary-based registration cost-function.[35] Co-registration was configured with nine degrees of freedom to account for distortions remaining in the BOLD reference. Head-motion parameters were estimated with respect to the BOLD reference before any spatiotemporal filtering using mcflirt (FSL 5.0.9). The BOLD time-series were resampled onto their original, native space by applying a single, composite transform to correct for head-motion and susceptibility distortions. The BOLD time-series were resampled to MNI152NLin2009cAsym standard space, generating a preprocessed BOLD run in MNI152NLin2009cAsym space using antsApplyTransforms, configured with Lanczos interpolation to minimize the smoothing effects of other kernels.[36]

Denoising pipeline. For both the evoked and spontaneous pain scans, nuisance covariates included 24 head motion parameters and "spike" regressors identifying volumes with framewise displacement (FD) >= .25 mm (often considered a strict threshold[37]). Spike regression was optimized for fast-TR data by a) applying a [.1 Hz – .5 Hz] band-stop filter to head motion parameters prior to computing FD, and b) computing FD with respect to the volume collected 2.4 sec previously (5 volume

© 2021 Ashar YK et al. *JAMA Psychiatry.*

difference).[38,39] We additionally included spike regressors for the four volumes following an identified spike, since effects of head motion can influence subsequent volumes as well. These nuisance covariates were included in 1st level models for evoked pain analyses and were regressed out of the spontaneous pain scans prior to connectivity analyses.

Denoising for the evoked back pain task included two additional procedures to limit the influence of head motion and remove signal less likely to be of neuronal origin. We applied anatomical CompCor, which generates nuisance covariates derived from signal fluctuations in white matter and cerebrospinal fluid,[40] and we included nuisance covariates with signal timeseries extracted from an anterior and a posterior out-of-brain area, to further capture and remove artifactual signal fluctuations in the data.

Denoising for spontaneous pain connectivity analyses additionally included global signal regression and band-pass filtering [.1 – .01 Hz], to focus on signal fluctuations most likely to be of neuronal origin.[41] We also included a nuisance regressor modelling the pain rating task (boxcar regressor) in order to more closely resemble traditional resting state analyses, though prior work has found that intrinsic connectivity networks are strongly preserved during the task performance.[42]

Evoked pain localizer region of interest selection. We conducted an evoked back pain localizer task identifying regions demonstrating significant within-person correlations with evoked back pain intensity over time. The localizer was conducted within a mask of regions most robustly implicated in chronic pain and pain appraisal.[43–52] The mask included the medial prefrontal cortex (mPFC), posterior cingulate cortex / precuneus (PCC/PC), insula, cingulate, primary and secondary somatosensory cortices (S1 and S2), nucleus accumbens (NAc), and amygdala (mask shown in eFigure 1b). Region definitions were derived from functional parcellations: the mPFC and PCC/PC region definitions were from the medial default mode network mask (DMN subnetwork "A") in Yeo et al.[53], other cortical regions definitions were from the Glasser et al. multi-modal parcellation,[54] and subcortical region definitions were from a high-resolution subcortical atlas.[54]

We conducted the localizer within a mask for two reasons. First, localizers often identify regions with significant signal that are not of theoretical interest (e.g., a face

© 2021 Ashar YK et al. *JAMA Psychiatry.*

viewing task will also produce signal in brain areas not of theoretical interest in face processing). Second, with n = 50 per group, we had 80% power to detect only moderately sized group differences (d = .55), motivating the need to define a more focused analytic search space.

Evoked back pain localizer design. Relationships with evoked back pain were identified by constructing a continuous within-person estimate of evoked pain intensity based on post-trial pain ratings (continuous evoked pain model, described below). Continuous pain values were entered as a regressor in each subject's 1st-level model (*Z*-scored so that voxel parameter estimates would capture pain magnitude) along with nuisance covariates, to estimate an evoked back pain parametric map for each participant at pre-treatment. In second-level (across subject) analyses, we submitted the pre-treatment parametric pain maps to a voxelwise regression with covariates for age, gender, mean evoked pain, and mean CSF activation (mean-centered, to control for artifactual signal at the second level). We applied a threshold of FDR $q < .05$, $k = 10$ within the mask to identify regions more strongly associated with pain intensity (Fig S1c). This yielded a set of discrete regions tracking evoked pain intensity (Fig S1d), with two large clusters spanning multiple anatomical boundaries divided into subregions based on an *a priori* atlas.[55] Region-average activity was then submitted to tests of Group *x* Time interactions (see main text).

Model of continuous evoked pain. To limit the confounding of pain-related and rating-related neural signals, we collected only post-trial ratings (VAS, 7 sec), rather than continuous pain ratings. Since participants were in pain throughout the task, we developed an exponential decay model of continuous pain based on the post-trial ratings.

This model was validated on a separate data set collected on a subset of study participants (*n* = 58) who completed an evoked back pain task in a behavioral testing room during their eligibility session visit. The validation task was identical to the task administered during fMRI, except participants provided continuous pain ratings using a trackball rather than brief post-trial ratings as during fMRI.

We fit an exponential decay model to estimate continuous pain intensity between post-trial ratings. This modelled a more rapid change in pain at the beginning of each

© 2021 Ashar YK et al. *JAMA Psychiatry.*

trial followed by an asymptotic approach to the next sample point. The model fit the formula $f(x) = (b-a) * (1 – e^{\tau x}) + a$, with a = pain rating at trial start, b = pain rating at trial end, x = trial timepoints between samples, and τ = a time constant governing the exponential decay process. τ was fit for each trial using the MATLAB curve fitting toolbox, and the average τ value across all subjects' trials was used to assess model performance (R^2 with bootstrapped confidence intervals, 10,000 bootstrap samples, MATLAB *bootci* function).

In this validation task, reported and model-predicted continuous pain were strongly related, mean R^2 = .85, 95% CI = [.82 .87]. Exponential decay model fits for four sample subjects are shown in eFigure 8.

Scans were excluded from analyses if post-trial pain ratings were missing on >= 25% of trials (n = 12), almost no pain was reported (pain <= 5/100 on 90% of trials, n = 3), or there was insufficient variability in pain (range <= 10/100, n = 3), as pain could not be reliably modelled in these scans.

Treatment effects on evoked pain. We tested for treatment effects in the regions identified by the back pain localizer using a mixed-effects ("random effects") model including two Group by Time interactions (PRT vs. placebo *x* Post vs. Pre, PRT vs. usual care *x* Post vs. Pre), covariates for age and gender, and a random intercept per subject (*fitlme*, MATLAB 2020a). We tested the directional hypothesis that treatment would reduce region-average activity in regions positively associated with back pain intensity (one-tailed test).

We followed this with an exploratory whole-brain test for treatment effects on evoked pain-related brain function (FSL randomise permutation test $p < .05$ with threshold-free cluster-enhancement and 10,000 iterations, to detect clusters exhibiting a significant Group by Time interaction), powered only to detect larger effects. No results survived whole-brain correction.

Spontaneous pain connectivity analyses. Spontaneous pain analyses tested seed-based connectivity with areas exhibiting a significant treatment effect in evoked pain analyses (anterior midcingulate, anterior prefrontal, and left anterior insula cortical regions; see results in main text). We tested for a Group *x* Time interaction in connectivity to two areas often found to have altered connectivity in chronic pain: (a)

midline regions of the default mode network (DMN), including the medial prefrontal and posterior cingulate cortex, and (b) primary somatosensory cortex (S1).[50,56–61] We estimated Pearson correlations between the average seed timeseries and target regions, computed a [Post – Pre] change score for each voxel, and conducted permutation tests of PRT vs. placebo and PRT vs. usual care group differences in connectivity change scores, with age and gender as covariates. Permutation tests were conducted with FSL randomise, with threshold-free cluster-enhancement (TFCE) and 10,000 permutations, applying a threshold of $p < .05$. Permutation tests were conducted within the DMN and S1 masks separately. The medial default mode network mask was taken from Yeo et al.[53] (DMN subnetwork "A"), and the S1 definition was taken from Glasser et al.,[54] (Brodmann Areas 1, 2, and 3).

This was followed by an exploratory permutation test conducted in a whole-brain mask. No clusters survived correction in the whole-brain mask.

fMRI data quality assessment. For the evoked back pain task, we assessed the influence of head motion at both the within-subject and between-subject level. Within-subject, we computed the variance inflation factor (VIF) for the pain regressor relative to the 24 head motion parameters, providing an estimate of task-correlated head motion. At the between-subject level, we included a head motion summary statistic (number of volumes identified as motion outliers) as a covariate in the mixed effects model and we tested for PRT vs. control differences in head motion.

For resting connectivity analyses with the spontaneous pain scans, we assessed data quality with "quality control-functional connectivity" ("QC-FC") correlations, using a whole-brain parcellation that included 489 parcels.[54,55] We computed the distribution of correlations between connectivity estimates and head motion across edges, an established measure of head motion associations with connectivity estimates.[37,41,62] We also tested for PRT vs. control group differences in head motion and repeated the 2nd level models with a head motion covariate. The median edge connectivity value was computed, and subjects' >= 3 standard deviations above the mean were excluded. Additionally, subjects were excluded if the spatial standard deviation of their connectivity map was >= 3 standard deviations above the group mean.

We also used a positive control task for data quality assessment. We administered 20 thumb pressure stimulations at a high and low pressure (4 and 7 kg/cm^2), estimated the [high – low] contrast for each subject, and applied an FDR $q < .05$ threshold to the group average contrast map using standard general linear model analyses in SPM12. We expected significant associations with the brain responses reliably reported in acute pain tasks (e.g., midcingulate, thalamus, insula, somatosensory cortex, cerebellum).

eResults

Treatment satisfaction and overall impressions of change. Satisfaction with PRT was high at post-treatment, M (SD) = 92.4 (8.01) out of 100, and remained high at 1-year follow-up, M (SD) = 85.28 (21.06), with similar results observed for patients' overall impressions of change (PGIC) (eTable 4).

Clinician evaluation results. Of the 45 patients who were randomized to PRT and completed the initial physician evaluation/education session, 43 were assessed as likely having centralized pain by PRT clinicians. Two had undetermined/unclear assessment findings at the initial session, with continued assessment during treatment indicating the likelihood of a substantial centralized contribution to pain. Of the 20 PRT patients with pre-existing radiological imaging, all had at least one spinal anomaly (median of 4 findings per patient; eTable 1), assessed by PRT clinicians as unlikely to be causal of pain (see Appendix I for an overview of the assessment procedure).[63]

fMRI data quality assessment. The positive control task produced the expected activations in pain-responsive regions (eFigure 6). Spontaneous pain scan correlations between head motion and functional connectivity estimates ("QC-FC correlations") were low, $r = .02$ (SD = .19) across edges (eFigure 7).[37,41,62] There were no PRT vs. placebo or PRT vs. usual care differences in head motion at pre- or post-treatment, all $p > .2$. Four subjects were excluded from spontaneous pain connectivity analyses due to poor

© 2021 Ashar YK et al. *JAMA Psychiatry.*

data quality (median edge correlation or spatial standard deviation more than 3 standard deviations above the group mean).

In the evoked back pain task, subjects had M = 189.11 (218.70) volumes flagged as spikes (~12% of volumes). This relatively strict approach to identifying volumes potentially corrupted by head motion still provided M = 11.03 (1.67) min of data for analyses. There were no PRT vs. placebo or PRT vs. usual care group differences in head motion at pre- or post-treatment, all $p > .4$. The statistical significance of the Group by Time interaction for the aIns or aPFC regions reported was not changed by including a head motion covariate in the model. Within-subject assessment of task-correlated head motion found mean VIF = 2.9 (SD = 1.5). Only one subject had VIF >= 10, a commonly used threshold for high collinearity. There were no PRT vs. placebo or PRT vs. usual care differences in VIFs at either timepoint, all $p > .13$. Overall, this suggests a limited influence of head motion on evoked back pain estimates at both the within- and between-subject level.

eDiscussion

PRT in relation to other psychological treatments

PRT shares concepts and techniques with other psychological treatments, including Cognitive behavioral therapy (CBT), Acceptance and Commitment Therapy (ACT), mindfulness-based treatments, exposure therapy, pain neuroscience education, emotion-focused treatments, and other approaches. Here, we briefly discuss how PRT relates to some of these approaches, while recognizing substantial variability in specific protocols within a given modality, as well as substantial variability in how protocols are interpreted and implemented by different providers.

CBT protocols typically aim to improve functioning by reducing pain catastrophizing and teaching pain coping skills. Some CBT protocols present the brain as primarily modulating incoming nociceptive signals from the affected body site (e.g., providing "gate control", rather than as "constructing" pain), while others emphasize that pain is an output of the brain and can be modulated by fear, worry, and avoidance.[64–66] CBT protocols often aim to help patients adopt a more reasonable, balanced

© 2021 Ashar YK et al. *JAMA Psychiatry.*

perspective on the pain and its impact, and typically do not take a strong stance on whether or not peripheral pathophysiology contributes to the pain.

Several mindfulness-based treatments also target improved functioning rather than primarily targeting pain reduction (e.g., ACT,[67] Mindfulness-Based Stress Reduction,[68] and Mindfulness-Oriented Recovery Enhancement[69]). Like PRT, they generally teach patients to attend to basic sensory aspects of pain experience with a non-reactive awareness, rather than resisting, avoiding, or emotionally elaborating on pain experience. However, mindfulness-based protocols typically do not promote the *reconceptualization* of pain as a "false alarm" of tissue damage.

Pain Exposure Therapy (PET), like PRT, aims to challenge harm beliefs and action outcome expectancies using education and exposure-based techniques. Although PET emphasizes that pain is harmless (i.e., not injurious), it typically does not take a clear stance on the *causes* of pain, which may limit the effectiveness of exposure in aiding the reconceptualization of pain in some cases. Additionally, the focus in exposure-based therapy is on relearning the threat value of feared actions (e.g., bending, standing) rather than reappraising feared sensations. Both may be useful and important.

PRT provides patients with a causal model of pain as due to brain processes and *not* due to bodily injury. Viewing the pain as a brain-generated "false alarm," PRT emphasizes that the pain is reversible and the body is healthy. This conceptual framework is similar to several pain neuroscience education protocols (e.g., Explain Pain[70]). Yet, previous studies have found that pain neuroscience education alone (without guided exposure, cognitive restructuring, and somatic attention and awareness) typically has limited effects on reducing or preventing chronic pain.[71,72] To help patients integrate and act on a new conceptualization of pain provided during treatment, PRT uses a number of techniques, including: a) individualized medical and psychological assessment to provide the patient with evidence for centralized causes of pain (e.g., pain that shifts locations, pain triggered by particular people or places; Appendix I and II); b) the expectation that centralized pain can be substantially reduced or eliminated with psychological treatment; and c) the guided reappraisal of pain sensations as non-threatening using a combination of mindfulness-based, cognitive, emotional and

© 2021 Ashar YK et al. *JAMA Psychiatry.*

somatic techniques (see Appendix II). Future studies are needed to identify which components of PRT are specifically efficacious.

Mediation of pain experience by changes in pain beliefs and vice versa

We found evidence for both the hypothesized direction of mediation and for the "reverse" direction. In the hypothesized ("forward") direction, we found that treatment effects on pain intensity were mediated by reductions in fearful beliefs that pain indicates injury, consistent with hypothesized treatment mechanisms. In the "reverse" direction, we found that treatment effects on fearful pain beliefs were mediated by pain intensity reductions. This may be because pain reductions experienced with psychological treatment can increase beliefs that pain is modifiable, brain-generated, and less driven by bodily injury (since pain was reduced by a psychological treatment). Taken together, this suggests likely cyclical/bidirectional relationships between pain intensity, pain beliefs, and fear/avoidance.

Two considerations prevent us from further, stronger interpretations of the mediation results. First, we did not measure beliefs and pain experience with sufficient frequency (temporal resolution) to make strong inferences about directional interactions. Second, causal interpretations derived from measured variables are difficult to make with confidence because of the potential for un-modeled lagged effects and common causal influences. For example, it is not recommended to interpret the relative statistical strength of "forward" and "reverse" mediation effects, as these can be caused by variation in the magnitude and sources of measurement error across measured variables, and thus we do not do so here.[27]

Choice of placebo control condition

Placebo research over the past decade shows that there is not one placebo effect, but many, arising from multiple sources. There is no single gold-standard "placebo control" for psychological interventions in particular, as mechanisms related to psychological appraisal and expectation are thought to be common to both placebo and active psychological treatments, though engaged to different degrees. A reasonable consensus position is that a placebo control for psychological therapy ought to engage

© 2021 Ashar YK et al. *JAMA Psychiatry.*

well-recognized “common factors”, including (a) being followed and assessed by the research team during the course of the study, and (b) positive expectations and hope arising from being given expert medical attention and a potentially beneficial treatment.

An emerging approach is to use an open-label placebo, which is given with the patient’s knowledge that it is not an active drug. Open-label placebos are typically coupled with expert medical attention, education on the ways in which the mind can influence symptoms, and suggestions that therapeutic benefits are possible and may arise through the patient’s engagement in treatment. Open-label placebos are considered ethically preferable to deceptive placebo treatments, and a growing evidence base shows they produce effects comparable to traditional deceptive placebos for clinical pain. Three previous trials comparing open-label placebo and traditional (deceptive) placebo all found that both forms of placebo treatments were similarly efficacious for pain or other chronic symptoms when the proper framing of open-label placebo was provided, as we implemented here.[4,5,73] In the present study, placebo effects relative to usual care comparable to or larger than those found in other studies of clinical pain. For example, in a meta-analysis of clinical trials of chronic neuropathic pain, Tuttle et al. (2015) reported 18.3% pre-to-post-treatment pain reductions with placebo treatments (95% CI 15.2% - 21.4%).[74] The Hrobjartsson and Gotzsche (2010) meta-analyses of placebo effects in chronic pain clinical trials reported a pain intensity reduction of d = -0.28 for placebo vs. usual care (95% CI -0.36 -0.19);[75] and a third meta-analysis by Peerdeman et al. (2016) reported average expectancy effects (including suggestion, imagery, conditioning, and placebo) of g = -0.33 for chronic pain (95% CI -0.04 -0.62).[76] While a complete comparison of placebo vs. usual care will be presented in a future manuscript, the placebo effects observed here (g = 0.53 for pain, relative to usual care) were comparable to the meta-analytic effect size estimates provided above (see Table 2 for data).

Generalizability of clinical outcomes

We recruited a sample from the community, including but not limited to referrals from pain clinics. Baseline pain severity was required to be >= 4, and averaged 4.41 at the first pre-treatment timepoint. Because the majority of people with back pain have

low-to-moderate (non-severe) pain,[5] we believe our results are relevant to a large portion of people with back pain.

Four out of 10 average pain intensity is clinically relevant. Patients with this level of pain are typically candidates for invasive procedures. For example, a large multi-site trial testing vertebroplasty for chronic back pain required 3 out of 10 pain for surgery eligibility,[78] and a large steroid injection trial required 2 out of 12 knee pain intensity for eligibility.[79]

Baseline pain in our study also appears lower due to our analytic approach. We computed baseline pain as the average of two pre-treatment assessments, to increase measurement reliability. As nearly always observed in clinical trials, pain decreased from the first to the second pre-treatment assessment, lowering the average baseline pain score. Although this approach boosts reliability of pain measurement, it contributes to the appearance of lower pain intensity relative to other trials, which typically use only a single pre-treatment measurement.

Future trials will be needed to test PRT effects in more selected populations (e.g., high-pain/high-disability patients, tertiary care clinics). Future trials will also be needed to test the efficacy of PRT for other centralized ("nociplastic") pain conditions (e.g., tension headache, irritable bowel syndrome, fibromyalgia). We believe PRT is applicable to these populations. We do not believe PRT is appropriate for nociceptive or neuropathic pain conditions. Elements of PRT may help treat "mixed" pain conditions, when centralized (nociplastic) processes are important contributors to pain.[80]

Interpretation of fMRI findings

In addition to fMRI findings discussed in the main text, there are several features of the fMRI results that merit discussion. The role of the anterior prefrontal cortex (aPFC) and adjacent dorsolateral prefrontal cortex (dlPFC) in pain is complex. This area has been found both to respond to noxious stimuli and to inhibit pain.[81] In our data, this region was increasingly activated with increased evoked back pain across 4 levels of back distention (Fig 1, Fig S1), paralleling previous findings in other samples that evocation of chronic back pain engages the dlPFC.[82,83] The aPFC reductions we observed for PRT vs. placebo may thus reflect decreased pain intensity or reduced

engagement of pain inhibitory mechanisms and a decreased priority on pain control, consistent with the framework of PRT and other psychological treatments.

We also observed heightened anterior midcingulate-precuneus (PC) connectivity for PRT vs. usual care. The PC and adjacent posterior cingulate cortex (PCC) are regions in the medial 'Default Mode Network', which is thought to subserve meaning-making, self-referential thinking, and affective appraisals.[84,85] In pain, lower PC/PCC activity during noxious heat has been associated with higher dispositional mindfulness,[86] and fibromyalgia patients exhibit greater PC/PCC activity when reflecting on pain-catastrophizing statements relative to neutral statements.[87] This suggests a role for the PC/PCC in pain appraisal, and it is possible that the PC connectivity changes we observed here reflect altered pain appraisal following PRT, though further research is needed to better understand the role of functional connectivity changes in pain treatments.

© 2021 Ashar YK et al. *JAMA Psychiatry.*

eTable 1. Spinal anomalies among participants randomized to PRT

Radiological finding	*N*	%
Disc degenerative changes	15	75%
Disc herniation or rupture	7	35%
Spinal misalignment	14	70%
Osteoarthritic changes	13	65%
Neuroforaminal narrowing	9	45%
Central canal stenosis	9	45%

Note. Percentages are out of *N* = 20 patients with pre-existing radiological records available for review. There was a median of 4 findings per patient.

© 2021 Ashar YK et al. *JAMA Psychiatry.*

eTable 2. Treatment response rates

	30% pain reduction		50% pain reduction		Pain-free or nearly pain-free	
	Post-tx	1-year	Post-tx	1-year	Post-tx	1-year
PRT	78%	70%	70%	60%	66%	52%
Placebo	49%	49%	29%	35%	20%	27%
Usual care	38%	30%	16%	20%	10%	16%

Note. Treatment response rates for each group at post-treatment (post-tx) and at 1-year-follow-up (1-year). The three treatment response categories assessed were report of 30% pain reduction, 50% pain reduction, and 0 or 1 out of 10 pain, which was defined as pain-free or nearly pain-free.

© 2021 Ashar YK et al. *JAMA Psychiatry.*

eTable 3. Secondary clinical outcomes measured only at pretreatment and posttreatment

	PRT Mean (SD)	Placebo Mean (SD)	Usual care Mean (SD)	PRT vs. Placebo, *g*	PRT vs. Usual care, *g*
PANAS positive affect (5-25)					
Baseline	16.73 (3.36)	16.38 (3.67)	15.80 (3.25)	-	-
Post-tx	17.89 (3.38)	15.20 (5.60)	14.98 (3.50)	0.63**	0.59**
PANAS negative affect (5-25)					
Baseline	8.86 (2.62)	8.04 (2.15)	8.21 (2.55)	-	-
Post-tx	8.30 (3.04)	7.70 (2.44)	8.19 (2.75)	-0.11	-0.32
Alcohol use, # of drinks					
Baseline	11.29 (11.09)	13.61 (14.93)	8.80 (11.10)	-	-
Post-tx	11.63 (10.83)	12.88 (14.30)	8.02 (10.63)	0.08	-0.09
Opioid use, # of pills					
Baseline	1.14 (4.64)	0.31 (1.14)	1.84 (8.87)	-	-
Post-tx	1.29 (4.50)	0.36 (2.11)	1.77 (8.99)	-0.04	0.07
Cannabis use, grams					
Baseline	7.50 (29.80)	3.49 (6.45)	1.06 (2.26)	-	-
Post-tx	6.76 (25.32)	3.31 (6.72)	1.49 (3.20)	-0.08	-0.27

Note. These measures were not collected at follow-up timepoints to reduce burden on study participants. Effect sizes show the group difference in change from baseline (Group by Time interaction). Means, standard deviations (SD), and effect sizes (Hedge's *g*) include all available data at the given timepoint (and corresponding baseline data for effect size computation). *** = $p < .001$, ** = $p < .01$, * = $p < .05$, † = $p < .1$. PANAS = Positive and Negative Affect Scale; higher indicates stronger affect. Post-tx = post-treatment.

© 2021 Ashar YK et al. *JAMA Psychiatry.*

eTable 4. Treatment satisfaction and patient global impression of change

	PRT Mean (SD)	Placebo Mean (SD)	Usual care Mean (SD)	PRT vs. Placebo, *g*	PRT vs. Usual care, *g*
Treatment Satisfaction (0-100)					
Post-tx	92.40 (8.01)	57.61 (23.05)	36.86 (23.01)	2.00***	3.15***
At 1 month	90.95 (11.94)	53.22 (26.73)	43.38 (17.96)	1.76***	3.08***
At 2 month	89.72 (13.59)	50.03 (28.52)	44.01 (20.76)	1.78***	2.56***
At 3 month	89.84 (14.20)	51.01 (28.96)	39.82 (20.13)	1.66***	2.83***
At 6 months	88.33 (15.11)	54.66 (27.71)	38.36 (24.12)	1.53***	2.49***
At 12 months	85.28 (21.06)	51.08 (30.01)	37.94 (24.27)	1.32***	2.08***
PGIC (1-7)					
Post-tx	6.14 (0.88)	3.61 (1.62)	2.06 (1.45)	1.92***	3.34***
At 1 month	6.00 (1.43)	3.32 (1.67)	2.10 (1.27)	1.70***	2.85***
At 2 month	5.92 (1.34)	3.45 (1.82)	2.61 (1.67)	1.53***	2.16***
At 3 month	6.00 (1.28)	3.21 (1.73)	2.19 (1.35)	1.80***	2.86***
At 6 months	5.98 (1.31)	3.43 (1.79)	2.49 (1.68)	1.63***	2.31***
At 12 months	5.84 (1.54)	3.50 (1.90)	3.14 (1.97)	1.35***	1.54***

Note. These measured were collected only at post-treatment and follow-up (not at pre-treatment). Effect size estimates show the group difference at each time point, not change from baseline. PGIC = Patient Global Impression of Change, with higher indicating greater improvement. Treatment Satisfaction: 0 = not satisfied, 100 = very satisfied.

© 2021 Ashar YK et al. *JAMA Psychiatry.*

eTable 5. Mediation results

		Mediation path				
		_a_	_b_	_c′_	_c_	_ab_
PRT vs. Placebo → Pre-to-post-tx change in TSK → Pain at follow-up						
Pain at 1 month		-1.34***	0.31**	-0.32	-0.73***	-0.41**
Pain at 2 months		-1.35***	0.26*	-0.32	-0.68***	-0.36*
Pain at 3 months		-1.45***	0.28*	-0.33	-0.74***	-0.41*
Pain at 6 months		-1.42***	0.34**	-0.28	-0.75***	-0.48**
Pain at 12 months		-1.41***	0.29*	-0.40	-0.81***	-0.41*
PRT vs. Placebo → Pre-to-post-tx change in pain → TSK at follow-up						
TSK-11 at 1 month		-0.99***	0.14	-0.88***	-1.02***	-0.14
TSK-11 at 2 months		-0.94***	0.23*	-0.87***	-1.08***	-0.21*
TSK-11 at 3 months		-1.00***	0.20*	-0.97***	-1.16***	-0.19†
TSK-11 at 6 months		-0.95***	0.29***	-0.68**	-0.95***	-0.28***
TSK-11 at 12 months		0.93***	0.31**	-0.84***	-1.13***	-0.29***
PRT vs. Usual care → Pre-to-post-tx change in TSK → Pain at follow-up						
Pain at 1 month		-1.24***	0.20*	-0.78**	-1.02***	-0.25†
Pain at 2 months		-1.28***	0.26*	-0.51*	-0.84***	-0.33*
Pain at 3 months		-1.25***	0.23*	-0.75**	-1.04***	-0.30*
Pain at 6 months		-1.25***	0.12	-0.85***	-1.00***	-0.15
Pain at 12 months		-1.30***	0.23	-0.70†	-1.01***	-0.30*
PRT vs. Usual care → Pre-to-post-tx change in pain → TSK at follow-up						
TSK-11 at 1 month		-1.35***	0.26***	-0.62***	-0.98***	-0.36**
TSK-11 at 2 months		-1.41***	0.22*	-0.67**	-0.98***	-0.31*
TSK-11 at 3 months		-1.42***	0.22**	-0.82***	-1.12***	-0.31*
TSK-11 at 6 months		-1.43***	0.31***	-0.62**	-1.06***	-0.44***

© 2021 Ashar YK et al. *JAMA Psychiatry.*

TSK-11 at 12 months		-1.40***	0.48***	-0.22	-0.89***	-0.67***

Note. Standardized βs are shown, with asterisks to indicate statistical significance. For all models, the independent variable was treatment group assignment (PRT vs. placebo or PRT vs. usual care), dummy coded. The mediator variable was pre-to-post-treatment change, and the dependent variable was measured at the noted follow-up timepoint. We tested the Tampa Scale of Kinesiophobia 11-item form (TSK-11) as a mediator for treatment effects on 1-week average pain intensity, and the reverse—pain as a mediator of treatment effects on TSK-11. All analyses controlled for baseline levels of the dependent variable. The Pain Catastrophizing Scale and the Survey of Pain Attitudes Emotion subscale did not significant mediate treatment effects on pain intensity at any follow-up timepoint (results not shown). Estimates for path *a* varied slightly across timepoints due to different observations missing at different timepoints. Values of each mediator at each time point presented in eTable 6. † = $p < .1$, * = $p < .05$, ** = $p < .01$, *** = $p < .001$.

© 2021 Ashar YK et al. *JAMA Psychiatry.*

eTable 6. Values for mediators at each timepoint

	PRT Mean (SD)	Placebo Mean (SD)	Usual care Mean (SD)	PRT vs. Placebo, *g*	PRT vs. Usual care, *g*
Tampa Scale of Kinesiophobia (11-44)					
Baseline	23.62 (5.46)	23.10 (4.62)	23.93 (4.68)	-	-
Post-tx	16.41 (5.37)	22.16 (4.94)	22.51 (6.30)	-1.90***	-1.67***
At 1 month	16.16 (5.21)	21.45 (5.55)	22.33 (6.57)	-1.48***	-1.53***
At 2 month	16.03 (5.11)	21.15 (5.62)	22.45 (7.00)	-1.48***	-1.37***
At 3 month	15.14 (5.09)	21.58 (6.53)	22.62 (6.42)	-1.75***	-1.63***
At 6 months	16.61 (5.88)	21.91 (6.22)	23.49 (6.21)	-1.17***	-1.42***
At 12 months	17.16 (5.82)	23.33 (5.40)	21.75 (5.88)	-1.28***	-0.93***
Survey of Pain Attitudes – Emotion subscale (2 – 10)					
Baseline	7.52 (1.65)	7.23 (1.85)	7.53 (1.57)	-	-
Post-tx	8.68 (2.13)	7.20 (2.39)	6.96 (2.26)	0.64**	0.85***
At 1 month	8.55 (2.19)	6.89 (2.44)	6.72 (2.35)	0.84***	0.97***
At 2 month	8.03 (2.44)	6.85 (2.31)	7.21 (2.26)	0.47*	0.46*
At 3 month	8.61 (1.93)	6.61 (2.57)	6.95 (2.24)	1.04***	0.80***
At 6 months	8.39 (2.06)	6.71 (2.55)	7.27 (2.09)	0.63**	0.66**
At 12 months	8.27 (2.14)	7.05 (2.12)	7.17 (2.10)	0.52*	0.66**
Pain Catastrophizing Scale (from 0-52)					
Baseline	8.86 (2.62)	8.04 (2.15)	8.21 (2.55)	-	-
Post-tx	8.30 (3.04)	7.70 (2.44)	8.19 (2.75)	-0.11	-0.32

Note. The effect size shows the group difference in change from baseline to the indicated timepoint. The Pain Catastrophizing Scale was collected only at baseline and post-treatment to reduce participant burden.

© 2021 Ashar YK et al. *JAMA Psychiatry.*

eTable 7. Evoked back pain localizer results

Region	Volume	X	Y	Z	Max *Z*
Amygdala	3016	19	5	-13	4.46
Retrosplenial cortex	4080	3	-46	8	4.82
Precuneus L.	1008	-10	-49	46	3.27
Frontal operculum/mid-insula R	12136	46	18	0	5.75
Inf. frontal gyrus (BA 47l L)	1056	-50	37	-5	4.31
Lateral PFC (BA 9/46d L)	2368	-26	50	22	3.61
S1 R.	24896	35	-30	57	6.33
S1/S2 L	33000	-34	-27	60	7.03
S2 L.	13176	-49	-29	21	3.29
Precentral gyrus (BA4) R	2216	44	-14	38	3.13
S2 (BA PFcm) R	7720	46	-30	25	7.03
Frontal operculum/mid-insula L	12960	-45	5	3	4.65
Ant. Insula L	1072	-29	29	3	3.32
Precuneus/posterior cingulate	13312	1	-34	45	6.01
Anterior midcingulate (BA 32)	1624	-1	31	34	7.03
Midcingulate	15608	3	11	38	4.04

Note. Regions significantly positively related to evoked pain intensity within a mask of interest (see also eFigure 1). No negative effects survived correction. Region labels provided with reference to the Harvard-Oxford cortical atlas and a multi-modal atlas[55]. BA = Brodmann's Area; S1 = primary somatosensory cortex; S2 = secondary somatosensory cortex; PFC = prefrontal cortex; Ant. = anterior; Inf. = Inferior; L = left, R = right.

© 2021 Ashar YK et al. *JAMA Psychiatry.*

eTable 8. Regions showing pretreatment to posttreatment connectivity changes for PRT vs placebo or PRT vs usual care

	Region	Volume	X	Y	Z	Max Z	*p*
aIns seed, PRT vs. Placebo							
	BA2 R	928	46	-26	42	3.55	.04
aIns seed, PRT vs. Usual care							
	BA3b R	1048	40	-22	56	3.58	.03
aPFC seed, PRT vs. Placebo							
	BA3b L	1504	-56	-16	46	3.90	.02
	BA1 R	1256	54	-12	48	3.91	.03
aPFC seed, PRT vs. Usual care							
	No clusters survived correction						
aMCC seed, PRT vs. Placebo							
	No clusters survived correction						
aMCC seed, PRT vs. Usual care							
	Precuneus (BA 7)	3464	0	-64	36	4.23	.01

Note. Analyses tested Group (PRT vs. control) by Time (Post – Pre) interactions. Coordinates corresponding to results displayed in Figure 5d. BA = Brodmann's Area; L = left; R = right; aIns = anterior insula; aPFC = anterior prefrontal cortex; max *Z* and *p* values derived from permutation tests with threshold-free cluster-enhancement (TFCE) conducted within two masks—primary somatosensory cortex and medial default mode network (DMN) regions. No clusters survived correction within the DMN mask.

© 2021 Ashar YK et al. *JAMA Psychiatry.*

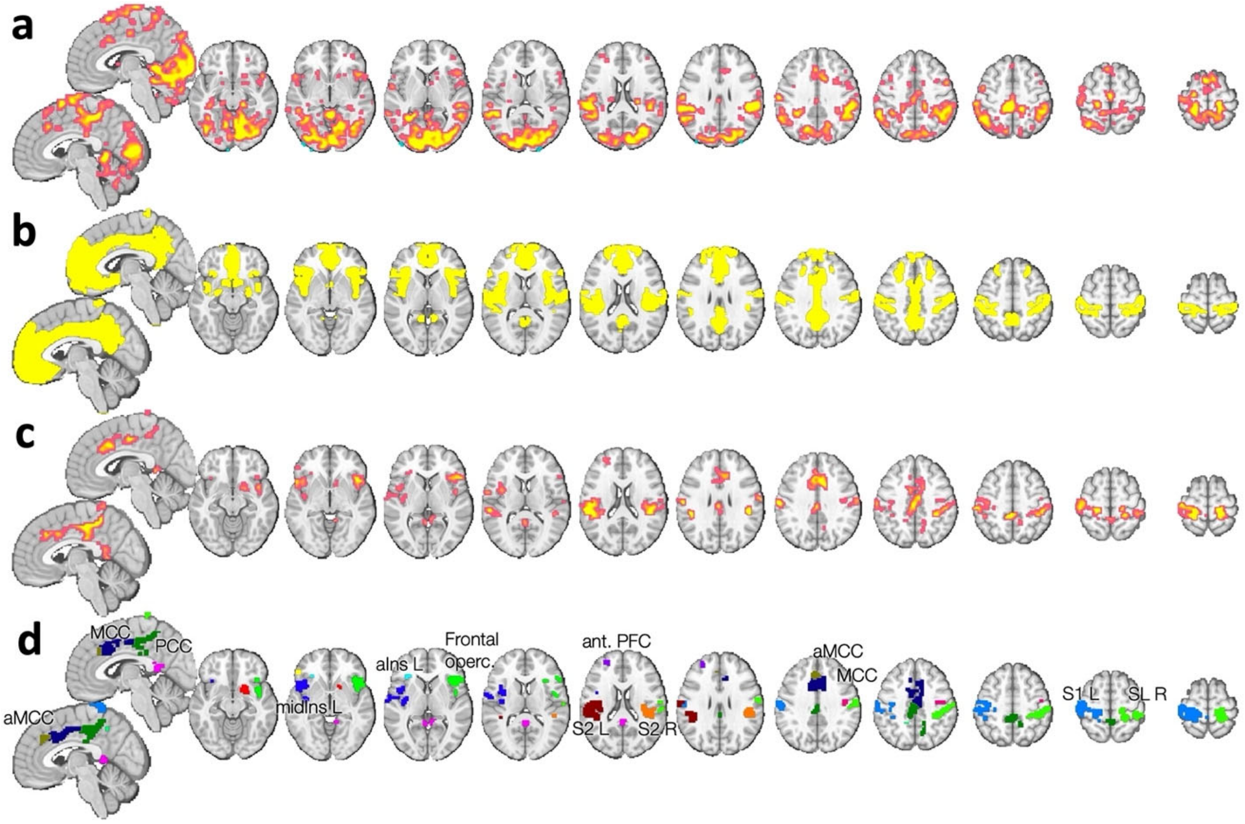

eFigure 1. Evoked back pain localizer. We conducted a localizer within a mask defining *a priori* regions of interest. This served to identify regions that were associated with evoked back pain intensity and were of theoretical interest, for testing of treatment effects. A) Whole-brain correlates of evoked back pain intensity, FDR $q < .05$, equivalent to uncorrected $p < .016$, owing to strong widespread signal. B) Mask defining regions of interest, including the medial prefrontal, cingulate, insular, and somatosensory cortices, precuneus, nucleus accumbens, and amygdala (see Supplementary Methods for details of mask construction). C) Correlates of evoked back pain intensity within the regions of interest (i.e., the intersection of (a) and (b)), with a threshold of FDR $q < .05$ applied, equivalent to uncorrected $p < .001$. D) Results from (c) were divided into discrete regions, with larger clusters divided along anatomical boundaries. Region-average responses to evoked pain were submitted to Group *x* Time interactions, testing for effects of treatment vs. control. MCC = midcingulate cortex; aMCC = anterior MCC; PCC = posterior cingulate cortex; midIns = mid-insula; FOP = frontal operculum; ant. PFC = anterior prefrontal cortex; S1 = primary somatosensory cortex; S2 = secondary somatosensory cortex; L = left; R = right.

© 2021 Ashar YK et al. *JAMA Psychiatry.*

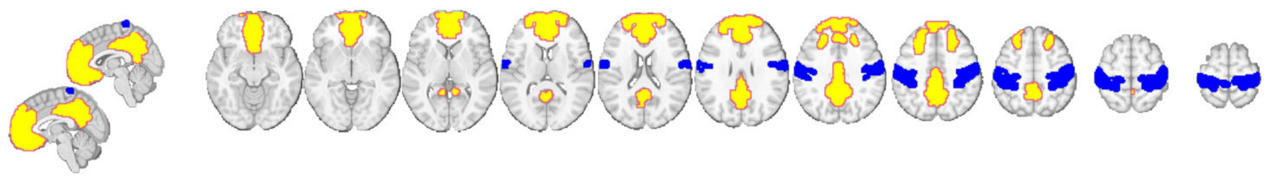

eFigure 2. Target masks for seed connectivity analyses. Yellow = medial default mode network regions, as defined in Yeo et al.[53]; blue = primary somatosensory cortex, as defined in Glasser et al.[55] (see Supplementary Methods for details).

© 2021 Ashar YK et al. *JAMA Psychiatry.*

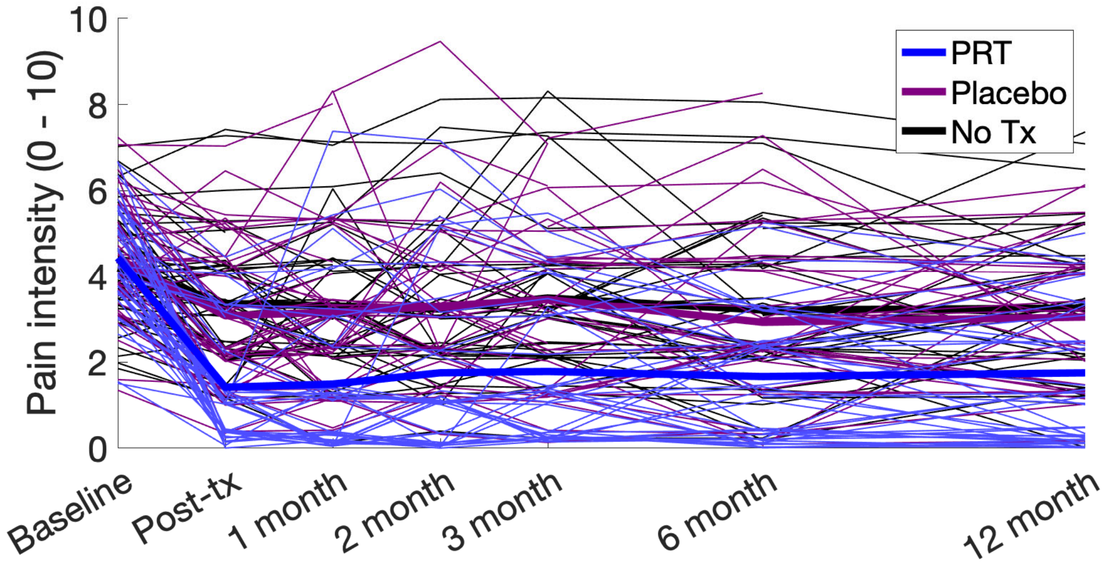

eFigure 3. Individual trajectories of pain intensity for participants in the PRT (blue), placebo (purple), and usual care (black) groups, jittered slightly for visualization purposes. Thick lines show group means. All available data is displayed.

© 2021 Ashar YK et al. *JAMA Psychiatry.*

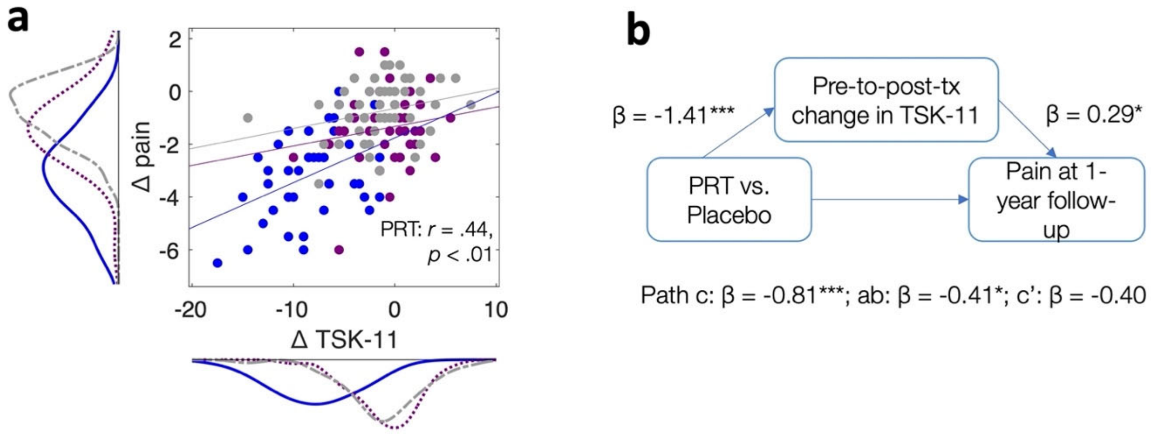

eFigure 4. Effects of PRT on pain-related fear and avoidance and beliefs that pain indicates injury, operationalized as Tampa Scale of Kinesiophobia (TSK-11) scale scores. a) In the PRT group, pre-to-post-treatment reductions in pain correlated with reduced TSK-11 scores; this correlation was not significant in the placebo or usual care groups. b) Pre-to-post-treatment reductions in the TSK-11 mediated treatment effects on pain intensity at multiple follow-up timepoints. Path coefficients for PRT vs. placebo at 1-year follow-up shown here; full mediation results presented in eTable 5. Pre-to-post-treatment pain reductions also mediated the effects of treatment on pain beliefs at follow-up ("reverse mediation"; see text for details).

© 2021 Ashar YK et al. *JAMA Psychiatry.*

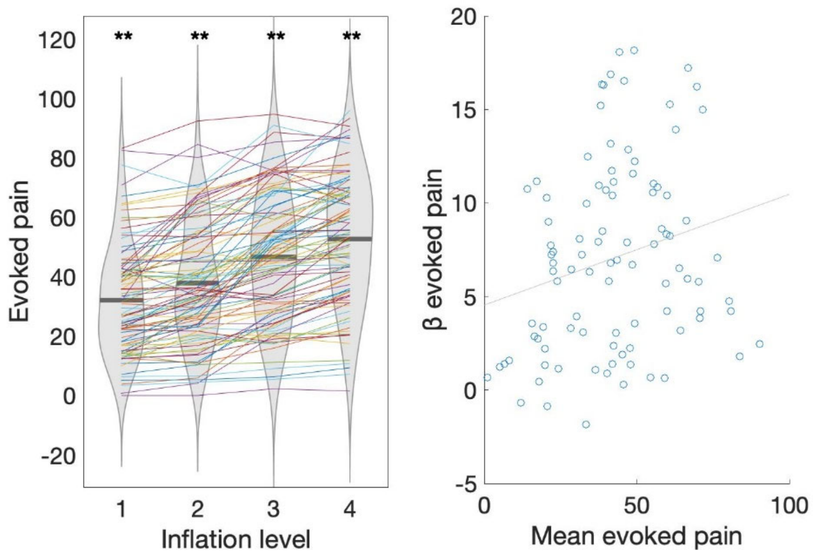

eFigure 5. Evoked back pain at pretreatment. The left panel shows mean evoked pain at each inflation level for each patient (colored lines). The right panel shows mean evoked pain by increase in pain per increase in inflation level (β evoked pain, estimated using linear regression) for each patient.

© 2021 Ashar YK et al. *JAMA Psychiatry.*

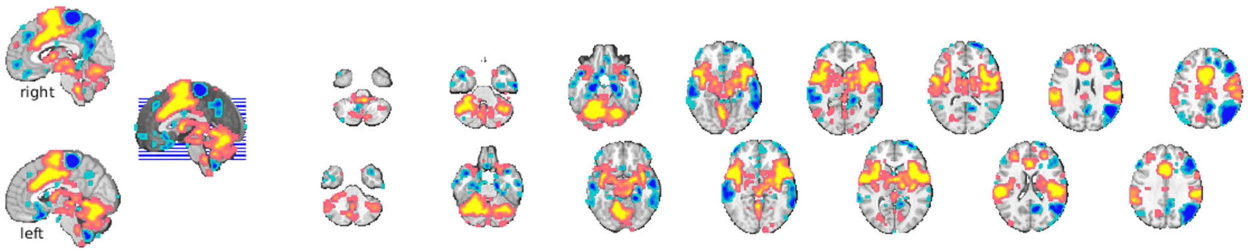

eFigure 6. High vs low thumb pressure stimulation, FDR $q < .05$, serving as a positive control. Effects are observed in the expected pain-responsive regions.

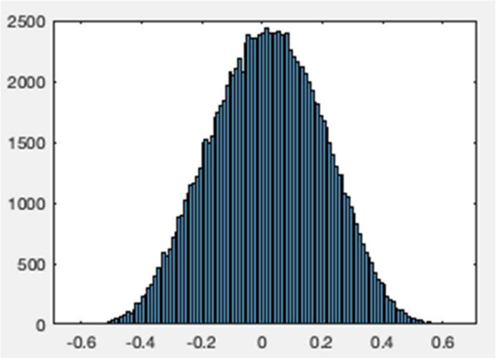

eFigure 7. Histogram of quality control-functional connectivity (QC-FC) correlations for spontaneous pain scans.

© 2021 Ashar YK et al. *JAMA Psychiatry.*

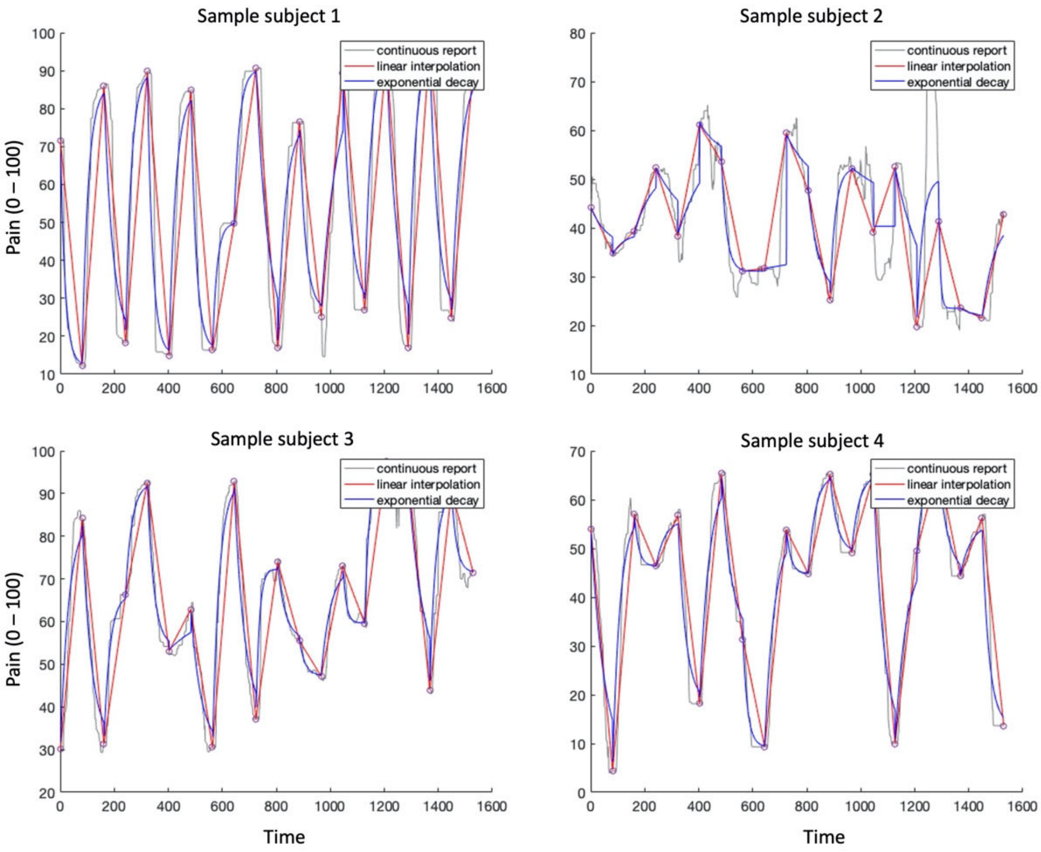

eFigure 8. Continuous pain regressors for 4 randomly chosen sample individuals. Grey line shows observed continuous report in the validation data, with gray circles indicating the samples taken at post-trial intervals. Predicted continuous pain between samples is shown for the linear interpolation (red) and exponential decay model (blue).

© 2021 Ashar YK et al. *JAMA Psychiatry.*

eAppendix 1. Initial medical pain assessment and education session

Each patient had an initial 1-hour telehealth visit with a physician with expertise in mind-body medicine, pain diagnosis, and patient education (author HS). This session assessed likely peripheral and centralized contributions to the patient's pain through detailed attention to pain history and presentation, stressful life events, and other contextual factors (see also an additional description[88] and the complete protocol[89]). There are four steps in this process: a) ruling out structural pain, b) education on predictive coding and neural circuit pain, c) ruling in neural circuit pain, and d) personalizing the information.

First, there was a "ruling out" component of the assessment to exclude a clearly identified structural problem such as a tumor, fracture, infection, or inflammatory condition. In addition, there was an assessment for any neurological deficit that can occur with severe spinal stenosis or a herniated disc. MRIs, when available, were reviewed. All participants with available MRIs had some radiological findings, including degenerative discs disease, bulging discs, spinal stenosis, spondylolisthesis, and facet arthropathy (see main text and eTable 7). However, because these abnormalities are seen in the majority of asymptomatic individuals, these were not assumed to be causing back pain.

Second, the patient was given education on the role of the brain in the generation and maintenance of pain. The model of predictive coding was explained so that patients understood that pain is generated by the brain and that pain can be triggered by either physical injury, predicted (but not actual) injury, or by stress and difficult emotions. It was emphasized that all pain is real and not due to the patient's imagination, and that no blame or stigma should be associated with having centralized chronic pain. Neural circuits in the brain generate all pain, and persistent centralized pain is driven by a cycle of pain leading to fear and focus on pain, which leads to increased pain.

Third, a series of detailed assessments were done to gather evidence that would "rule in" a centralized pain process. The assessed factors included:

1) having a history of other syndromes that fit the category of central sensitization syndromes, such as irritable bowel syndrome, fibromyalgia, tension headaches, anxiety, depression, and chronic fatigue;

2) review of a pain body map to determine if the patient has had pain in a variety of body regions;

3) determination of linkages between the onset of back pain and particularly stressful life events;

© 2021 Ashar YK et al. *JAMA Psychiatry.*

4) determination if the pain characteristics fit into criteria for a functional disorder, including pain that spreads to different areas over time, pain that is in a large region of the back, pain that radiates to areas unrelated to nerve root distributions, pain that has persisted after an injury would have healed, and pain that has newly arisen again in an area of an old injury;

5) determination if pain characteristics are inconsistent, such as pain that shifts to different regions of the back, pain that typically varies significantly at different time points of the day, pain that temporarily resolves when engaged in distracting or pleasant activities, pain that temporarily resolves after treatments that are likely to be placebos (e.g., energy work), pain that does not increase while engaging in certain physical activities but then dramatically increases after the activity; and

6) whether pain is triggered by innocuous stimuli, such as pain that worsens with exposure to certain smells, sounds, lights, computer screens, light touch; or pain that occurs on a significant anniversary, or in anticipation of a stressful event; or pain that occurs with imagining physical movements or stressful life events.

Findings were discussed with the patient, with great care taken to validate and destigmatize the patient's pain experience, using a framework including three heuristic categories: 1) structural pain, such as the case with tumors, infections, fractures; 2) centralized pain; and 3) a combination of the two. For participants assessed to likely have centralized pain, education was provided that: a) their pain was due to central nervous system processes and did not accurately indicate tissue damage, and b) 'centralized' pain can be greatly reduced or even eliminated with the upcoming treatment sessions.

All participants continued to PRT psychotherapy sessions, regardless of assessment findings. An important component of PRT is the continued process of gathering evidence for centralized pain (see Appendix II). In cases where the initial assessment was ambiguous, this process helped clarify the likely pain subtype.

© 2021 Ashar YK et al. *JAMA Psychiatry.*

eAppendix 2. Pain reprocessing therapy description

Pain Reprocessing Therapy (PRT) has five main components: 1) education about the brain origins and reversibility of pain, 2) gathering and reinforcing *personalized* evidence for the brain origins and reversibility of pain, 3) attending to and appraising pain sensations through a lens of safety, 4) addressing other emotional threats, and 5) gravitating to positive feelings and sensations. We provide here a brief overview of PRT, and a PRT manual is forthcoming (Gordon & Ziv, forthcoming).

I. Education about the brain origins and reversibility of pain and the pain-fear cycle.

PRT begins with education about how pain can be present in the absence of any tissue damage. For example:

> “Pain is a danger signal. If you put your hand on a hot stove, the pain is letting you know to move your hand, so you don’t injure yourself further. But sometimes these danger signals can get activated even in the absence of structural damage. Sometimes, the brain can interpret safe signals from the body as if they’re dangerous, even though there is nothing injured in the body. In these cases, the pain is like a “false alarm”. The alarm is really going off (your pain is totally real), and at the same time, there really is no fire (your body is not injured).”

Education is then provided about the pain-fear cycle. For example:

> “When we have a lot of fear and preoccupation around the pain, it reinforces to the brain that the pain is dangerous, and the pain persists. Here’s how the cycle works:
>
> 1. Pain triggers feelings of fear.
> 2. The fear puts the brain on high alert which causes more pain.
> 3. Which leads to more fear.
> 4. Which leads to more pain.
>
> We break this cycle by shifting our perspective of the pain and thinking of it as completely safe. As you learn to eliminate the fear around the pain, over time your pain will fade” (Gordon and Ziv, forthcoming).

II. Gathering and reinforcing evidence

It is difficult to overcome the fear around the pain if one believes that the pain is an accurate reflection of tissue damage in the body. So, a goal of treatment is to help

© 2021 Ashar YK et al. *JAMA Psychiatry.*

patients embrace the idea that *their* pain is due to central processes, as opposed to a structural or physical problem in their bodies.

This can be challenging for three reasons:

1. Biology: We are evolutionarily wired to associate physical pain with physical injury.
2. Previous diagnoses: Many chronic back pain patients have been given structural diagnoses (herniation, disc degeneration, etc.).
3. Learned associations: Many chronic back pain patients have developed learned associations – physical positions (e.g., sitting, standing) or activities (e.g., walking, running, bending) that have come to be associated with the onset pain, reinforcing the belief that there is something structurally wrong with them.

One way to combat the belief that there is a "structural"/peripheral cause of the pain is by gathering as much counter-evidence as possible – evidence reinforcing that the pain is actually due to central processes, as opposed to a structural problem in the body. Indicators of centralized pain include:

- Pain originating during a time of stress
- Pain originating without injury
- Inconsistencies in how pain presents
- Patient presenting with a variety of different somatic symptoms
- Pain triggers that indicate centralized processes (e.g., social contexts, etc.)
- Instances where the pain wasn't present, despite patient engaging in physical positions or activities that generally brings it on

The therapist and patient work together in a collaborative effort to gather and reinforce evidence that their pain is not a function of underlying structural pathology. The therapist can assist the patient in developing an evidence sheet - a list of all the support that reinforces that patient's pain is due to central processes. A sample evidence sheet might be:

- MRI showed that my back looked pretty good overall.
- I have a history of other pain syndromes (headaches, IBS…)
- My back pain started two weeks after my mom moved in with me.
- The pain is a lot worse when I'm at work and it's barely there over the weekend.

This process of evidence gathering is ongoing. Often treatment can provide additional evidence for centralized pain, which can create a positive feedback loop. For example, if the therapist leads the patient in a psychological exercise that results in a large pain

© 2021 Ashar YK et al. *JAMA Psychiatry.*

reduction (e.g., during "somatic tracking"—see below), this becomes another piece of evidence that there may not be a structural basis for the pain.

III. Attending to and appraising pain sensations through a lens of safety

A central technique in PRT is called "somatic tracking". The goal of somatic tracking is to help the patient attend to pain sensations through a lens of safety. Somatic tracking is used both during interoceptive exposures to pain sensations and during situational exposures to feared, pain-eliciting activities.

When the patient has pain associated with a physical position like sitting, the therapist can guide the patient in a somatic tracking exercise while the patient is seated. When the patient has pain associated with movements or activities (walking, bending, twisting, etc.), the therapist can lead the patient in a somatic tracking exercise while the patient is engaging in that movement or activity.

Somatic tracking involves three components: mindfulness, safety reappraisal, and positive affect induction. The **mindfulness** component of somatic tracking promotes exploring the pain sensations with a sense of objective interest and curiosity:

- "How would you describe the quality of the sensation?"
- "Is it widespread or localized?"
- "You don't need to change it, you don't need to get rid of it, you're just exploring it. It's like you're a passenger in the car, just along for the ride."

Mindfulness alone often isn't sufficient to neutralize the fear around the pain, motivating the need for the second component of somatic tracking: **safety reappraisal**. During a somatic tracking exercise, the therapist continuously helps the patient reappraise the sensation as safe:

- "Even though it's a tight/burning/tingling sensation, we know that it's safe. We've gathered a lot of evidence. Your back is perfectly healthy. Your brain is simply misinterpreting the signals coming from your body as if they're dangerous."
- "We all feel sensations in our backs when we bend. Because our muscles are being stretched. In fact, it's often a nice sensation. It's just that your brain is interpreting this sensation through a lens of danger, so it's being experienced as unpleasant. But there's nothing wrong with your back. Your muscles, your tendons, your ligaments, they're all perfectly healthy. This is a safe sensation. It's just a gentle stretch."
- "Right now, you're feeling a burning sensation in your back. But that isn't the issue. The issue is that you think burning indicates danger. But burning doesn't have to feel bad. Think about when you first get into a jacuzzi, or when you're

taking a nice, hot shower… there's a burning sensation, but it actually feels really nice. So, see if you can pay attention to this burning sensation in your back right now. We know that there's nothing wrong with your body, this is just your brain putting on a show for you. It's just an interesting burning sensation, but we know that it's safe. So just sit back and enjoy the show."

The safety reappraisal component of somatic tracking is important, but if the patient isn't able to authentically buy into these messages of safety, it falls flat. This is why the evidence gathering component of PRT is so important. If the patient can truly embrace that the pain isn't a reflection of tissue damage in their body, it lays the foundation to authentically attend to these sensations through a lens of safety.
The third component of somatic tracking is **positive affect induction**. If the therapist is able to lighten the mood, it allows the patient to more easily attend to the sensation through a lens of safety and positivity. Humor is one of the best ways to achieve positive affect induction:

- "Remember, whatever happens to the sensation is okay. Because it's safe. So, let it do what it's going to do. All you have to do is watch. It's like you're snorkeling or scuba diving and you're floating there, and you see a school of beautiful fish. You're not trying to chase the fish. You're not trying to catch the fish. You're just calmly watching them. Your back is the ocean and the sensations you're feeling are those fish. All you have to do is observe. I'm just a friendly sea turtle swimming nearby. A friendly, talking sea turtle. Okay, I may have taken this analogy too far" (Gordon and Ziv, forthcoming).

Picturing the therapist as a talking turtle is a little silly, and that's the point. This is all about lightening the mood. The goal is to help the patient observe their physical sensations with lightness and curiosity.

In addition to leading the patient in somatic tracking exercises in-session, the therapist guides the patient on how to practice on their own. Patients are guided on when to engage in somatic tracking and when to abstain, based on the level of pain intensity. Often during a somatic tracking exercises, patients are able to get a "corrective experience". If they sit/stand/walk/bend with little to no pain, it further reinforces that the pain is due to central processes, and that there is nothing wrong with their bodies. This frees them up to engage in previously fear positions and activities. Subsequently when the pain does arise, instead of responding with fear, frustration, or despair, the patient is able to authentically reappraise the pain as a misinterpretation by their brain, as opposed to a reflection of tissue damage in their body.

IV. Addressing other emotional threats

© 2021 Ashar YK et al. *JAMA Psychiatry.*

When we are in a state of high alert, we are more likely to interpret *everything* through a lens of danger. Loud noises will make us jump, light touches will cause us to recoil, and sensations in our body are more likely to be experienced as painful.

PRT thus aims to lower a person's overall threat level. This can include helping someone process threatening emotions, a history of trauma, difficult relationships, and more. As overall levels of fear and stress decrease, the brain is more likely to interpret signals from the body as safe, leading to a reduction in pain. Techniques for expressing, disclosing, and processing difficult emotions from several relevant therapeutic approaches (e.g., emotional awareness and expression therapy), can be used for this component of PRT.

Relatedly, patients often have a tendency to engage in psychologically destructive behaviors, such as self-criticism, putting pressure on themselves, and scaring themselves. These behaviors can further communicate messages of danger to the brain, thus increasing susceptibility to pain. As part of PRT, the therapist helps the patient identify such psychologically destructive behaviors and develop the skills to intervene on their own behalf.

V. Gravitating more generally to positive feelings and sensations

In addition to reducing the patient's overall threat level, PRT also aims to increase an overall feeling of safety. Pain patients have become so conditioned to gravitating toward negative and unpleasant sensations in their body that they often focus on many things through a lens of danger (sensations, emotions, even their own selves). One of the goals of PRT is to help the patient more globally shift from "danger mode" to "safety mode."

The therapist can help the patient attend to pleasant sensations in their body (e.g., the breath) through a lens of positivity:

- "See if you can pay attention to the physical sensation of the breath. The air is cool as it comes in, and warm as it goes out. You don't want to scrutinize it like the way you study for an exam, you're simply watching it with a sense of effortlessness and ease, like when you're lying back in a meadow and watching the clouds pass above. And see if you can actually enjoy this pleasant feeling in your body."

© 2021 Ashar YK et al. *JAMA Psychiatry.*

As the patient gets practice leaning into positive sensations through a lens of safety, it increases their capacity to attend to aversive sensations through a lens of safety as well.

Likewise, the therapist can help the patient gravitate toward other positive emotional states. For example, techniques for increasing self-compassion and gratitude can help further generate that shift from “danger” to “safety”.

Ultimately as the patient develops the skills of attending to internal and external stimuli through a lens of safety and promoting a more general felt-sense of safety, it will support reappraising pain sensations as safe as well.

Conclusions

In PRT, the first focus is on education, evidence-gathering, and reappraising the pain sensations as non-dangerous (typically using somatic tracking). Other components of PRT are then engaged as needed (e.g., addressing threatening emotions, learning to attend to positive sensations). We then return the focus of treatment to the pain sensations as soon as appropriate.

© 2021 Ashar YK et al. *JAMA Psychiatry.*

eAppendix 3. PRT treatment fidelity checklist

Please check all of the following therapist activities / behavior that were present in the session that you reviewed:

___ 1. *Educating patient about the brain origins and reversibility of pain*

Definition: Communicating to a patient a conceptual model that beliefs, attributions and emotions surrounding pain, and the neural pathways that support them, are the primary cause of pain, rather than tissue damage.

- Teaching patient to understand that pain is due to brain processes, and there is nothing “wrong” with the body
- Teaching patient that fear and avoidance of the pain perpetuate the pain, and the pain can be reduced or eliminated by reattributing the pain to the brain rather than body

___ 2. *Helping patient gather personal evidence about the brain origins and reversibility of their pain*

Definition: The therapist engages with the patient to identify evidence supporting the assessment of their pain as brain-based and reversible. This is a personalized process, focused on features of the particular patient, such as injury history, pain sensation fluctuations, medical test results, personality style, adverse childhood experiences, etc. This should be coded as present for the process of considering evidence, regardless of whether conclusive evidence is found or endorsed by patient.

- Identifying personal examples that support pain as brain-generated
- Helping patient to recognize their experiences as evidence that pain is caused by brain

___ 3. *Attending to and appraising pain sensations (“somatic tracking”)*

Definition: The therapist directs the patient’s attention to pain sensations and promotes the reappraisal of those sensations as safe and/or brain-generated using cognitive, mindfulness-based, somatic, or other techniques. This can be done while the patient is sitting or while the patient is engaging in a different pain or fear-inducing posture or movement (e.g., bending, standing, walking, etc.).

___ 4. *Processing of difficult emotions and external stressors*

Definition: The therapist helps the patient access, disclose, explore, understand, and/or navigate difficult emotions and stress that may contribute to pain.

-Helping patient address negative emotions related to pain (fear, frustration, sadness, etc.)

© 2021 Ashar YK et al. *JAMA Psychiatry.*

-Helping patient explore/discuss/understand difficult emotions not directly related to the pain
-Helping patient to recognize and reduce self-directed, psychologically destructive
feelings (e.g., self-criticism, putting pressure on self, scaring themselves, etc.)
-Helping patient to navigate external stressors (e.g., relating to work, school, relationships, etc.)

___ 5. *Generating positive emotional states*

Definition: Therapist helps the patient experience positive affect as part of the overall goal of helping patient shift from “danger-focused” to “safety focused” and provides positive affect to facilitate engaging in somatic tracking, behavioral engagement, or processing of difficult emotions.

- Guiding patient to attend to positive (pleasant) sensations in their body.
- Helping patient develop a playful and/or curious attitude towards sensations
- Using humor to help engage and motivate patient and help patient feel safer
- Helping patient increase self-compassion or other positive emotional states

___6. *Prescribing or evaluating home practice*

Definition: Therapist assigns between-session activities or discusses between-session activities from the previous session, such as review of pain education materials that support concepts used in PRT, engaging in somatic tracking, experiencing or amplifying positive sensations, moving/behaving in feared and/or painful ways. The therapist may also direct activities *not* to engage if they are deemed overly painful or scary for the current stage of treatment.

- Encouraging and prescribing the patient to do the above between sessions
- Discussing with patients their at-home activities

© 2021 Ashar YK et al. *JAMA Psychiatry.*

eReferences

1. Leibowitz KA, Hardebeck EJ, Goyer JP, Crum AJ. The Role of Patient Beliefs in Open-Label Placebo Effects. *Heal Psychol*. 2019;38(7):613-622. doi:10.1037/hea0000751
2. Locher C, Nascimento AF, Kirsch I, Kossowsky J, Meyer A, Gaab J. Is the rationale more important than deception? A randomized controlled trial of openlabel placebo analgesia. *Pain*. 2017;0(0):1. doi:10.1097/j.pain.0000000000001012
3. Kaptchuk TJ, Friedlander E, Kelley JM, et al. Placebos without deception: A randomized controlledtrial in irritable bowel syndrome. *PLoS One*. 2010;5(12):1-7. doi:10.1371/journal.pone.0015591
4. Locher C, Nascimento AF, Kirsch I, Kossowsky J, Meyer A, Gaab J. Is the rationale more important than deception? A randomized controlled trial of openlabel placebo analgesia. *Pain*. 2017;0(0):1. doi:10.1097/j.pain.0000000000001012
5. Lembo A, Kelley JM, Nee J, et al. Open-label placebo vs double-blind placebo for irritable bowel syndrome: a randomized clinical trial. *Pain*. Published online 2021. doi:10.1097/j.pain.0000000000002234
6. Carvalho C, Caetano JM, Cunha L, Rebouta P, Kaptchuk TJ, Kirsch I. Open-label placebo treatment in chronic low back pain. *Pain*. 2016;0(0):1. doi:10.1097/j.pain.0000000000000700
7. Pek J, Flora DB. Reporting effect sizes in original psychological research: A discussion and tutorial. *Psychol Methods*. 2018;23(2):208-225. doi:10.1037/met0000126
8. Dworkin RH, Turk DC, Farrar JT, et al. Core outcome measures for chronic pain clinical trials: IMMPACT recommendations. *Pain*. 2005;113(1-2):9-19. doi:10.1016/j.pain.2004.09.012
9. Ramasamy A, Martin ML, Blum SI, et al. Assessment of Patient-Reported Outcome Instruments to Assess Chronic Low Back Pain. *Pain Med*. Published online 2017:1-13. doi:10.1093/pm/pnw357
10. Cleeland C, Ryan K. *The Brief Pain Inventory*. Pain Research Group; 1991.
11. Fairbank JC, Couper J, Davies JB, O'Brien JP. The Oswestry low back pain disability questionnaire. *Physiotherapy*. 1980;66(8):271-273. doi:PMID: 6450426
12. Thompson ER, Wanberg CR, Zhang Z, et al. Development and Validation of an Internationally Reliable Short-Form of the Positive and Negative Affect Schedule (PANAS). *J Cross Cult Psychol*. 2007;38(2):227-242. doi:10.1177/0022022106297301
13. Stone AA, Broderick JE, Junghaenel DU, Schneider S, Schwartz JE. PROMIS fatigue, pain intensity, pain interference, pain behavior, physical function, depression, anxiety, and anger scales demonstrate ecological validity. *J Clin Epidemiol*. Published online November 25, 2015. doi:10.1016/j.jclinepi.2015.08.029
14. Licciardone J, Worzer WE, Hartzell MM, Kishino N, Gatchel RJ. An Overview of the Patient-Reported Outcomes Measurement Information System (PROMIS) for

© 2021 Ashar YK et al. *JAMA Psychiatry.*

Assessing Chronic Low Back Pain Patients. *J Appl Biobehav Res*. Published online 2017:1-22. doi:10.1111/jabr.12057
15. Sullivan MJL, Bishop SR, Pivik J. The Pain Catastrophizing Scale: Development and validation. *Psychol Assess*. 1995;7(4):524-532. doi:10.1037/1040-3590.7.4.524
16. Tkachuk GA, Harris CA. Psychometric properties of the tampa scale for kinesiophobia-11 (TSK-11). *J Pain*. 2012;13(10):970-977. doi:10.1016/j.jpain.2012.07.001
17. Woby SR, Roach NK, Urmston M, Watson PJ. Psychometric properties of the TSK-11: A shortened version of the Tampa Scale for Kinesiophobia. *Pain*. 2005;117(1-2):137-144. doi:10.1016/j.pain.2005.05.029
18. Jensen MP, Keefe FJ, Lefebvre JC, Romano JM, Turner JA. One- and two-item measures of pain beliefs and coping strategies. *Pain*. 2003;104(3):453-469. doi:10.1016/S0304-3959(03)00076-9
19. Robinson SM, Sobell LC, Sobell MB, Leo GI. Reliability of the Timeline Followback for cocaine, cannabis, and cigarette use. *Psychol Addict Behav*. 2014;28(1):154-162. doi:10.1037/a0030992
20. Atkinson MJ, Sinha A, Hass SL, et al. Validation of a general measure of treatment satisfaction, the Treatment Satisfaction Questionnaire for Medication (TSQM), using a national panel study of chronic disease. *Health Qual Life Outcomes*. 2004;2(1):12. doi:10.1186/1477-7525-2-12
21. Hoffman BM, Papas RK, Chatkoff DK, Kerns RD. Meta-analysis of psychological interventions for chronic low back pain. *Health Psychol*. 2007;26(1):1-9. doi:10.1037/0278-6133.26.1.1
22. Xiao L, Yank V, Ma J. Algorithm for balancing both continuous and categorical covariates in randomized controlled trials. *Comput Methods Programs Biomed*. 2012;108(3):1185-1190. doi:10.1016/j.cmpb.2012.06.001
23. Ma Z, Hu F. Balancing continuous covariates based on Kernel densities. *Contemp Clin Trials*. 2013;34(2):262-269. doi:10.1016/j.cct.2012.12.004
24. Lin Y, Su Z. Balancing continuous and categorical baseline covariates in sequential clinical trials using the area between empirical cumulative distribution functions. *Stat Med*. 2012;31(18):1961-1971. doi:10.1002/sim.5363
25. Hentschke H, Stüttgen MC. Computation of measures of effect size for neuroscience data sets. *Eur J Neurosci*. 2011;34(12):1887-1894. doi:10.1111/j.1460-9568.2011.07902.x
26. Lemmer G, Gollwitzer M. The "true" indirect effect won't (always) stand up: When and why reverse mediation testing fails. *J Exp Soc Psychol*. 2017;69:144-149. doi:10.1016/j.jesp.2016.05.002
27. Thoemmes F. Reversing Arrows in Mediation Models Does Not Distinguish Plausible Models. *Basic Appl Soc Psych*. 2015;37(4):226-234. doi:10.1080/01973533.2015.1049351
28. Esteban O, Markiewicz CJ, Blair RW, et al. fMRIPrep: a robust preprocessing pipeline for functional MRI. *Nat Methods*. 2019;16(1):111-116. doi:10.1038/s41592-018-0235-4
29. Gorgolewski K, Burns CD, Madison C, et al. Nipype: A Flexible, Lightweight and Extensible Neuroimaging Data Processing Framework in Python. *Front*

Neuroinform. 2011;5:13. doi:10.3389/fninf.2011.00013
30. Tustison NJ, Avants BB, Cook PA, et al. N4ITK: Improved N3 Bias Correction. *IEEE Trans Med Imaging*. 2010;29(6):1310-1320. doi:10.1109/TMI.2010.2046908
31. Reuter M, Rosas HD, Fischl B. Highly accurate inverse consistent registration: A robust approach. *Neuroimage*. 2010;53(4):1181-1196. doi:10.1016/J.NEUROIMAGE.2010.07.020
32. Fonov V, Evans A, McKinstry R, Almli C, Collins D. Unbiased nonlinear average age-appropriate brain templates from birth to adulthood. *Neuroimage*. 2009;47:S102. doi:10.1016/S1053-8119(09)70884-5
33. Cox RW, Hyde JS. Software tools for analysis and visualization of fMRI data. *NMR Biomed*. 1997;10(4-5):171-178. doi:10.1002/(SICI)1099-1492(199706/08)10:4/5<171::AID-NBM453>3.0.CO;2-L
34. Jenkinson M, Smith S. A global optimisation method for robust affine registration of brain images. *Med Image Anal*. 2001;5(2):143-156. doi:10.1016/S1361-8415(01)00036-6
35. Greve DN, Fischl B. Accurate and robust brain image alignment using boundary-based registration. *Neuroimage*. 2009;48(1):63-72. doi:10.1016/J.NEUROIMAGE.2009.06.060
36. Lanczos C. Evaluation of Noisy Data. *J Soc Ind Appl Math Ser B Numer Anal*. 1964;1(1):76-85. doi:10.1137/0701007
37. Power JD, Barnes KA, Snyder AZ, Schlaggar BL, Petersen SE. Spurious but systematic correlations in functional connectivity MRI networks arise from subject motion. *Neuroimage*. 2012;59(3):2142-2154. doi:10.1016/j.neuroimage.2011.10.018
38. Power JD, Lynch CJ, Silver BM, Dubin MJ, Martin A, Jones RM. Distinctions among real and apparent respiratory motions in human fMRI data. *Neuroimage*. 2019;201(July):116041. doi:10.1016/j.neuroimage.2019.116041
39. Snellenberg V. Motion denoising of multiband resting state functional connectivity MRI data: An improved volume censoring method. 2019;c.
40. Behzadi Y, Restom K, Liau J, Liu TT. A component based noise correction method (CompCor) for BOLD and perfusion based fMRI. *Neuroimage*. 2007;37(1):90-101. doi:10.1016/j.neuroimage.2007.04.042
41. Ciric R, Rosen AFG, Erus G, et al. Mitigating head motion artifact in functional connectivity MRI. *Nat Protoc*. 2018;13(12):2801-2826. doi:10.1038/s41596-018-0065-y
42. Cole MW, Bassett DS, Power JD, Braver TS, Petersen SE. Intrinsic and task-evoked network architectures of the human brain. *Neuron*. 2014;83(1):238-251. doi:10.1016/j.neuron.2014.05.014
43. Dale J, Zhou H, Zhang Q, et al. Scaling Up Cortical Control Inhibits Pain. *Cell Rep*. 2018;23(5):1301-1313. doi:10.1016/j.celrep.2018.03.139
44. Lee M, Manders TR, Eberle SE, et al. Activation of Corticostriatal Circuitry Relieves Chronic Neuropathic Pain. 2015;35(13):5247-5259. doi:10.1523/JNEUROSCI.3494-14.2015
45. Schwartz N, Miller C, Fields HL. Cortico-Accumbens Regulation of Approach-Avoidance Behavior Is Modified by Experience and Chronic Pain. *Cell Rep*. 2017;19(8):1522-1531. doi:10.1016/j.celrep.2017.04.073

© 2021 Ashar YK et al. *JAMA Psychiatry.*

46. Woo C-W, Roy M, Buhle JT, Wager TD. Distinct Brain Systems Mediate the Effects of Nociceptive Input and Self-Regulation on Pain. Posner M, ed. *PLoS Biol*. 2015;13(1):e1002036. doi:10.1371/journal.pbio.1002036
47. Hua T, Chen B, Lu D, et al. General anesthetics activate a potent central pain-suppression circuit in the amygdala. *Nat Neurosci*. Published online 2020. doi:10.1038/s41593-020-0632-8
48. Corder G, Ahanonu B, Grewe BF, Wang D, Schnitzer MJ, Scherrer G. An amygdalar neural ensemble that encodes the unpleasantness of pain. *Science (80-)*. 2019;363(6424):276-281. doi:10.1126/science.aap8586
49. Tracey I. Getting the pain you expect: mechanisms of placebo, nocebo and reappraisal effects in humans. *Nat Med*. 2010;16(11):1277-1283. doi:10.1038/nm.2229
50. Bushnell MC, Ceko M, Low L a. Cognitive and emotional control of pain and its disruption in chronic pain. *Nat Rev Neurosci*. 2013;14(7):502-511. doi:10.1038/nrn3516
51. Napadow V, Harris RE. What has functional connectivity and chemical neuroimaging in fibromyalgia taught us about the mechanisms and management of “centralized” pain? *Arthritis Res Ther*. 2014;16(4):1-8. doi:10.1186/s13075-014-0425-0
52. Baliki MN, Apkarian AV. Nociception, Pain, Negative Moods, and Behavior Selection. *Neuron*. 2015;87(3):474-491. doi:10.1016/j.neuron.2015.06.005
53. Yeo BTT, Krienen FM, Sepulcre J, et al. The organization of the human cerebral cortex estimated by intrinsic functional connectivity. *J Neurophysiol*. 2011;106:1125-1165. doi:10.1152/jn.00338.2011.
54. Pauli WM, Nili AN, Tyszka JM. A high-resolution probabilistic in vivo atlas of human subcortical brain nuclei. *Sci Data*. 2018;5:180063. https://doi.org/10.1038/sdata.2018.63
55. Glasser MF, Coalson TS, Robinson EC, et al. A multi-modal parcellation of human cerebral cortex. *Nature*. 2016;536(7615):171-178. doi:10.1038/nature18933
56. Martucci KT, Shirer WR, Bagarinao E, et al. The posterior medial cortex in urologic chronic pelvic pain syndrome: Detachment from default mode network - A resting-state study from the MAPP Research Network. *Pain*. 2015;156(9):1755-1764. doi:10.1097/j.pain.0000000000000238
57. Napadow V, Kim J, Clauw DJ, Harris RE. Decreased intrinsic brain connectivity is associated with reduced clinical pain in fibromyalgia. *Arthritis Rheum*. 2012;64(7):2398-2403. doi:10.1002/art.34412 [doi]
58. Mawla I, Harris RE, Napadow V. Greater Somatosensory Afference with Acupuncture Increases Primary Somatosensory Connectivity and alleviates Fibromyalgia Pain via Insular GABA. In: *Pain Research Centre Data Blitz*. ; 2020.
59. Lazaridou A, Kim J, Cahalan CM, et al. Effects of Cognitive-behavioral Therapy (CBT) on Brain Connectivity Supporting Catastrophizing in Fibromyalgia. *Clin J Pain*. Published online 2016. doi:10.1097/AJP.0000000000000422
60. Kim J, Mawla I, Kong J, et al. Somatotopically specific primary somatosensory connectivity to salience and default mode networks encodes clinical pain. *Pain*. 2019;160(7):1594-1605. doi:10.1097/j.pain.0000000000001541

© 2021 Ashar YK et al. *JAMA Psychiatry.*

61. Kong J, Spaeth RB, Wey H-Y, et al. S1 is associated with chronic low back pain: a functional and structural MRI study. *Mol Pain*. 2013;9(1):43. doi:10.1186/1744-8069-9-43
62. Parkes L, Fulcher B, Yücel M, Fornito A. NeuroImage An evaluation of the ef fi cacy , reliability , and sensitivity of motion correction strategies for resting-state functional MRI. *Neuroimage*. 2018;171(December 2017):415-436. doi:10.1016/j.neuroimage.2017.12.073
63. Brinjikji W, Luetmer PH, Comstock B, et al. Systematic literature review of imaging features of spinal degeneration in asymptomatic populations. *Am J Neuroradiol*. 2015;36(4):811-816. doi:10.3174/ajnr.A4173
64. Keefe FJ, Caldwell DS, Williams DA, et al. Pain coping skills training in the management of osteoarthritic knee pain: A comparative study. *Behav Ther*. 1990;21(1):49-62. doi:10.1016/S0005-7894(05)80188-1
65. Thorn BE, Eyer JC, Van Dyke BP, et al. Literacy-adapted cognitive behavioral therapy versus education for chronic pain at low-income clinics a randomized controlled trial. *Ann Intern Med*. 2018;168(7):471-480. doi:10.7326/M17-0972
66. Cherkin DC, Sherman KJ, Balderson BH, et al. Effect of Mindfulness-Based Stress Reduction vs Cognitive Behavioral Therapy or Usual Care on Back Pain and Functional Limitations in Adults With Chronic Low Back Pain. *JAMA*. 2016;315(12):1240. doi:10.1001/jama.2016.2323
67. McCracken LM, Vowles KE. Acceptance and commitment therapy and mindfulness for chronic pain: Model, process, and progress. *Am Psychol*. 2014;69(2).
68. Kabat-Zinn J, Lipworth L, Burney R. The clinical use of mindfulness meditation for the self-regulation of chronic pain. *J Behav Med*. 1985;8(2):163-190. doi:10.1007/BF00845519
69. Garland EL, Manusov EG, Froeliger B, Kelly A, Williams JM, Howard MO. Mindfulness-oriented recovery enhancement for chronic pain and prescription opioid misuse: results from an early-stage randomized controlled trial. *J Consult Clin Psychol*. 2014;82(3):448-459. doi:10.1037/a0035798
70. Moseley GL, Butler DS. Fifteen Years of Explaining Pain: The Past, Present, and Future. *J Pain*. 2015;16(9):807-813. doi:10.1016/j.jpain.2015.05.005
71. Clarke CL, Ryan CG, Martin DJ. Pain neurophysiology education for the management of individuals with chronic low back pain: A systematic review and meta-analysis. *Man Ther*. 2011;16(6):544-549. doi:10.1016/j.math.2011.05.003
72. Traeger AC, Lee H, Hübscher M, et al. Effect of Intensive Patient Education vs Placebo Patient Education on Outcomes in Patients with Acute Low Back Pain: A Randomized Clinical Trial. *JAMA Neurol*. 2019;76(2):161-169. doi:10.1001/jamaneurol.2018.3376
73. Kleine-borgmann J, Schmidt K, Hellmann A, Bingel U. Effects of open-label placebo on pain , functional disability , and spine mobility in patients with chronic back pain : a randomized controlled trial. 2019;160(12).
74. Tuttle AH, Tohyama S, Ramsay T, et al. *Increasing Placebo Responses over Time in U.S. Clinical Trials of Neuropathic Pain*.; 2015. doi:10.1097/j.pain.0000000000000333
75. Hróbjartsson A, Gøtzsche PC. Placebo interventions for all clinical conditions.

© 2021 Ashar YK et al. *JAMA Psychiatry.*

Cochrane Database Syst Rev. 2010;CD003974(1). doi:10.1002/14651858.CD003974.pub3.www.cochranelibrary.com
76. Peerdeman KJ, van Laarhoven AIM, Keij SM, et al. Relieving patients' pain with expectation interventions. *Pain*. 2016;157(6):1. doi:10.1097/j.pain.0000000000000540
77. Dahlhamer J, Lucas J, Zelaya, C, et al. Prevalence of Chronic Pain and High-Impact Chronic Pain Among Adults — United States, 2016. *MMWR Morb Mortal Wkly Rep*. 2018;67(36):1001-1006. doi:10.15585/mmwr.mm6736a2
78. Kallmes DF, Comstock BA, Heagerty PJ, et al. A Randomized Trial of Vertebroplasty for Osteoporotic Spinal Fractures. *N Engl J Med*. 2009;361(6):569-579. doi:10.1056/nejmoa0900563
79. McAlindon TE, LaValley MP, Harvey WF, et al. Effect of intra-articular triamcinolone vs saline on knee cartilage volume and pain in patients with knee osteoarthritis a randomized clinical trial. *JAMA*. 2017;317(19):1967-1975. doi:10.1001/jama.2017.5283
80. Fitzcharles MA, Cohen SP, Clauw DJ, Littlejohn G, Usui C, Häuser W. Nociplastic pain: towards an understanding of prevalent pain conditions. *Lancet*. 2021;397(10289):2098-2110. doi:10.1016/S0140-6736(21)00392-5
81. Seminowicz DA, Moayedi M. The Dorsolateral Prefrontal Cortex in Acute and Chronic Pain. *J Pain*. 2017;18(9):1027-1035. doi:10.1016/j.jpain.2017.03.008
82. Wasan AD, Loggia ML, Chen LQ, Napadow V, Kong J, Gollub RL. Neural correlates of chronic low back pain measured by arterial spin labeling. *Anesthesiology*. 2011;115(2):364-374. doi:10.1097/ALN.0b013e318220e880
83. Lee J, Mawla I, Kim J, et al. Machine learning-based prediction of clinical pain using multimodal neuroimaging and autonomic metrics. *Pain*. 2019;160(3):550-560. doi:10.1097/j.pain.0000000000001417
84. Ashar YK, Chang LJ, Wager TD. Brain Mechanisms of the Placebo Effect: An Affective Appraisal Account. *Annu Rev Clin Psychol*. 2017;13(1):73-98. doi:10.1146/annurev-clinpsy-021815-093015
85. Koban L, Gianaros PJ, Kober H, Wager TD. The self in context: brain systems linking mental and physical health. *Nat Rev Neurosci*. Published online 2021:1-14. doi:10.1038/s41583-021-00446-8
86. Zeidan F, Salomons T, Farris SR, et al. Neural mechanisms supporting the relationship between dispositional mindfulness and pain. *Pain*. 2018;159(12):2477-2485. doi:10.1097/j.pain.0000000000001344
87. Lee J, Protsenko E, Lazaridou A, et al. Encoding of Self-Referential Pain Catastrophizing in the Posterior Cingulate Cortex in Fibromyalgia. *Arthritis Rheumatol*. 2018;70(8):1308-1318. doi:10.1002/art.40507
88. Lumley MA, Schubiner H. Psychological Therapy for Centralized Pain : An Integrative Assessment and Treatment Model. 2019;(March):114-124. doi:10.1097/PSY.0000000000000654
89. Abbass A, Schubiner H. *Hidden from View.: A Clinician's Guide to Psychophysiologic Disorders.* Psychophysiologic Press; 2018.

© 2021 Ashar YK et al. *JAMA Psychiatry.*
