# Supplementary material for: Effect of Pain Reprocessing Therapy vs Placebo and Usual Care for Patients With Chronic Back Pain: A Randomized Clinical Trial
Source: JAMA Psychiatry. 2021 Sep 29;79(1):1–12. doi: 10.1001/jamapsychiatry.2021.2669 (PMC8482298; doi:10.1001/jamapsychiatry.2021.2669)
Supplement: Supplement 3. — Data sharing statement. [file jamapsychiatry-e212669-s003.pdf]

Data Sharing Statement

Ashar. Effect of Pain Reprocessing Therapy vs Placebo and Usual Care for Patients With Chronic Back Pain. *JAMA Psychiatry*. Published September 29, 2021. doi:10.1001/jamapsychiatry.2021.2669

Data
Data available: Yes
Data types: Deidentified participant data
How to access data: De-identified patient-reported outcomes and single-subject fMRI statistical parameter maps will be shared via figshare:
https://figshare.com/s/1840dc4c0e236a7072ca
When available: With publication

Supporting Documents
Document types: None

Additional Information
Who can access the data: public
Types of analyses: for any purpose
Mechanisms of data availability: publicly available
